# Low-Coordinate Magnesium Sulfide and Selenide Complexes

**DOI:** 10.1021/acs.inorgchem.3c02132

**Published:** 2023-09-25

**Authors:** Stuart Burnett, Rochelle Ferns, David B. Cordes, Alexandra M. Z. Slawin, Tanja van Mourik, Andreas Stasch

**Affiliations:** † EaStCHEM School of Chemistry, University of St. Andrews, North Haugh, St. Andrews KY16 9ST, United Kingdom

## Abstract

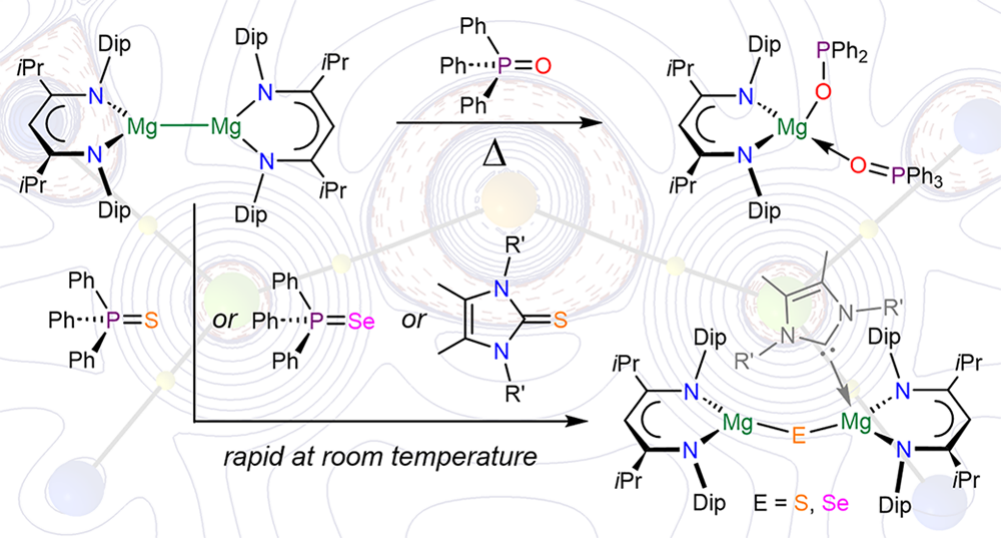

The reactions of [{(^iPrDip^NacNac)Mg}_2_] **1** (^iPrDip^nacnac = HC(iPrCNDip)_2_) with Ph_3_P=O at 100 °C afforded the phosphinate complex [(^iPrDip^NacNac)Mg(OPPh_3_)(OPPh_2_)] **3**. Reactions of **1** with Ph_3_P=E (E = S, Se) proceeded rapidly at room temperature to low-coordinate chalcogenide complexes [{(^iPrDip^NacNac)Mg}_2_(μ-S)] **4** and [{(^iPrDip^NacNac)Mg}_2_(μ-Se)] **5**, respectively. Similarly, reactions of ^R^NHC=S ((MeCNR)_2_C=S with R = Me, Et, or *i*Pr) with **1** afforded NHC adducts of magnesium sulfide complexes, [{(^iPrDip^NacNac)Mg(^R^NHC)}(μ-S){Mg(^iPrDip^NacNac)}] **6**, that could alternatively be obtained by adding the appropriate ^R^NHC to sulfide complex **4**. Complex **4** reacted with 1-adamantylazide (AdN_3_) to give [{(^iPrDip^NacNac)Mg}_2_(μ-SN_3_Ad)] **7** and can form various simple donor adducts in solution, of which [(^iPrDip^NacNac)Mg(OAd)}_2_(μ-S)] **8a** (OAd = 2-adamantanone) was structurally characterized. The nature of the ionic Mg–E–Mg unit is described by solution and solid-state studies of the complexes and by DFT computational investigations.

## Abstract 2

The synthesis of the first well-defined magnesium sulfide complexes is reported from the reaction of a dimagnesium(I) complex with triphenylphosphine sulfide or imidazole-2-thiones. A low-coordinate magnesium selenide complex is also described. The structures and properties of the compound classes were investigated by X-ray diffraction, NMR spectroscopy, and DFT computational studies.

## Introduction

**Figure 1:**
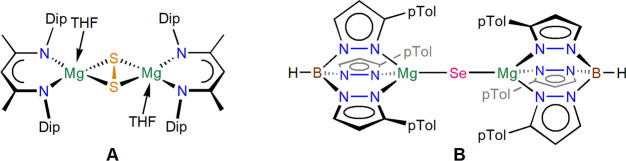
Compounds **A** and **B**.

Well-defined complexes of inorganic fragments in low-coordination modes are expected to show significantly different properties and a higher reactivity compared with those of solid bulk materials. Metal chalcogenide materials are of interest for various energy-related areas, and thus many synthetic methods have been successfully applied to various nanostructured materials and bulk solids.^1−4^ In the field of well-defined molecular compounds with main group element–chalcogen bonds,^6,7^ including heavier carbonyl analogues,^8,9^ significant advances have been made in recent years regarding their controlled synthesis, structure, and bonding, but many challenges remain, especially for those of the early main group metals. For magnesium, oxide complexes with an LMgOMgL (L = anionic ligand) unit and low-coordinate oxide ions^10−13^ were predominantly obtained by reactions of “high-energy” dimagnesium(I) species^14−16^ with nitrous oxide (N_2_O). In addition, some molecules with low-coordinate BeOBe units have been structurally characterized.^17,18^ Well-defined alkaline earth metal complexes of heavier chalcogenides are very rare, and a majority of complexes with magnesium and sulfur contacts stem from compounds containing sulfur as part of larger anionic ligands (e.g., in magnesium thiolate complexes).^19−25^ A rare magnesium disulfide complex (**A**), see Figure 1, was reported by Ren and Gu from the reaction of a Mg^I^ mimic and S_8_, plus related work for Ca.^26,27^ Ghosh and Parkin reported a structurally characterized magnesium selenide complex **B** (Figure 1) that was prepared via an organomagnesium species and H_2_Se, and this work also featured hydroselenide (biselenide) and a related bisulfide species.^28^ For the related metal zinc, few complexes with bridging low-coordinate chalcogenide ions are known^29−32^ plus a unique anionic complex with a terminal Zn–S bond.^33^ Here we report on the chemistry of low-coordinate β-diketiminate magnesium sulfide complexes and one related magnesium selenide complex.

## Results and Discussion

### Synthesis

**Scheme 1:**
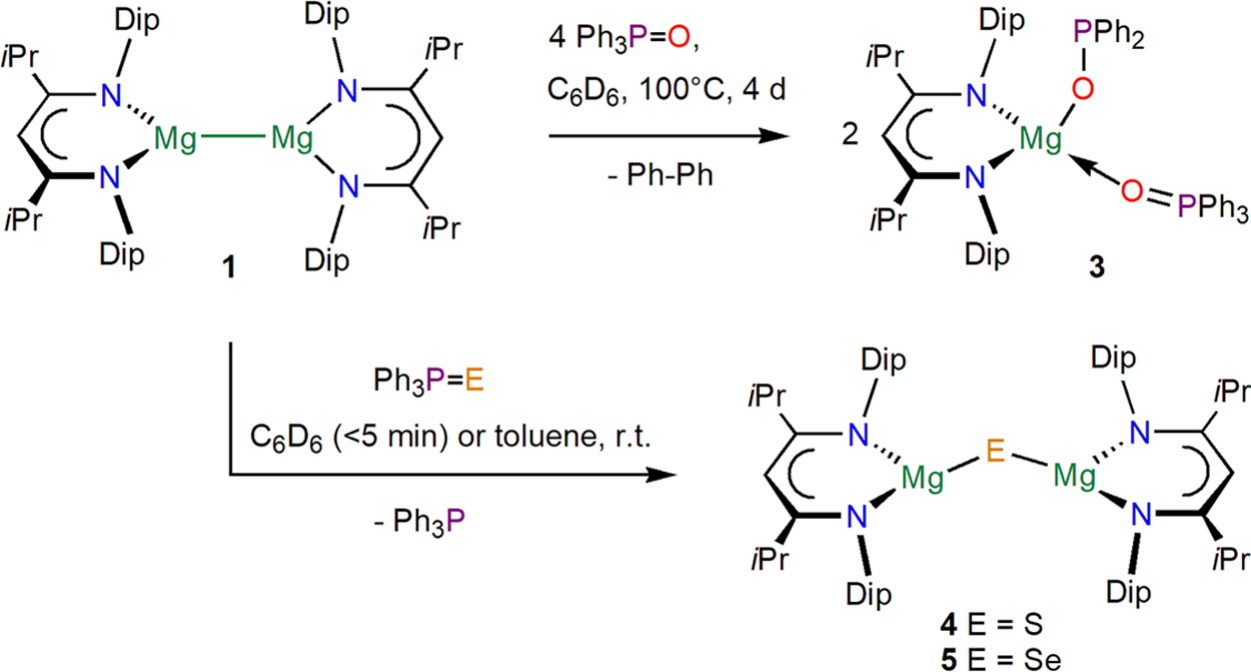
Syntheses of Complexes **3**–**5**

**Figure 2:**
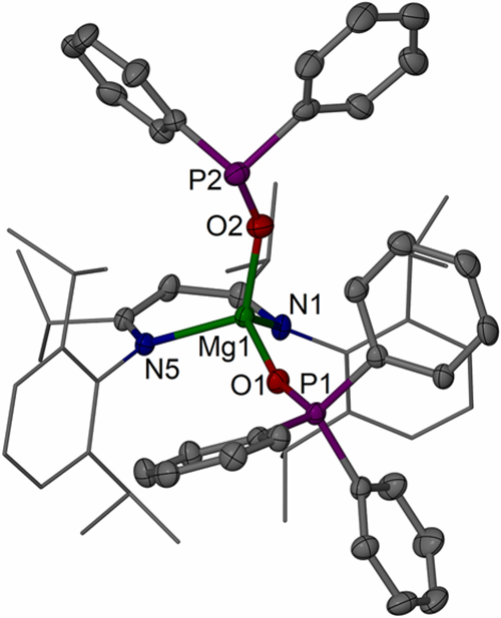
Molecular structure (30% thermal ellipsoids) of [(^iPrDip^NacNac)Mg(OPPh_3_)(OPPh_2_)] **3**·C_6_H_6_. Dip and *i*Pr groups are shown as wireframe. Only the major part of the disordered Ph_2_P group is shown. Hydrogen atoms are omitted. Selected bond lengths (Å) and angles (deg): P1–O1 1.4972(13), P2–O2 1.5459(17), Mg1–O2 1.9000(16), Mg1–O1 1.9243(14), Mg1–N1 2.0511(17), Mg1–N5 2.0767(16); O2–Mg1–O1 109.13(7), N1–Mg1–N5 95.12(6), P1–O1–Mg1 162.83(9), P2–O2–Mg1 143.26(11).

Previously, reactions of dimagnesium(I) complexes with N_2_O afforded low-coordinate oxide complexes, but these reactions can be plagued by the formation of significant quantities of hydroxide byproducts.^10,11,13^ With the target of a convenient synthesis of LMgEMgL complexes, E = group 16 elements, we studied the reactions of the dimagnesium(I) complex [{(^*i*PrDip^NacNac)Mg}_2_] **1**(13) with Ph_3_P=E (E = O, S, Se). The addition of one equivalent of Ph_3_P=O to a yellow benzene-*d*_6_ solution of **1** at ambient temperature showed no reaction as judged by ^1^H and ^31^P{^1^H} NMR spectroscopy. Heating the solution to 60 °C for 3 days suggested only minimal conversion, and heating to 100 °C afforded a slow reaction, a partial conversion of **1**, and a color change to orange-red but no formation of the magnesium oxide complex [{(^iPrDip^NacNac)Mg}_2_(μ-O)] **2**.^13^ Reacting [{(^*i*PrDip^NacNac)Mg}_2_] **1** with four equivalents of Ph_3_P=O at 100 °C for 2 days afforded the full conversion of **1** to one main *β-*diketiminate-containing complex with two different ^31^P{^1^H} NMR singlets, indicative of a coordinated triphenylphosphinoxide donor (Ph_3_PO···Mg, 36.8 ppm) and a diphenylphosphinite ligand (Ph_2_PO–Mg, 85.8 ppm), [(^iPrDip^NacNac)Mg(OPPh_2_)(OPPh_3_)] **3**; see Scheme 1 and Figure 2 for a molecular structure. Biphenyl was detected by ^1^H NMR spectroscopy as a byproduct from this reaction. Complex **3** was isolated from *n*-hexane in 44% isolated yield as a colorless solid that often appears red in earlier crops due to the intense color in solution. For the related reaction of sodium dispersion with Ph_3_PO in THF, the generation of Ph_2_PONa is observed and the formation of NaPh as a byproduct has been suggested due to the onward reactivity with sodium diphenylphosphinite to C–C coupled sodium 5*H*-benzo[b]phosphindol-5-olate.^34,35^ The generated NaPh has been shown to then react with the THF solvent to form sodium vinyl alkoxide in this system.^34,35^ The relatively harsh reaction conditions for the formation of **3** and the color of the solution and the formation of biphenyl as the byproduct suggest that the reaction proceeds via donor adducts of magnesium(I) complexes with elongated Mg–Mg bonds^14−16,36^ (e.g., Ph_3_PO bisadducts of **1**) and could also point to the formation of radicals during the process.

**Figure 3:**
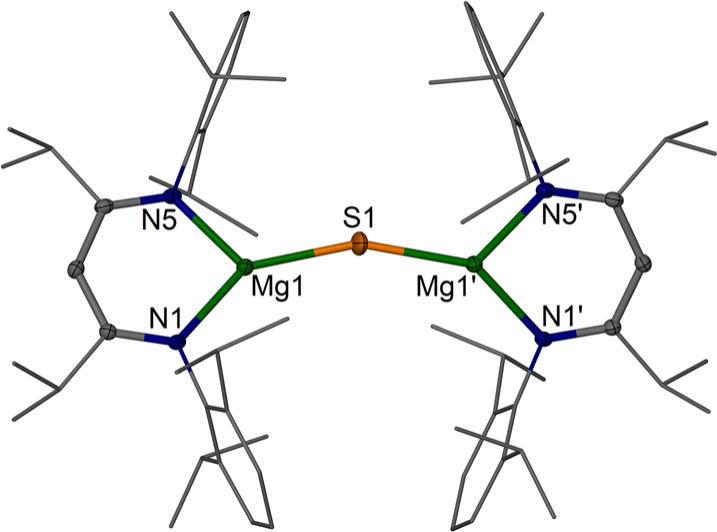
Molecular structure (30% thermal ellipsoids) of [{(^iPrDip^NacNac)Mg}_2_(μ-S)] **4**. Dip and *i*Pr groups are shown as wireframe. Only one of the disordered sulfur positions is shown. Hydrogen atoms are omitted. Selected bond lengths (Å) and angles (deg): Mg–S ca. 2.23–2.24 (mean, see Supporting Information), Mg1–N1 2.0126(16), Mg1–N5 2.0209(16); Mg1–S–Mg1′ ca. 159, N1–Mg1–N5 95.87(6).

**Figure 4:**
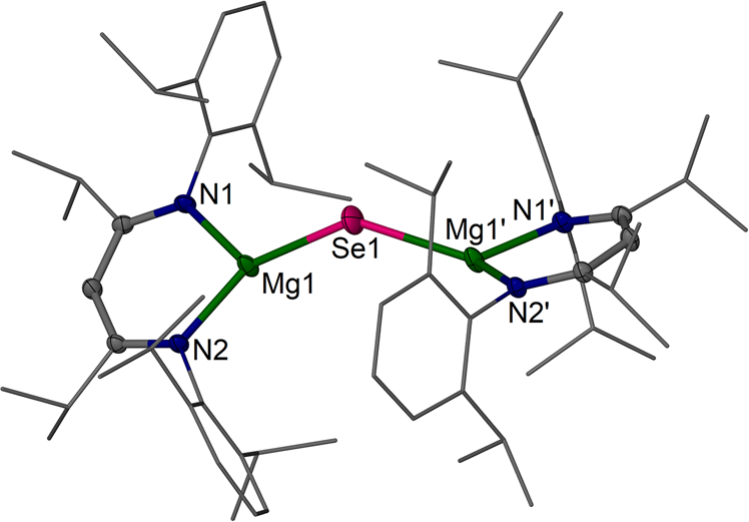
Molecular structure (30% thermal ellipsoids) of [{(^iPrDip^NacNac)Mg}_2_(μ-Se)] **5**. Dip and *i*Pr groups are shown as wireframe. Only one of the main Se positions is shown. Hydrogen atoms are omitted. Selected bond lengths (Å) and angles (deg): Mg1–Se1 2.3497(18), Mg1′–Se1 2.4739(18), Mg1–N2 2.0071(16), Mg1–N1 2.0130(16); Mg1–Se1–Mg1′ 137.89(6).

In contrast, reactions of Ph_3_P=E (E = S, Se) with **1**, followed by NMR spectroscopy in deuterated benzene, proceeded very rapidly at room temperature, giving high *in situ* yields of the low-coordinate chalcogenide complexes [{(^iPrDip^NacNac)Mg}_2_(μ-S)] **4** and [{(^iPrDip^NacNac)Mg}_2_(μ-Se)] **5** (see Figures 3 and 4, respectively) and Ph_3_P (Scheme 1). The isolation of complexes **4** (54%) and **5** (around 30% yield) was predominantly limited by the need for separation from triphenylphosphine and difficulties in the precipitation of the desired product, especially for **5**. NMR spectra for **4** and **5** show, as expected, resonances for highly symmetric complexes in solution. [{(^iPrDip^NacNac)Mg}_2_(μ-Se)] **5** shows a noticeably upfield ^77^Se NMR resonance at −764 ppm. For comparison, Parkin’s tris(pyrazolyl)hydroborato-stabilized magnesium hydroselenido complex [(Tp^*p*-Tol^)Mg(SeH)] displays a chemical shift at −486 ppm.^28^ The much more facile activation of Ph_3_P=E (E = O, S, Se) for E = S, Se compared with E = O can be related to the weaker P=E bonds for E = S (ca. 301 kJ mol^–1^) and Se (ca. 246 kJ mol^–1^) compared with E = O (ca. 546 kJ mol^–1^). The latter is also larger than that of a typical P–C bond (ca. 513 kJ mol^–1^) (cf. the formation of phosphinite **3**).^37,38^

**Scheme 2:**
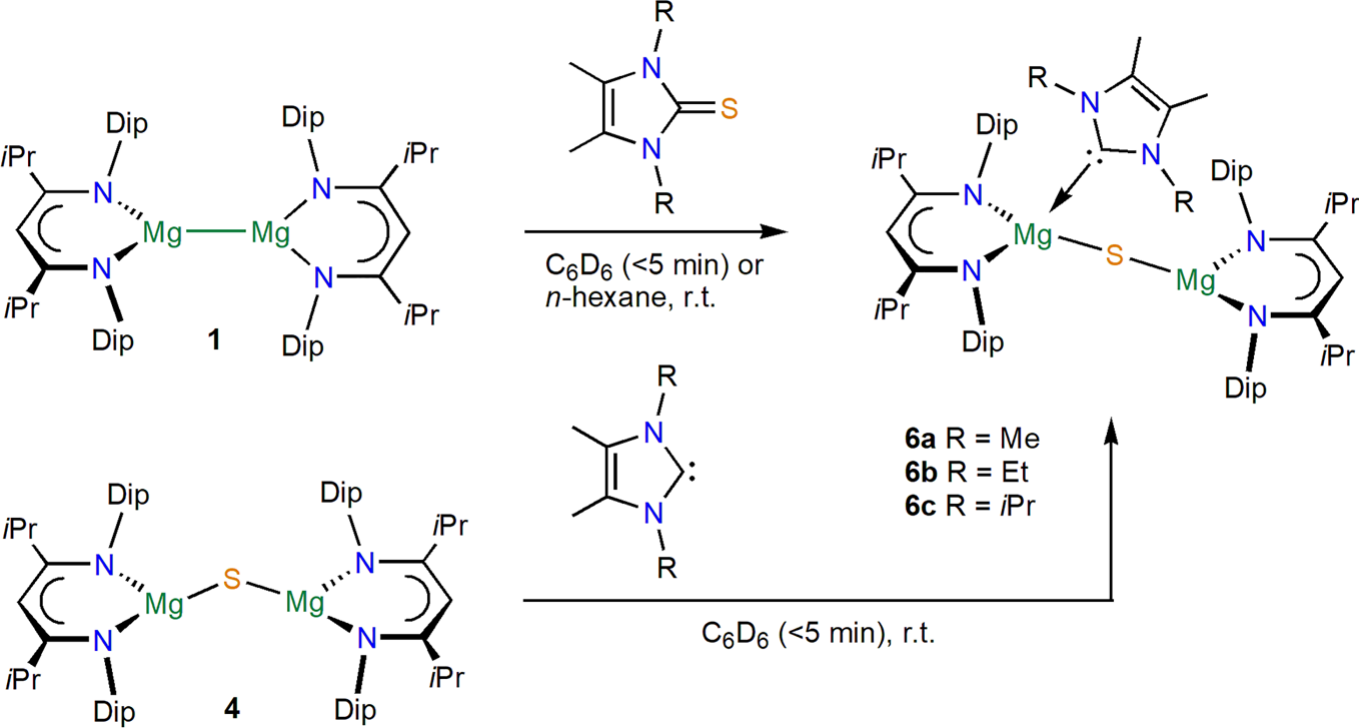
Syntheses of Complexes **6**

**Scheme 3:**
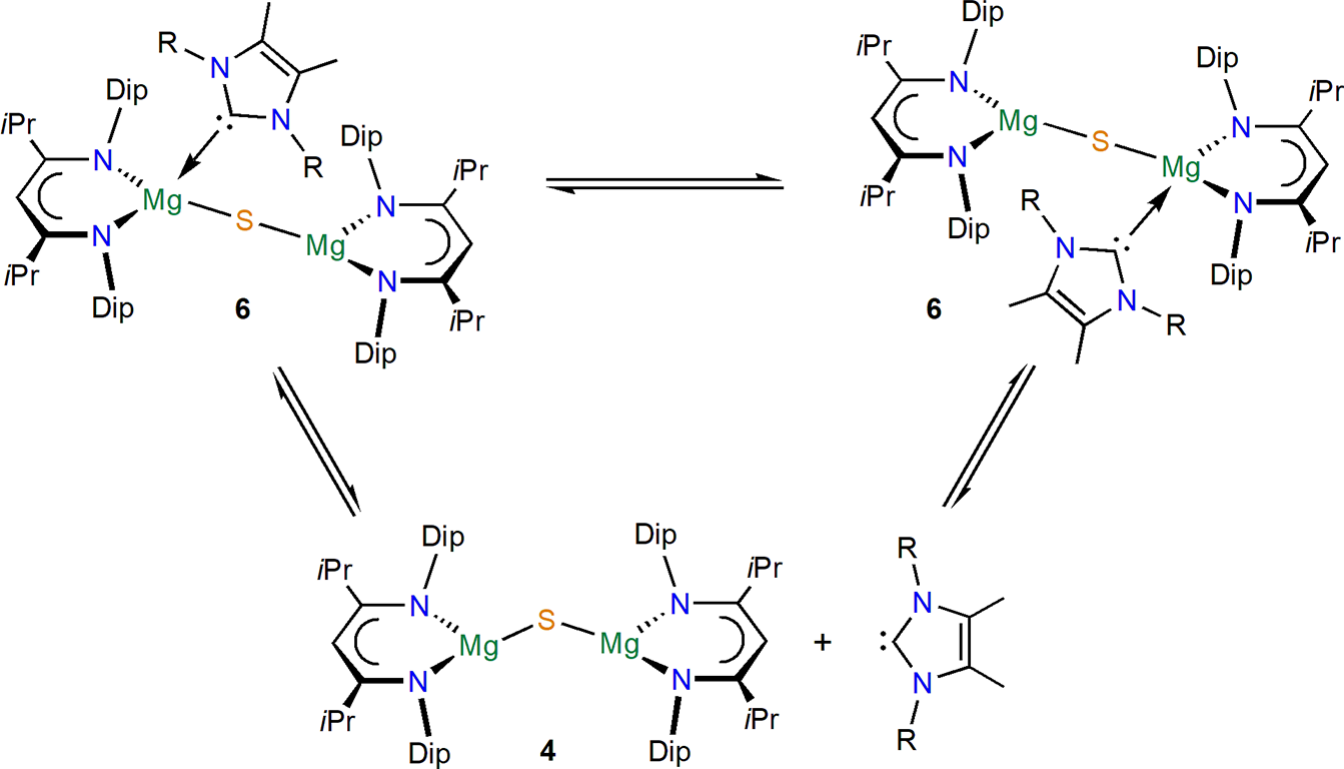
Solution Equilibria of Complexes **6**

Next, reactions between [{(^*i*PrDip^NacNac)Mg}_2_] **1** and substituted imidazole-2-thiones were studied, which also showed rapid conversions at room temperature to donor-substituted sulfide complexes of the type [{(^iPrDip^NacNac)Mg(^R^NHC)}(μ-S){Mg(^iPrDip^NacNac)}] **6** (^R^NHC = (MeCNR)_2_C with R = Me **6a**, Et **6b**, and *i*Pr **6c**) in very high yields, as shown in Scheme 2. Unfortunately, so far these complexes could not be structurally characterized, due to their high solubility and preferential crystallization or precipitation of the uncoordinated sulfide complex **4**, especially for the larger ^R^NHC ligand with R = *i*Pr (**6c**), likely generated in an equilibrium; see Scheme 3. The same compounds (**6**) were shown by NMR spectroscopy to be obtained nearly quantitatively when sulfide complex **4** was treated with one equivalent of the respective *N*-heterocyclic carbene (^R^NHC); see Scheme 2. NMR spectra of **6a** and **6b** both show resonances for two different ligand environments, the latter with slightly broader resonances and ^13^C{^1^H} NMR resonances of 183–184 ppm for the coordinated NHC ligand. The room-temperature ^1^H NMR spectrum for **6c** shows broader resonances and one ligand backbone CH unit. Complexes **6b** and **6c** were studied by variable-temperature NMR spectroscopy (Figures S27 and S32), and for **6b**, resonances above the coalescence temperature of ca. 60 °C show one β-diketiminate ligand environment only. For **6c**, resonances for two ligand sets were observed at low temperature, with coalescence near room temperature. These processes suggest estimated barriers of *ΔG*^⧧^ ≈ 16 kcal mol^–1^ (**6b**) and *ΔG*^⧧^ ≈ 14 kcal mol^–1^ (**6c**) and depend on the steric demand of the ^R^NHC, with the bulkier one decoordinating and rearranging more easily (Scheme 3), likely via donor-free species **4**, which is in line with the repeated observation of crystallization of **4** from such solutions. In a similar manner, the monomeric aluminum(I) complex ^MeDip^NacNacAl: (^MeDip^NacNac = HC(MeNDip)_2_) has been reacted with Ph_3_PS and imidazole-2-thiones to form aluminum sulfide complexes, also forming reduced phosphine or *N*-heterocyclic carbene units, respectively.^39^ For comparison and in contrast to the magnesium chemistry reported herein, the reaction of ^MeDip^NacNacAl: with Ph_3_PO proceeded at room temperature, afforded PPh_3_, and transferred one oxygen to the aluminum center without P–C bond cleavage.^40^

### Reactivity

**Figure 5:**
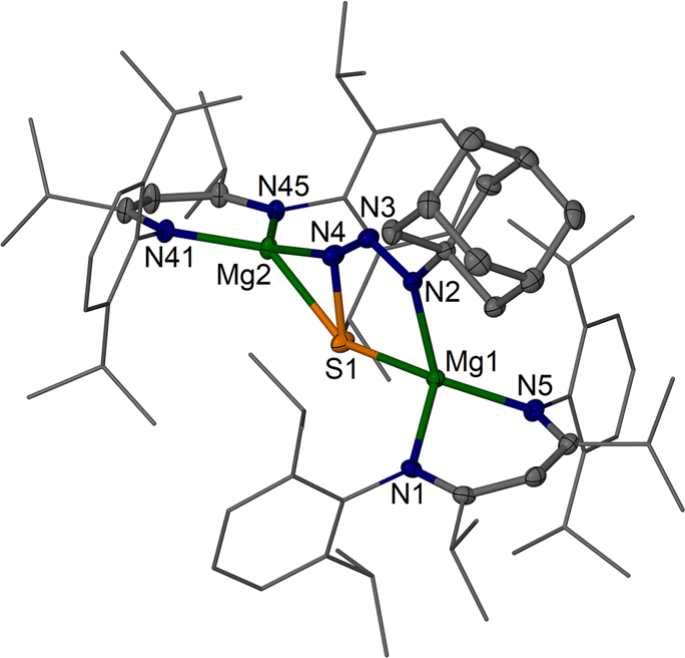
Molecular structure (30% thermal ellipsoids) of [{(^iPrDip^NacNac)Mg}_2_(μ-SN_3_Ad)] **7**. Dip and *i*Pr groups are shown as wireframe. Hydrogen atoms are omitted. Selected bond lengths (Å) and angles (deg): Mg1–S1 2.3926(5), Mg2–S1 2.4343(5), Mg1–N1 2.0443(11), Mg1–N5 2.0523(11), Mg1–N2 2.0944(11), Mg2–N45 2.0111(11), Mg2–N41 2.0098(11), Mg2–N4 2.0123(11), S1–N4 1.8257(12), N2–N3 1.3061(15), N3–N4 1.2726(16); Mg1–S1–Mg2 148.43(2), N4–S1–Mg1 94.98(4), N4–S1–Mg2 54.12(4), N1–Mg1–N5 95.48(5), N2–Mg1–S1 80.86(3), N41–Mg2–N45 97.27(4), N4–Mg2–S1 47.32(3), N4–N3–N2 120.52(11).

**Scheme 4:**
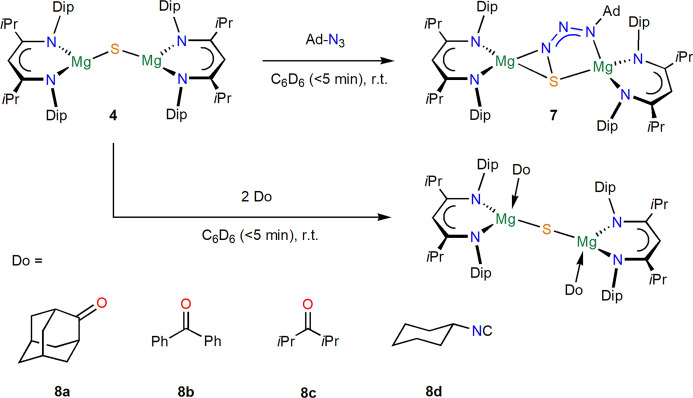
Syntheses of Complexes **7** and **8**

With the low-coordinate sulfide complex [{(^iPrDip^NacNac)Mg}_2_(μ-S)] **4** in hand, an initial assessment of its reactivity was made. The reaction of **4** with 1-adamantylazide (1-azidoadamantane) in deuterated benzene afforded colorless crystals of the S–N bonded complex [{(^iPrDip^NacNac)Mg}_2_(μ-SN_3_Ad)] **7**, see Scheme 4 and Figure 5, in high *in situ* yield (67% isolated). Evidently, the nucleophilic sulfide reacted with an electrophilic site at the organic azide to form a bridging {SN_3_Ad}^2–^ ligand, *vide infra*. To the best of our knowledge, this is the first example of such a ligand fragment, but it bears a resemblance to magnesium complexes of N–N azide coupled {AdN_3_–N_3_Ad}^2–^ fragments^41,42^ and the {SN_2_O}^2–^ ligand formed from sulfide addition to N_2_O in a zinc complex.^33^ At room temperature, complex **7** shows NMR resonances for a more symmetric structure than expected from its molecular structure (i.e., showing one set of ligand resonances suggesting a flexible coordination of the {AdN_3_S}^2–^ ligand between the (^iPrDip^NacNac)Mg^+^ units). At low temperatures, resonances broaden at around −10 °C and Dip-isopropyl group methyl resonances appear to be separated at −45 °C, but the characteristic β-diketiminate backbone methine singlet is not split down to −75 °C, the limit of this experiment (Figure S38), showing the flexible coordination behavior of this system. At high temperatures, complex **7** appears to be quite thermally stable in solution and shows only minimal decomposition after several days at 100 °C in deuterated benzene.

**Figure 6:**
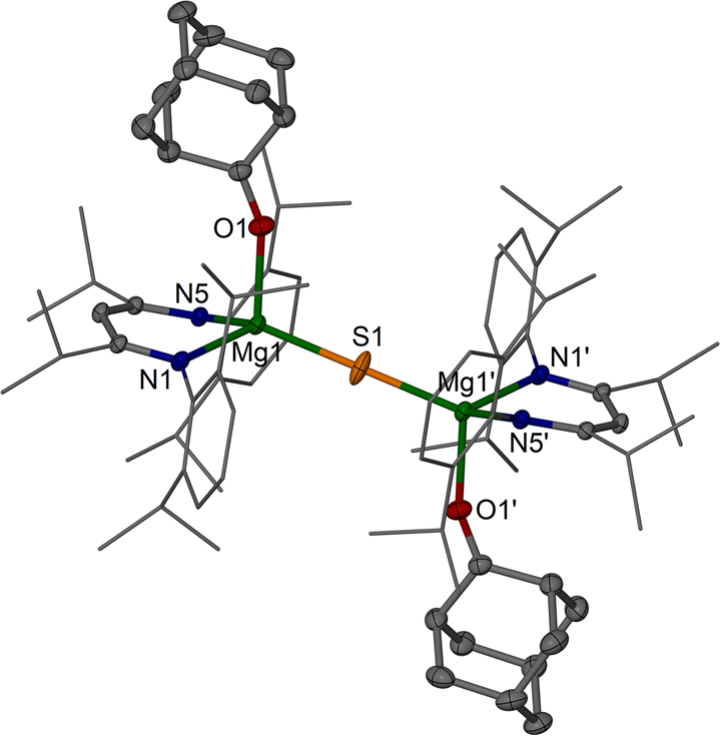
Molecular structure (30% thermal ellipsoids) of [{(^iPrDip^NacNac)Mg(AdO)}_2_(μ-S)] **8a**·C_6_H_6_. Dip and *i*Pr groups are shown as wireframe. Hydrogen atoms, solvent molecule, and minor disordered parts are omitted for clarity. Selected bond lengths (Å) and angles (deg): Mg1–S1 2.2610(5), Mg1′–S1 2.2610(5), Mg1–N1 2.0723(14), Mg1–N5 2.0733(14), Mg1–O1 2.0875(14), O1–C36 1.225(2); Mg1–S1–Mg1′ 180.0, N1–Mg1–N5 94.14(5), N1–Mg1–O1 101.64(6), N5–Mg1–O1 101.49(6), N1–Mg1–S1 122.81(4), N5–Mg1–S1 124.03(4), O1–Mg1–S1 108.91(5).

Reactions of [{(^iPrDip^NacNac)Mg}_2_(μ-S)] **4** with two equivalents of ketones were studied by NMR spectroscopy in deuterated benzene and immediately and quantitatively afforded simple donor adducts [{(^iPrDip^NacNac)Mg(Do)}_2_(μ-S)] **8** in solution, with the donors (Do) 2-adamantanone (OAd) **8a**, benzophenone (OCPh_2_) **8b**, and diisopropylketone (OC*i*Pr_2_) **8c**. The related isonitrile donor adduct [{(^iPrDip^NacNac)Mg(CNCy)}_2_(μ-S)] **8d** was formed on reaction with cyclohexylisonitrile (Scheme 4). Complex [{(^iPrDip^NacNac)Mg(OAd)}_2_(μ-S)] **8a** could in addition be structurally characterized; see Figure 6. For adducts **8**, one set of ^1^H and ^13^C{^1^H} NMR resonances is observed for the β-diketiminate ligand that is significantly shifted from those of low-coordinate **4**. If further uncoordinated **4** is added to these solutions, then still only one set of β-diketiminate ligand resonances is observed, showing the fluxional nature of this interaction, and the resonances shift and sharpen toward those of uncoordinated **4**. No resonances were found for the carbonyl carbon centers in ^13^C{^1^H} NMR spectra of the adducts, possibly due to the fluxional behavior. NMR resonances of adduct [{(^iPrDip^NacNac)Mg(OC*i*Pr_2_)}_2_(μ-S)] **8c** contain broadened peaks. Heating adduct **8c** in deuterated benzene to 80 °C afforded slow conversion to a product mixture according to ^1^H NMR spectroscopy, including a new ^1^H NMR resonance at −0.99 ppm which was tentatively assigned to bridging Mg–SH groups. This was later corroborated by a few colorless crystals that were deposited from solution that were structurally characterized as [{(^iPrDip^NacNac)Mg(μ-SH)}_2_] **9**; see Figure S61. Presumably, the basic nature of the magnesium sulfide can deprotonate the ketone, followed by ligand rearrangements to form [{(^iPrDip^NacNac)Mg(μ-SH)}_2_] **9** as part of a product mixture. Further heating led to the formation of some proligand, ^iPrDip^NacNacH.

### Molecular Structures

[(^iPrDip^NacNac)Mg(OPPh_2_)(OPPh_3_)] **3**, crystallized with a full molecule in the asymmetric unit, shows a distorted tetrahedral Mg center sitting significantly above the chelating diketiminate unit (by approximately 0.99 Å) toward the Ph_2_PO ligand which is located approximately perpendicular with respect to the diketiminate, whereas the larger neutral Ph_3_PO donor is approximately in plane with the diketiminate unit. The Mg–O bond distance to the anionic diphenylphosphinite is only slightly shorter than that to the neutral triphenylphosphinoxide (Mg1–O2 1.9000(16) versus Mg1–O1 1.9243(14)), and the P–O bond in the P^V^ ligand (P1–O1 1.4972(13)) is, as expected, shorter than that in the phosphinite (P2–O2 1.5459(17)). These features compare well to those of other alkaline earth metal complexes with diphenylphosphinite ligands.^43−48^

The molecular structure of [{(^iPrDip^NacNac)Mg}_2_(μ-S)] **4** was determined multiple times, and the best two data sets are included here. In all cases, **4** was obtained with half a molecule of the complex in the asymmetric unit and contains three-coordinate Mg centers. The sulfur positions were found to be disordered on or very close to special positions in the asymmetric unit. As such, the multiple individual bond lengths are not given and a mean value of Mg–S 2.23–2.24 Å was obtained. The Mg–S–Mg angles for this model show some variation, with an average around 159°, and are likely flexible and easy to distort. For context, the Mg–S distances in rock salt MgS are ca. 2.57 Å and in zinc blende MgS are ca. 2.42 Å, showing the expected significant effect of the coordination number on bond distances.^49^ An Mg–S distance of 2.44 Å can be inferred from single-bond covalent radii^50^ and show the significant shortening in **4**, but not as low as that determined for diatomic MgS of ca. 2.143 Å.^51^ The Mg–S bond lengths in **4** are significantly shorter than those observed in Ren’s disulfide-bridged NacNac magnesium complex **A** (2.4518(10) Å and 2.5149(10) Å) and those found for related terminal NacNac magnesium thiolates (e.g., in [(^Dip^NacNac)Mg(THF)(SPh)]^26^ (Mg1–S1 2.3839(9) Å)). In addition, a few related low-coordinate β-diketiminate metal(II) sulfide complexes of general formula [{(NacNac)M}_2_(μ-S)] exist with M = Fe,^52^ Ni,^53^ and Sn.^54^

MgSe complex **5** is isomorphous to **4**, and again the Se atom is disordered on or very close to special positions. There is, however, one main position in the asymmetric unit which shows two different Mg–Se distances (Se1–Mg1 2.3497(18), Se1–Mg1′ 2.4739(18) Å) among shorter contacts and a relatively acute Mg1–Se1–Mg1 angle of 137.89(6)°, alongside more obtuse angles, and likely again hints that the Se position in this ionic arrangement is easy to distort and preferably bent. Parkin’s magnesium selenide complex [{(^*p*-Tol^Tp)Mg}_2_Se] **B**(28) displays comparable Mg–Se bond lengths of 2.404(3) Å and 2.408(3) Å in a four-coordinate Mg environment and a linear Mg–Se–Mg moiety. In solid MgSe, the Mg–Se distances are ca. 2.70 Å (rock salt) and ca. 2.54 Å (zinc blende),^49^ and single bond radii provide 2.55 Å.^50^

Complex [{(^iPrDip^NacNac)Mg}_2_(μ-SN_3_Ad)] **7**, crystallized with a full molecule in the asymmetric unit. The central {SN_3_Ad}^2–^ ligand bridges two Mg centers in a μ-κ^2^*S*,*N3*:κ^2^*S*,*N1* fashion. The N–N bond lengths in **7** (N2–N3 1.3061(15) Å, N3–N4 1.2726(16) Å) are indicative of significant delocalization across the N_3_ fragment and are similar to those in the two N_3_ fragments in the N_6_-chain complexes of [{(^MeDip^NacNac)Mg(μ-N_3_Ad)}_2_].^41,42^ The S–N bond (1.8257(12) Å) is noticeably longer than would typically be expected for a S–N single bond (ca. 1.74),^50^ likely due to electrostatic repulsion in the dianionic unit. To the best of our knowledge, this is the first structurally characterized {SN_3_R}^2–^ ligand that has some resemblance to the {SN_2_O}^2–^ coordinated to a zinc center.^33^

The donor adduct [{(^iPrDip^NacNac)Mg(AdO)}_2_(μ-S)] **8a** with four-coordinate Mg centers shows a linear Mg–S–Mg fragment (cf. the linear arrangement in **B**) with Mg–S bond lengths of 2.2610(5) Å that are only slightly elongated compared to the mean value found in uncoordinated [{(^iPrDip^NacNac)Mg}_2_(μ-S)] **4**. The hydrosulfide (bisulfide) complex [{(^iPrDip^NacNac)Mg(μ-SH)}_2_] **9** (Figure S61) shows the expected dimeric structure with a Mg–S distance of 2.51 Å (mean) and a Mg–S–Mg angle of 89.52(3)° and can be compared to the shorter Mg–S distance of 2.4424(6) Å in [{(^MeDip^NacNac)Mg(μ-S*n*Bu)}_2_].^25^

### Computational Studies

A DFT study at the M06L/def2-TZVP level of theory with D3 dispersion addition followed by single-point calculations at the M06-D3/def2-TZVP level of theory (M06-D3/def2-TZVP//M06-L-D3/def2-TZVP) was conducted for the compound series with the full ligand model [{(^iPrDip^NacNac)Mg}_2_(μ-E)] E = O (**2**), S (**4**), Se (**5**) and the small ligand model system [{(^MeMe^NacNac)Mg}_2_(μ-E)] (^MeMe^NacNac = HC(MeNMe)_2_), E = O, S, Se, see Table 1 for selected data). The optimization of both the full ligand sphere and a cut-back model was conducted to gain some insight into the influence of the ligand bulk for these species. The DFT optimized full ligand model species [{(^iPrDip^NacNac)Mg}_2_(μ-E)] reproduced the overall structures found by X-ray diffraction well, but the sterically smaller models [{(^MeMe^NacNac)Mg}_2_(μ-E)] reproduce only the linear Mg–O–Mg geometry with coplanar metal–ligand arrangements well but show significantly more bent Mg–E–Mg units for E = S and Se, resulting in sub-90° bond angles. These trends show the importance of the influence of the sterically demanding ligands for the structures of the compounds for E = S and Se and may provide a reason for the significant disorder of the S and Se positions in the molecular structures of **4** and **5**. The sub-90° bond angles in [{(^MeMe^NacNac)Mg}_2_(μ-S)] for E = S and Se appear to be in part due to influences from dispersion forces.^55,56^ Removing the dispersion addition for [{(^MeMe^NacNac)Mg}_2_(μ-S)] leads to a more relaxed Mg–E–Mg angle of 106.4° (E = S) but a virtually unchanged angle for E = Se (82.6°), suggesting that these systems are easy to distort (Figure S64).

**Table 1:** Selected Metrical Data and Calculated Charges from NPA and QTAIM

	Mg–E [Å]	Mg–E–Mg [deg]	charge on Mg NPA (QTAIM)	charge on E NPA (QTAIM)
[{(^iPrDip^NacNac)Mg}_2_(μ-O)] **2**	1.805	179.9	+1.88 (+1.73)	–1.86 (−1.64)
[{(^MeMe^NacNac)Mg}_2_(μ-O)]	1.799	179.9	+1.81	–1.84
[{(^iPrDip^NacNac)Mg}_2_(μ-S)] **4**	2.243	139.8	+1.76 (+1.69)	–1.69 (−1.54)
[{(^MeMe^NacNac)Mg}_2_(μ-S)]	2.275	87.58	+1.69	–1.61
[{(^iPrDip^NacNac)Mg}_2_(μ-Se)] **5**	2.377	131.0	+1.73 (+1.68)	–1.65 (−1.49)
[{(^MeMe^NacNac)Mg}_2_(μ-Se)]	2.413	82.51	+1.66	–1.56

**Figure 7:**
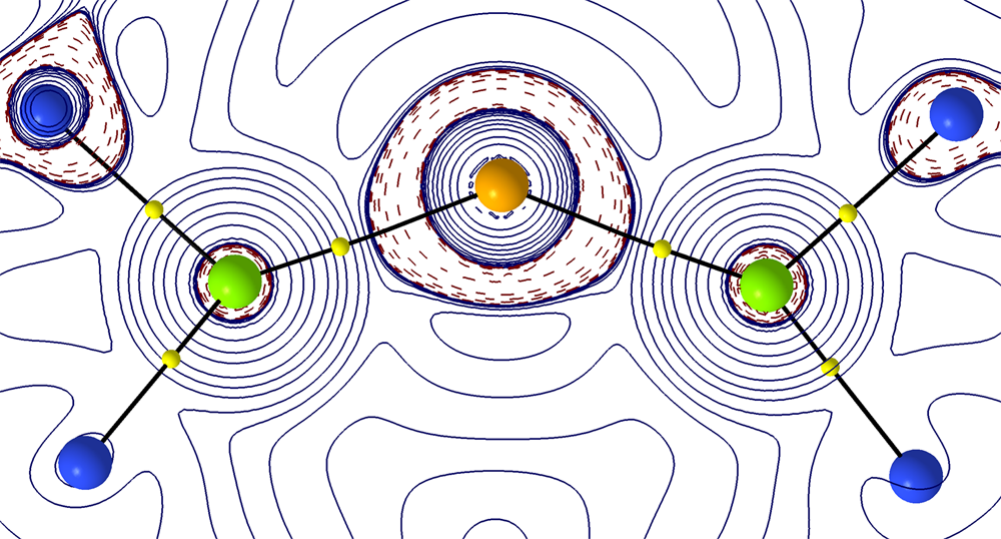
QTAIM contour plots of the Laplacian of electron density (solid lines positive, dashed lines negative) for [{(^iPrDip^NacNac)Mg}_2_(μ-S)] **4** through the Mg_2_S plane showing only the core atoms (S orange, Mg green, and N blue), selected bond paths (black), and bond critical points (yellow). Selected values for the electron density, ρ (black), Laplacian, ∇^2^ρ (blue), and bond ellipticity, ε (green), are given for key bond critical points in Table S2.

Inspecting the calculated charges of the Mg and E atoms from natural population analysis (NPA) and the quantum theory of atoms in molecules (QTAIM) analysis in the series shows the most charge separation on Mg with the most electronegative O and less charge separation descending the chalcogen group to S and Se. The negative charge accumulation and slight polarization by the Mg centers at the chalcogen atom (E) can be visualized in the contour plot of the Laplacian of the electron density for [{(^iPrDip^NacNac)Mg}_2_(μ-S)] **4** (Figure 7) and in related images for E = O (Figure S65) and Se (Figure S66). High-lying occupied orbitals for [{(^iPrDip^NacNac)Mg}_2_(μ-E)] show *p*_*x*_ and *p*_*y*_ orbitals of the chalcogens approximately perpendicular to the Mg–E–Mg bond (for O, HOMO–3 and HOMO–4; for S and Se, HOMO–1 and HOMO–2) and one significantly stabilized *p*_*z*_ orbital (for O, HOMO–12; for S, HOMO–12; for Se, HOMO–11) approximately along the Mg–E–Mg bond which is around 1.4 eV (ca. 32 kcal/mol, 135 kJ/mol) lower in energy compared to the respective *p*_*x*_ and *p*_*y*_ orbitals (see Figures S62 and S63). Other occupied orbitals show common β-diketiminate ligand-based features. All of these findings support that these complexes possess highly ionic Mg–E–Mg units that are easy to distort for E = S and Se.

## Conclusions

Reactions of the magnesium(I) complex [{(^iPrDip^NacNac)Mg}_2_] **1** with Ph_3_P=E (E = O, S, Se) required forcing conditions for E = O and afforded the phosphinate complex [(^iPrDip^NacNac)Mg(OPPh_3_)(OPPh_2_)] **3** highlighting P–C rather than P–O bond cleavage. For E = S and Se, facile reactions afforded the low-coordinate chalcogenide complexes [{(^iPrDip^NacNac)Mg}_2_(μ-S)] **4** and [{(^iPrDip^NacNac)Mg}_2_(μ-Se)] **5**, respectively, which show low-coordinate Mg–E–Mg units with short Mg–E bonds. Similarly, ^R^NHC=S species were rapidly reduced by **1** to magnesium sulfide complexes [{(^iPrDip^NacNac)Mg(^R^NHC)}(μ-S){Mg(^iPrDip^NacNac)}] **6**. Sulfide complex **4** readily reacted with 1-adamantylazide (AdN_3_) to afford [{(^iPrDip^NacNac)Mg}_2_(μ-SN_3_Ad)] **7** with a dianionic ligand containing a new S–N bond. Donor adducts of magnesium sulfide complexes were also obtained, for example, the structurally characterized example [(^iPrDip^NacNac)Mg(OAd)}_2_(μ-S)] **8a** (OAd = 2-adamantanone) that features a linear Mg–S–Mg unit. DFT computational studies highlight the ionic nature of the Mg–E–Mg units (E = O, S, Se) in [{(NacNac)Mg}_2_(μ-E)] complexes, and structural and computational studies show the linear arrangement for E = O and the range of severely bent to linear geometries for E = S and Se.

## Supporting Information Available

Experimental section, NMR spectroscopy, X-ray crystallography, and DFT computational studies (PDF)
