# Supplementary material for: Low-Coordinate Magnesium Sulfide and Selenide Complexes
Source: Inorg Chem. 2023 Sep 25;62(40):16443–50. doi: 10.1021/acs.inorgchem.3c02132 (PMC10565804; doi:10.1021/acs.inorgchem.3c02132)
Supplement: Supplementary file 1 — ic3c02132_si_001.pdf [file ic3c02132_si_001.pdf]

Electronic supplementary information

Low Coordinate Magnesium Sulfide and Selenide Complexes

Stuart Burnett[a], Rochelle Ferns[a], David B. Cordes[a], Alexandra M. Z. Slawin[a], Tanja van Mourik[a], and Andreas Stasch[a]*

[a]*EaStCHEM School of Chemistry, University of St Andrews, North Haugh, St Andrews, KY16 9ST, United Kingdom*

*Email: as411@st-andrews.ac.uk

Table of Contents

1	Experimental Section	2
2	NMR Spectroscopy	9
3	X-ray Crystallography	47
4	DFT Computational Studies	56
5	References	74

1 Experimental Section

1.1 General considerations

All manipulations were carried out using standard Schlenk and glove box techniques under a dry argon or dinitrogen atmosphere. Benzene, toluene, and *n*-hexane were either dried and distilled under inert gas over $LiAlH_4$, sodium or potassium, or taken from an MBraun solvent purification system and degassed prior to use. ^{1}H, $^{13}C\{^{1}H\}$, and $^{31}P\{^{1}H\}$ NMR spectra were recorded on a Bruker AV 300, Bruker AVII 400 or Bruker AV III 500 spectrometer in deuterated benzene or toluene and were referenced to the residual ^{1}H or $^{13}C\{^{1}H\}$ resonances of the solvent used, or external aqueous H_3PO_4 solutions, respectively. Chemical shifts are given in ppm. Abbreviations: s = singlet, d = doublet, t = triplet, q = quartet, sept = septet, br = broad, m = multiplet. Elemental analyses were performed by the Elemental Analysis Service at London Metropolitan University; for few, likely due to the highly air- and moisture-sensitive nature of the compounds, accurate and reliable results for isolated species could not be obtained. Other species were only generated in solution and were not isolated in pure form. 1,3,4,5-trimethyl-imidazole-2-thione (^{Me}NHC=S), 1,3-diethyl-4,5-dimethyl-imidazole-2-thione (^{Et}NHC=S), 1,3-diisopropyl-4,5-dimethyl-imidazole-2-thione (^{iPr}NHC=S), ^{Me}NHC, ^{Et}NHC, and ^{iPr}NHC were synthesised as described in the literature.[1] [{(^{iPrDip}NacNac)Mg}$_2$] **1**,[2] and Ph_3PSe[3] were synthesised as described before. All other reagents were used as received.

1.2 Syntheses

[(^{iPrDip}NacNac)Mg($OPPh_3$)($OPPh_2$)] 3

A benzene-d_6 solution (0.5 mL) of [{(^{iPrDip}NacNac)Mg}$_2$] (70 mg, 0.070 mmol) **1** and triphenylphosphine oxide (78.1 mg, 0.28 mmol, 4.0 equivs.) was heated in a J. Young's NMR tube to 100 °C for two days, during which time a gradual colour change from yellow to red was observed. All volatiles were subsequently removed *in vacuo* and the residue extracted with *n*-hexane (1 mL). Leaving to stand for 16 hours at 20 °C afforded **3** as a red crystalline solid. This red colour likely arises from small traces of impurities as additional crops of **3** were much paler red, while some crops were completely colourless. Yield = 59.8 mg (44 %). ^{1}H NMR (400.1 MHz, benzene-d_6, 294 K) δ = 0.86-0.91 (m, 12H, Ar-*o*-CH(C*H*$_3$)$_2$), 0.98 (d, J_{HH} = 6.7 Hz, 6H, Ar-*o*-CH(C*H*$_3$)$_2$), 1.16 (d, J_{HH} = 6.7 Hz, 6H, NCCH(C*H*$_3$)$_2$), 1.30 (d, J_{HH} = 6.9 Hz, 6H, Ar-*o*-CH(C*H*$_3$)$_2$), 1.35 (d, J_{HH} = 6.7 Hz, 6H, NCCH(C*H*$_3$)$_2$), 2.68 (sept, J_{HH} = 6.7 Hz, 2H, NCC*H*(CH$_3$)$_2$), 3.35 (sept, J_{HH} = 6.7 Hz, 2H, Ar-*o*-C*H*(CH$_3$)$_2$), 3.87 (sept, J_{HH} = 6.7 Hz, 2H, Ar-*o*-C*H*(CH$_3$)$_2$), 5.26 (s, 1H, NC(CH(CH$_3$)$_2$)C*H*), 6.91-7.14 (m, 23H, Ar-*H*), 7.27 (t, J_{HH} = 7.5 Hz, 4H, Ar-*H*), 8.08 (br t, 4H, Ar-*H*). $^{13}C\{^{1}H\}$ NMR (100.5 MHz, benzene-d_6, 294 K): δ = 14.4 (Ar-*o*-CH(*C*H$_3$)$_2$), 23.1 (NCCH(*C*H$_3$)$_2$), 23.3 (NCCH(*C*H$_3$)$_2$), 23.7 (Ar-*o*-CH(*C*H$_3$)$_2$), 24.3 (Ar-*o*-CH(*C*H$_3$)$_2$), 24.6 (Ar-*o*-CH(*C*H$_3$)$_2$), 26.8 (Ar-*o*-*C*H(CH$_3$)$_2$), 27.4

(Ar-*o*-*C*H(CH_3)$_2$), 32.6 (NC*C*H(CH_3)$_2$), 85.8 (NC(CH(CH_3)$_2$)*C*H), 123.8 (Ar-*C*), 124.4 (Ar-*C*), 124.6 (Ar-*C*), 126.9 (Ar-*C*), 129.1 (Ar-*C*), 129.2 (Ar-*C*), 129.5 (Ar-*C*), 130.0 (Ar-*C*), 130.2 (Ar-*C*), 132.6 (Ar-*C*), 132.7 (Ar-*C*), 143.0 (Ar-*C*), 145.3 (Ar-*C*), 145.7 (Ar-*C*), 154.9 (Ar-*C*), 155.2 (Ar-*C*), 178.8 (N*C*CH(CH_3)$_2$). $^{31}P\{^1H\}$ (161.97 MHz, benzene-d_6, 294 K) $\delta = 36.8$ (Ph_3PO), 85.8 (Ph_2PO^-).

[{(^{iPrDip}NacNac)Mg}$_2$(μ-S)] 4

[{(^{iPrDip}NacNac)Mg}$_2$] (200 mg, 0.200 mmol) **1** and triphenylphosphine sulfide (59.1 mg, 0.200 mmol) were dissolved in toluene (25 mL) at 20 °C and stirred for 16 hours, during which time a colour change from yellow to colourless and formation of a white precipitate was observed. The precipitate was allowed to settle, filtered and dried under vacuum yielding **4** as a white powder. Concentrating the filtrate to *ca.* 5 mL and storing at -40 °C for two days afforded a second crop of **4**. Crystals suitable for X-ray crystallographic analysis were grown from a concentrated toluene solution. Yield = 111 mg (54 %). ^1H NMR (400.1 MHz, benzene-d_6, 294 K) $\delta = 1.00$ (d, $J_{HH} = 6.6$ Hz, 24H, NCCH(C*H*$_3$)$_2$), 1.16 (d, $J_{HH} = 6.6$ Hz, 24H, Ar-*o*-CH(C*H*$_3$)$_2$), 1.21 (d, $J_{HH} = 6.9$ Hz, 24H, Ar-*o*-CH(C*H*$_3$)$_2$), 2.58 (sept, $J_{HH} = 6.8$ Hz, 4H, NC(C*H*(CH_3)$_2$), 3.08 (sept, $J_{HH} = 6.9$ Hz, 8H, Ar-*o*-C*H*(CH_3)$_2$), 4.96 (s, 2H, NC(CH(CH_3)$_2$)C*H*), 7.07-7.13 (m, 12H, Ar-*H*). $^{13}C\{^1H\}$ NMR (100.5 MHz, benzene-d_6, 294 K): $\delta = 22.8$ (NC(CH(*C*H$_3$)$_2$)), 23.5 (Ar-*o*-CH(*C*H$_3$)$_2$), 26.1 (Ar-*o*-CH(*C*H$_3$)$_2$), 28.1 (Ar-*o*-*C*H(CH_3)$_2$), 31.8 (NC(CH(*C*H$_3$)$_2$)), 85.1 (NC(CH(CH_3)$_2$)*C*H), 123.8 (Ar-*C*), 125.6 (Ar-*C*), 142.5 (Ar-*C*), 142.6 (Ar-*C*), 179.3 ((N*C*(CH(CH_3)$_2$)CH). Elemental analysis: calculated for $C_{66}H_{98}N_4Mg_2S$: C 77.10; H 9.61; N 5.45%; found: C 76.91; H 9.31; N 5.25%.

[{(^{iPrDip}NacNac)Mg}$_2$(μ-Se)] 5

[{(^{iPrDip}NacNac)Mg}$_2$] (100 mg, 0.200 mmol) **1** and triphenylphosphine selenide (68.6 mg, 0.200 mmol) were dissolved in toluene (25 mL) at 20 °C and stirred for 16 hours, during which time a colour change from yellow to colourless was observed. All volatiles were subsequently removed *in vacuo* and the residue extracted with n-hexane (30 mL). Filtration, concentration of the supernatant solution to *ca.* 10 mL and storing at -40 °C for one day afforded a second crop of **5**. Crystals suitable for X-ray crystallographic analysis were grown from a concentrated benzene-d_6 solution. Yield = 31.5 mg (29%). ^1H NMR (400.1 MHz, benzene-d_6, 294 K) $\delta = 1.00$ (d, $J_{HH} = 6.7$ Hz, 24H, NCCH(C*H*$_3$)$_2$), 1.20 (d, $J_{HH} = 6.7$ Hz, 48H, Ar-*o*-CH(C*H*$_3$)$_2$), 2.58 (sept, $J_{HH} = 6.7$ Hz, 4H, NC(C*H*(CH_3)$_2$), 3.09 (sept, $J_{HH} = 6.7$ Hz, 8H, Ar-*o*-C*H*(CH_3)$_2$), 4.97 (s, 2H, NC(CH(CH_3)$_2$)C*H*), 7.08-7.14 (m, 12H, Ar-*H*). $^{13}C\{^1H\}$ NMR (100.5 MHz, benzene-d_6, 294 K): $\delta = 22.9$ (NC(CH(*C*H$_3$)$_2$)), 23.7 (Ar-*o*-CH(*C*H$_3$)$_2$), 26.4 (Ar-*o*-CH(*C*H$_3$)$_2$), 28.1 (Ar-*o*-*C*H(CH_3)$_2$), 31.9 (NC(*C*H(CH_3)$_2$)), 85.3 (NC(CH(CH_3)$_2$)*C*H), 123.9 (Ar-*C*), 125.7 (Ar-*C*), 142.4 (Ar-*C*), 142.7 (Ar-

C), 179.5 (N*C*(CH(CH$_3$)$_2$)CH). ^{77}Se{^{1}H} NMR (95.4 MHz, benzene-*d*$_6$, 294 K): δ = -764.3 (Mg-Se-Mg).

[{(^{iPrDip}NacNac)Mg(^{Me}NHC)}(μ-S){Mg(^{iPrDip}NacNac)}] 6a

Method 1: [{(^{iPrDip}NacNac)Mg}$_2$(μ-S)] **1** (10 mg, 9.7 μmol) and ^{Me}NHC ({MeCNMe}$_2$C:, 1.21 mg, 9.7 μmol) were dissolved in benzene-*d*$_6$ (0.5 mL) at 20 °C, yielding a colourless solution. Analysis by ^{1}H NMR spectroscopy showed immediate consumption of starting materials and formation of **6a**. *Method 2:* [{(^{iPrDip}NacNac)Mg}$_2$] **1** (100 mg, 0.100 mmol) and 1,3,4,5-trimethyl-imidazole-2-thione (^{Me}NHC=S, 15.7 mg, 0.100 mmol) were dissolved in *n*-hexane (30 mL) at 20 °C and stirred for 16 hours, during which time a colour change from yellow to colourless was observed. Due to high product solubility and tendency for precipitation of uncoordinated [{(^{iPrDip}NacNac)Mg}$_2$(μ-S)] **4**, no solid/pure **6a** could be isolated. Due to the overlapping nature of some sets of resonances, some assignment is tentative: ^{1}H NMR (400.1 MHz, benzene-*d*$_6$, 294 K) δ = 0.59 (d, J_{HH} = 6.7 Hz, 6H, Ar-*o*-CH(C*H*$_3$)$_2$), 1.01 (d, J_{HH} = 6.7 Hz, 12H, Ar-*o*-CH(C*H*$_3$)$_2$ or NC(CH(C*H*$_3$)$_2$)), 1.04 (d, J_{HH} = 6.7 Hz, 6H, Ar-*o*-CH(C*H*$_3$)$_2$), 1.12 (d, J_{HH} = 6.7 Hz, 6H, Ar-*o*-CH(C*H*$_3$)$_2$), 1.16 (d, J_{HH} = 6.7 Hz, 6H, Ar-*o*-CH(C*H*$_3$)$_2$), 1.17 (d, J_{HH} = 6.6 Hz, 12H, Ar-*o*-CH(C*H*$_3$)$_2$ or NC(CH(C*H*$_3$)$_2$)), 1.23 (d, J_{HH} = 6.8 Hz, 12H, Ar-*o*-CH(C*H*$_3$)$_2$ or NC(CH(C*H*$_3$)$_2$)), 1.35 (d, J_{HH} = 6.8 Hz, 6H, Ar-*o*-CH(C*H*$_3$)$_2$), 1.41 (d, J_{HH} = 6.8 Hz, 6H, Ar-*o*-CH(C*H*$_3$)$_2$), 1.58 (s, 3H, NCC*H*$_3$ (NHC)), 1.63 (s, 3H, NCC*H*$_3$ (NHC)), 2.57 (2 overlapping sept, J_{HH} = 6.6 Hz, 4H, NC(C*H*(CH$_3$)$_2$), 2.90 (sept, J_{HH} = 6.6 Hz, 2H, Ar-*o*-C*H*(CH$_3$)$_2$), 3.22 (2 overlapping sept, J_{HH} = 6.6 Hz, 4H, Ar-*o*-C*H*(CH$_3$)$_2$), 3.32 (sept, J_{HH} = 6.6 Hz, 2H, Ar-*o*-C*H*(CH$_3$)$_2$), 3.40 (s, 3H, NC*H*$_3$ (NHC)), 3.55 (s, 3H, NC*H*$_3$ (NHC)), 4.87 (s, 1H, NC(CH(CH$_3$)$_2$)C*H*), 5.01 (s, 1H, NC(CH(CH$_3$)$_2$)C*H*), 7.04-7.13 (m, 8H, Ar-*H*), 7.20-7.23 (m, 4H, Ar-*H*). ^{13}C{^{1}H} NMR (125.7 MHz, benzene-*d*$_6$, 294 K): δ = 8.4 (NC*C*H$_3$ (NHC)), 8.5 (NC*C*H$_3$ (NHC)), 22.9, 23.05, 23.1, 23.7, 23.8, 24.7, 25.0, 25.6, 26.2, 26.4, 27.5, 27.8, 28.0, 28.1 (NC(CH(*C*H$_3$)$_2$), Ar-*o*-CH(*C*H$_3$)$_2$, Ar-*o*-*C*H(CH$_3$)$_2$), 31.5 (NC(*C*H(CH$_3$)$_2$), 35.5 (N*C*H$_3$ (NHC)), 35.8 (N*C*H$_3$ (NHC)), 85.1 (NC(CH(CH$_3$)$_2$)*CH*), 85.4 (NC(CH(CH$_3$)$_2$)*CH*), 122.8 (Ar-*C*), 123.8 (Ar-*C*), 123.9 (Ar-*C*), 124.37 (Ar-*C*), 124.40 (Ar-*C*), 124.5 (Ar-*C*), 125.1 (Ar-*C*), 142.2 (Ar-*C*), 142.7 (Ar-*C*), 143.0 (Ar-*C*), 144.3 (Ar-*C*), 145.4 (Ar-*C*), 176.6 (N*C*(CH(CH$_3$)$_2$)CH), 179.1 (N*C*(CH(CH$_3$)$_2$)CH), 184.1 ((RN)$_2$*C*: (NHC)).

[{(^{iPrDip}NacNac)Mg(^{Et}NHC)}(μ-S){Mg(^{iPrDip}NacNac)}] 6b

Method 1: [{(^{iPrDip}NacNac)Mg}$_2$(μ-S)] **1** (10 mg, 9.7 μmol) and ^{Et}NHC ({MeCNEt}$_2$C:, 1.5 mg, 9.7 μmol) were dissolved in benzene-*d*$_6$ (0.5 mL) at 20 °C, yielding a colourless solution. Analysis by ^{1}H NMR spectroscopy showed immediate consumption of starting materials and formation of **6b**.

Method 2: [{(^{iPrDip}NacNac)Mg}$_2$] **1** (100 mg, 0.100 mmol) and 1,3-diethyl-4,5-dimethyl-imidazole-2-thione (^{Et}NHC=S, 18.5 mg, 0.100 mmol) were dissolved in n-hexane (30 mL) at 20 °C and stirred for 16 hours, during which time a colour change from yellow to colourless was observed. Due to high product solubility and tendency for precipitation of uncoordinated [{(^{iPrDip}NacNac)Mg}$_2$(μ-S)] **4**, no solid/pure **6b** could be isolated. Due to the overlapping nature of some sets of resonances, some assignment is tentative: ^{1}H NMR (400.1 MHz, benzene-d_6, 294 K) δ = 0.61-0.63 (br m, 9H, Ar-*o*-CH(C*H*$_3$)$_2$ and NCH$_2$C*H*$_3$ (NHC)), 0.99 (br d, 12H, NC(CH(C*H*$_3$)$_2$)), 1.03-1.05 (br m, 6H, NC(CH(C*H*$_3$)$_2$)), 1.11 (br d, 6H, NC(CH(C*H*$_3$)$_2$)), 1.15-1.27 (m, 33H, Ar-*o*-CH(C*H*$_3$)$_2$ and NCH$_2$C*H*$_3$ (NHC)), 1.31-1.36 (m, 12H, Ar-*o*-CH(C*H*$_3$)$_2$), 1.66 (br s, 6H, NC(C*H*$_3$)$_2$ (NHC)), 2.49-2.61 (m/overlapping sept, 4H, NC(C*H*(CH$_3$)$_2$)), 2.97 (br sept, 2H, Ar-*o*-C*H*(CH$_3$)$_2$), 3.15 (br sept, 4H, Ar-*o*-C*H*(CH$_3$)$_2$), 3.34 (br sept, 2H, Ar-*o*-C*H*(CH$_3$)$_2$), 4.11 (br q, 2H, NC*H*$_2$CH$_3$ (NHC)), 4.52 (br q, 2H, NC*H*$_2$CH$_3$ (NHC)), 4.84 (br s, 1H, NC(CH(CH$_3$)$_2$)C*H*), 5.01 (br s, 1H, NC(CH(CH$_3$)$_2$)C*H*), 7.01-7.19 (m, 12H, Ar-*H*). ^{13}C{^{1}H} NMR (100.5 MHz, benzene-d_6, 294 K): δ = 8.6 (NC*C*H$_3$ (NHC)), 9.0 (NC*C*H$_3$ (NHC)), 16.3 (NCH$_2$*C*H$_3$ (NHC)), 17.2 (NCH$_2$*C*H$_3$ (NHC)), 23.1 (NC(CH(*C*H$_3$)$_2$)), 23.5 (NC(CH(*C*H$_3$)$_2$)), 23.7 (Ar-*o*-CH(*C*H$_3$)$_2$), 25.1 (Ar-*o*-CH(*C*H$_3$)$_2$), 25.3 (Ar-*o*-CH(*C*H$_3$)$_2$), 25.7 (Ar-*o*-CH(*C*H$_3$)$_2$), 26.5 (Ar-*o*-CH(*C*H$_3$)$_2$), 27.3 (Ar-*o*-CH(*C*H$_3$)$_2$), 27.7 (Ar-*o*-*C*H(CH$_3$)$_2$), 28.0 (Ar-*o*-*C*H(CH$_3$)$_2$), 28.1 (Ar-*o*-*C*H(CH$_3$)$_2$), 31.8 (NC(CH(*C*H$_3$)$_2$)), 42.6 (N*C*H$_2$CH$_3$ (NHC)), 44.4 (N*C*H$_2$CH$_3$ (NHC)), 84.4 (NC(CH(CH$_3$)$_2$)*CH*), 85.4 (NC(CH(CH$_3$)$_2$)*CH*), 123.1 (Ar-*C*), 123.7 (Ar-*C*), 123.9 (Ar-*C*), 124.1 (Ar-*C*), 124.4 (Ar-*C*), 124.6 (Ar-*C*), 125.1 (Ar-*C*), 125.2 (Ar-*C*), 141.9 (Ar-*C*), 142.7 (Ar-*C*), 142.9 (Ar-*C*), 144.0 (Ar-*C*), 145.5 (Ar-*C*), 176.8 ((N*C*(CH(CH$_3$)$_2$)CH), 179.4 (N*C*(CH(CH$_3$)$_2$)CH), 183.2 ((RN)$_2$*C*: (NHC)). ^{1}H NMR (499.9 MHz, toluene-d_8, 398 K) δ = 0.98 (br d, 48H, Ar-*o*-CH(C*H*$_3$)$_2$ and NC(CH(C*H*$_3$)$_2$)), 1.15 (d, J_{HH} = 6.7 Hz, 24H, Ar-*o*-CH(C*H*$_3$)$_2$), 1.74 (br s, 6H, NC(C*H*$_3$)$_2$ (NHC)), 2.44 (sept, J_{HH} = 6.7 Hz, 4H, NC(C*H*(CH$_3$)$_2$)), 3.02 (br sept, 8H, Ar-*o*-C*H*(CH$_3$)$_2$), 4.28 (br q, 4H, NC*H*$_2$CH$_3$ (NHC)), 4.83 (s, 2H, NC(CH(CH$_3$)$_2$)C*H*). Note, some ligand aryl resonances are masked by residual solvent.

[{(^{iPrDip}NacNac)Mg(^{iPr}NHC)}(μ-S){Mg(^{iPrDip}NacNac)}] 6c

Method 1: [{(^{iPrDip}NacNac)Mg}$_2$(μ-S)] **1** (10 mg, 9.7 μmol) and ^{iPr}NHC ({MeCN*i*Pr}$_2$C:, 1.75 mg, 9.7 μmol) were dissolved in benzene-d_6 (0.5 mL) at 20 °C, yielding a colourless solution. Analysis by ^{1}H NMR spectroscopy showed immediate consumption of starting materials and formation of **6c**. *Method 2:* [{(^{iPrDip}NacNac)Mg}$_2$] **1** (100 mg, 0.100 mmol) and 1,3-diisopropyl-4,5-dimethyl-imidazole-2-thione (^{iPr}NHC=S, 21.3 mg, 0.100 mmol) were dissolved in *n*-hexane (30 mL) at 20 °C and stirred for 16 hours, during which time a colour change from yellow to colourless was observed. Due to high product solubility and tendency for precipitation of uncoordinated [{(^{iPrDip}NacNac)Mg}$_2$(μ-S)] **4**, no solid/pure **6c** could be isolated. ^{1}H NMR (499.9 MHz, toluene-d_8,

373 K) δ = 0.99 (d, J = 6.6 Hz, 24H, NCCH(CH_3)$_2$), 1.06 (d, J = 6.9 Hz, 24H, Ar-o-CH(CH_3)$_2$), 1.18 (d, J = 6.9 Hz, 24H, Ar-o-CH(CH_3)$_2$), 1.24 (d, 7.1 Hz, 8H, NCH(CH_3)$_2$ (^{iPr}NHC)), 1.43 (d, J = 6.6 Hz, excess (>12H), NCH(CH_3)$_2$ (^{iPr}NHC=S)), 1.75 (s, 4H, NCCH_3 (^{iPr}NHC)), 1.81 (br s, excess (>12H), NCH(CH_3)$_2$ (^{iPr}NHC=S)), 2.50 (sept, J = 6.9 Hz, 4H, NC(CH(CH$_3$)$_2$), 2.98 (sept, J = 6.9 Hz, 8H, Ar-o-CH(CH$_3$)$_2$), 4.03 (sept, J = 6.6 Hz, excess (>2H), NCH(CH$_3$)$_2$ (^{iPr}NHC=S)), 4.86 (s, 2H, NC(CH(CH$_3$)$_2$)CH). 5.64 (br sept, 2H, NCH(CH$_3$)$_2$ (^{iPr}NHC)) Ligand aryl resonances masked by residual solvent. ^{13}C{^{1}H} NMR (125.7 MHz, toluene-d_8, 398 K): δ = 8.9 (NCCH$_3$ (NHC)), 23.1 (NC(CH(CH_3)$_2$)), 23.7 (Ar-o-CH(CH$_3$)$_2$), 24.6 (NCH(CH$_3$)$_2$ (^{iPr}NHC)), 26.3 (Ar-o-CH(CH$_3$)$_2$), 28.4 (Ar-o-CH(CH$_3$)$_2$), 32.0 (NC(CH(CH$_3$)$_2$), 48.9 (NCH(CH$_3$)$_2$ (^{iPr}NHC)), 85.6 (NC(CH(CH$_3$)$_2$)CH), 124.0 (Ar-C), 137.7 (Ar-C), 143.1 (Ar-C), 179.8 (NC(CH(CH$_3$)$_2$)CH). A resonance for the carbenic carbon atom was too weak to be observed in the ^{13}C{^{1}H} NMR spectrum.

[{(^{iPrDip}NacNac)Mg}$_2$(μ-SN$_3$Ad)] 7

[{(^{iPrDip}NacNac)Mg}$_2$(μ-S)] **4** (20 mg, 0.019 mmol) and 1-adamantylazide (3.44 mg, 0.019 mmol) were slurried in benzene-d_6 (0.5 mL) at 20 °C in a J. Youngs NMR tube, yielding a colourless solution. Heating to 80 °C followed by slow cooling to 20 °C afforded colourless crystals of **7**. Concentration of the supernatant solution to *ca.* 0.2 mL and storing at 5 °C for two days afforded a second crop of **7**. [Note: heating was employed to aid efficient dissolution and recrystallisation, but was not required for the reaction to proceed.] Crystals suitable for X-ray crystallographic analysis were grown from a concentrated benzene-d_6 solution. Yield (isolated) = 15.8 mg (67 %). ^{1}H NMR (400.1 MHz, benzene-d_6, 294 K) δ = 0.95-1.01 (m, 48H, Ar-o-CH(CH_3)$_2$ and NCCH(CH_3)$_2$), 1.23 (d, J_{HH} = 6.7 Hz, 24H, Ar-o-CH(CH_3)$_2$), 1.59 (br s, 6H, Ad-CH_2), 1.90 (br s, 6H, Ad-CH_2), 2.01 (br s, 3H, Ad-CH), 2.54 (sept, J_{HH} = 6.6 Hz, 4H, NC(CH(CH$_3$)$_2$), 3.21 (sept, J_{HH} = 6.7 Hz, 8H, Ar-o-CH(CH$_3$)$_2$), 5.00 (s, 2H, NC(CH(CH$_3$)$_2$)CH), 7.09-7.11 (m, 6H, Ar-H), 7.16-7.20 (m, 6H, Ar-H). ^{13}C{^{1}H} NMR (100.5 MHz, benzene-d_6, 294 K): δ = 23.0 (NC(CH(CH$_3$)$_2$)), 24.2 (Ar-o-CH(CH$_3$)$_2$), 26.0 (Ar-o-CH(CH$_3$)$_2$), 28.1 (Ar-o-CH(CH$_3$)$_2$), 30.4 (Ad-CH), 31.9 (NC(CH(CH$_3$)$_2$)), 36.8 (Ad-CH$_2$), 43.2 (Ad-CH$_2$), 86.0 (NC(CH(CH$_3$)$_2$)CH), 124.2 (Ar-C), 125.4 (Ar-C), 142.9 (Ar-C), 143.2 (Ar-C), 179.8 (NC(CH(CH$_3$)$_2$)CH).

1.3 Information on donor adducts of [{(^{iPrDip}NacNac)Mg}$_2$(μ-S)] 4

[(^{iPrDip}NacNac)Mg(OAd)}$_2$(μ-S)] 8a

[{(^{iPrDip}NacNac)Mg}$_2$(μ-S)] **4** (10 mg, 9.7 μmol) and 2-adamantanone (= OAd, 2.92 mg, 19.4 μmol, 2 equivs.) were dissolved in benzene-d_6 (0.5 mL) at 20 °C yielding a colourless solution. Analysis by ^{1}H NMR spectroscopy showed immediate consumption of starting materials and formation of **8a**. From one such reaction, a few crystals suitable for X-ray crystallographic analysis were grown from

a concentrated benzene-d_6 solution. Attempts to isolate bulk crops of **8a** proved difficult due to high product solubility and tendency for precipitation of uncoordinated [{(^{iPrDip}NacNac)Mg}$_2$(μ-S)] **4**. ^{1}H NMR (400.1 MHz, benzene-d_6, 294 K) δ = 1.00 (d, J_{HH} = 6.7 Hz, 24Hz, NC(CH(C*H*$_3$)$_2$)), 1.18 (d, J_{HH} = 6.8 Hz, 24Hz, Ar-*o*-CH(C*H*$_3$)$_2$), 1.32 (d, J_{HH} = 7.0 Hz, 24Hz, Ar-*o*-CH(C*H*$_3$)$_2$), 1.51 (br s, 2H, Ad-C*H*$_2$), 1.58 (br s, 2H, Ad-C*H*), 1.68 (br s, 2H, Ad-C*H*$_2$), 1.71 (br s, 2H, Ad-C*H*$_2$), 1.84 (br s, 2H, Ad-C*H*$_2$), 1.87 (br s, 2H, Ad-C*H*$_2$), 2.58 (sept, J_{HH} = 6.8 Hz, 4H, NC(C*H*(CH$_3$)$_2$)), 2.70 (br s, 2H, Ad-C*H*), 3.13 (sept, J_{HH} = 6.8 Hz, 8H, Ar-*o*-C*H*(CH$_3$)$_2$), 4.82 (s, 2H, NC(CH(CH$_3$)$_2$)C*H*), 7.12-7.14 (m, 8H, Ar-*H*) 7.17-7.20 (m, 4H, Ar-*H*). ^{13}C{^{1}H} NMR (100.5 MHz, benzene-d_6, 294 K): δ = 23.0 (NC(CH(*C*H$_3$)$_2$)), 24.0 (Ar-*o*-CH(*C*H$_3$)$_2$), 26.8 (Ar-*o*-CH(*C*H$_3$)$_2$), 27.4 (Ad-*C*H), 27.9 (Ar-*o*-*C*H(CH$_3$)$_2$), 31.7 (NC(*C*H(CH$_3$)$_2$)), 36.1 (Ad-*C*H$_2$), 39.8 (Ad-*C*H$_2$), 46.9 (Ad-*C*H), 84.8 (NC(CH(CH$_3$)$_2$)*C*H), 123.7 (Ar-*C*), 124.9 (Ar-*C*), 143.0 (Ar-*C*), 143.8 (Ar-*C*), 178.1 (N*C*(CH(CH$_3$)$_2$).

[{(^{iPrDip}NacNac)Mg(OCPh$_2$)}$_2$(μ-S)] 8b

[{(^{iPrDip}NacNac)Mg}$_2$(μ-S)] **4** (10 mg, 9.7 μmol) and benzophenone (3.54 mg, 19.4 μmol, 2 equivs.) were dissolved in benzene-d_6 (0.5 mL) at 20 °C yielding a colourless solution. Analysis by ^{1}H NMR spectroscopy showed immediate consumption of starting materials and formation of **8b**. Attempts to isolate bulk crops of **8b** proved difficult due to high product solubility and tendency for precipitation of uncoordinated [{(^{iPrDip}NacNac)Mg}$_2$(μ-S)] **4**. ^{1}H NMR (400.1 MHz, benzene-d_6, 294 K) δ = 1.02 (d, J_{HH} = 6.8 Hz, 24Hz, NC(CH(C*H*$_3$)$_2$)), 1.08 (d, J_{HH} = 6.8 Hz, 24Hz, Ar-*o*-CH(C*H*$_3$)$_2$), 1.17 (d, J_{HH} = 6.8 Hz, 24Hz, Ar-*o*-CH(C*H*$_3$)$_2$), 2.57 (sept, J_{HH} = 6.5 Hz, 4H, NC(C*H*(CH$_3$)$_2$)), 3.12 (sept, J_{HH} = 6.8 Hz, 8H, Ar-*o*-C*H*(CH$_3$)$_2$), 5.01 (s, 2H, NC(CH(CH$_3$)$_2$)C*H*), 7.03-7.19 (m, 12H, Ar-*H*). ^{13}C{^{1}H} NMR (100.5 MHz, benzene-d_6, 294 K): δ = 23.1 (NC(CH(*C*H$_3$)$_2$)), 23.7 (Ar-*o*-CH(*C*H$_3$)$_2$), 26.8 (Ar-*o*-CH(*C*H$_3$)$_2$), 27.9 (Ar-*o*-*C*H(CH$_3$)$_2$), 31.8 (NC(*C*H(CH$_3$)$_2$)), 85.4 (NC(CH(CH$_3$)$_2$)*C*H), 123.8 (Ar-*C*), 125.1 (Ar-*C*), 128.6 (Ar-*C*), 133.7 (Ar-*C*), 142.9 (Ar-*C*), 143.9 (Ar-*C*), 178.4 (N*C*(CH(CH$_3$)$_2$).

[{(^{iPrDip}NacNac)Mg(OC*i*Pr$_2$)}$_2$(μ-S)] 8c and formation of [{(^{iPrDip}NacNac)Mg(μ-SH)}$_2$] 9

[{(^{iPrDip}NacNac)Mg}$_2$(μ-S)] **1** (10 mg, 9.7 μmol) and diisopropylketone (O=C*i*Pr$_2$, 2.22 mg, 2.76 μL, 19.4 μmol, 2 equivs.) were dissolved in benzene-d_6 (0.5 mL) at 20 °C yielding a colourless solution. Analysis by ^{1}H NMR spectroscopy showed immediate consumption of starting materials and the formation of **8c** was suggested. However, please note that the ^{1}H NMR spectrum is only tentatively and partially assigned. An attempt to resolve the overlapping and broad resonances in the ^{1}H NMR spectrum using high temperature NMR spectroscopy was not undertaken due to the instability of the compound/mixture at elevated temperatures. ^{1}H NMR (400.1 MHz, benzene-d_6, 294 K) δ = 0.63 (br, 24H, OC(CH(C*H*$_3$)$_2$)$_2$), 0.92-1.01 (m, 15H, Ar-*o*-CH(C*H*$_3$)$_2$), 1.13-1.31 (m, 51H, Ar-*o*-CH(C*H*$_3$)$_2$ and

NC(CH(CH_3)$_2$)), 1.99 (br, 6H, Ar-*o*-CH(CH_3)$_2$ or NC(CH(CH_3)$_2$)), 2.68 (br sept, 4H, Ar-*o*-C*H*(CH$_3$)$_2$), 3.09 (br sept, 4H, Ar-*o*-C*H*(CH$_3$)$_2$), 3.22 (br sept, 4H, NC(C*H*(CH$_3$)$_2$)), 3.33-3.37 (br m, 4H, OC(C*H*(CH$_3$)$_2$)$_2$), 5.00 (br s, 2H, NC(CH(CH$_3$)$_2$)C*H*). Ligand aryl resonances masked by residual solvent. Attempts to isolate bulk crops of **8c** proved difficult due to high product solubility and tendency for precipitation of uncoordinated [{(^{iPrDip}NacNac)Mg}$_2$(μ-S)] **4**.

Decomposition was observed according to ^{1}H NMR spectroscopy upon heating a benzene-*d*$_6$ solution of **8c** to 80 °C. Initial formation of [{(^{iPrDip}NacNac)Mg(μ-SH}$_2$] **9** was suggested as part of a reaction mixture after 6 hours of heating, followed by the subsequent decomposition of **9** to ^{iPrDip}NacNacH within the mixture. Crystals of **9** suitable for X-ray crystallographic analysis were grown from a concentrated benzene-*d*$_6$ solution. Due to complex nature of the obtained ^{1}H NMR spectrum, accurate assignment of **9** was not possible, although a resonance at -0.99 ppm was attributed to the Mg-S*H* group, see Figure S48, plus Figure S59 for the molecular structure from single crystal X-ray diffraction.

[{(^{iPrDip}NacNac)Mg(CNCy)}$_2$(μ-S)] 8d

[{(^{iPrDip}NacNac)Mg}$_2$(μ-S)] **4** (10 mg, 9.7 μmol) and cyclohexylisocyanide (2.12 mg, 2.39 μL, 19.4 μmol, 2 equivs.) were dissolved in benzene-*d*$_6$ (0.5 mL) at 20 °C in a J. Youngs NMR tube, yielding a colourless solution. Analysis by ^{1}H NMR spectroscopy showed immediate consumption of starting materials and formation of **8d**. Attempts to isolate bulk crops of **8d** proved difficult due to high product solubility and tendency for precipitation of uncoordinated [{(^{iPrDip}NacNac)Mg}$_2$(μ-S)] **4**. ^{1}H NMR (400.1 MHz, benzene-*d*$_6$, 294 K) δ = 0.96-0.99 (m, 4H, Cy-*o*-CH_2 and Cy-*p*-CH_2), 1.03 (d, J_{HH} = 6.7 Hz, 24H, NCCH(CH_3)$_2$), 1.22 (d, J_{HH} = 7.0 Hz, 24H, Ar-*o*-CH(CH_3)$_2$), 1.32 (d, 7.0 Hz, 24H, Ar-*o*-CH(CH_3)$_2$), 1.41-1.48 (m, 6H, Cy-*m*-CH_2 and Cy-*p*-CH_2), 2.61 (sept, J_{HH} = 6.7 Hz, 4H, NC(C*H*(CH$_3$)$_2$), 3.06 (br t, 1H, CNC*H*), 3.22 (sept, J_{HH} = 6.9 Hz, 8H, Ar-*o*-C*H*(CH$_3$)$_2$), 4.83 (s, 2H, NC(CH(CH$_3$)$_2$)C*H*), 7.15-7.23 (m, 12H, Ar-*H*). ^{13}C{^{1}H} NMR (100.5 MHz, benzene-*d*$_6$, 294 K): δ = 22.1 (Cy-*m*-CH$_2$), 23.0 (NC(CH(*C*H$_3$)$_2$)), 24.1 (Ar-*o*-CH(*C*H$_3$)$_2$), 24.9 (Cy-*o*-*C*H$_2$), 26.9 (Ar-*o*-CH(*C*H$_3$)$_2$), 28.1 (Ar-*o*-*C*H(CH$_3$)$_2$), 31.5 (NC(*C*H(CH$_3$)$_2$)), 31.6 (Cy-*p*-*C*H$_2$), 52.1 (CN*C*H), 84.0 (NC(CH(CH$_3$)$_2$)*C*H), 123.5 (Ar-*C*), 124.8 (Ar-*C*), 143.0 (Ar-*C*), 144.0 (Ar-*C*), 177.7 (N*C*(CH(CH$_3$)$_2$)CH).

2 NMR Spectroscopy

NMR spectra were recorded in deuterated benzene or toluene and further details are given in the figure captions. In some samples, resonances of residual solvent and silicone grease (as impurities) may be present; for example silicone grease (literature values: ^{1}H: 0.29 ppm, ^{13}C{^{1}H}: 1.38), toluene (^{1}H: 2.11, 7.02, 7.13 ppm, ^{13}C{^{1}H}: 21.10, 125.68, 128.56, 129.33, 137.91), and *n*-hexane (^{1}H: 0.89, 1.24, ^{13}C{^{1}H}: 14.32, 23.04, 31.96).[4] Estimated barriers were determined using the coalescence temperature, T_c, and the frequencies of the unmerged resonances.[5]

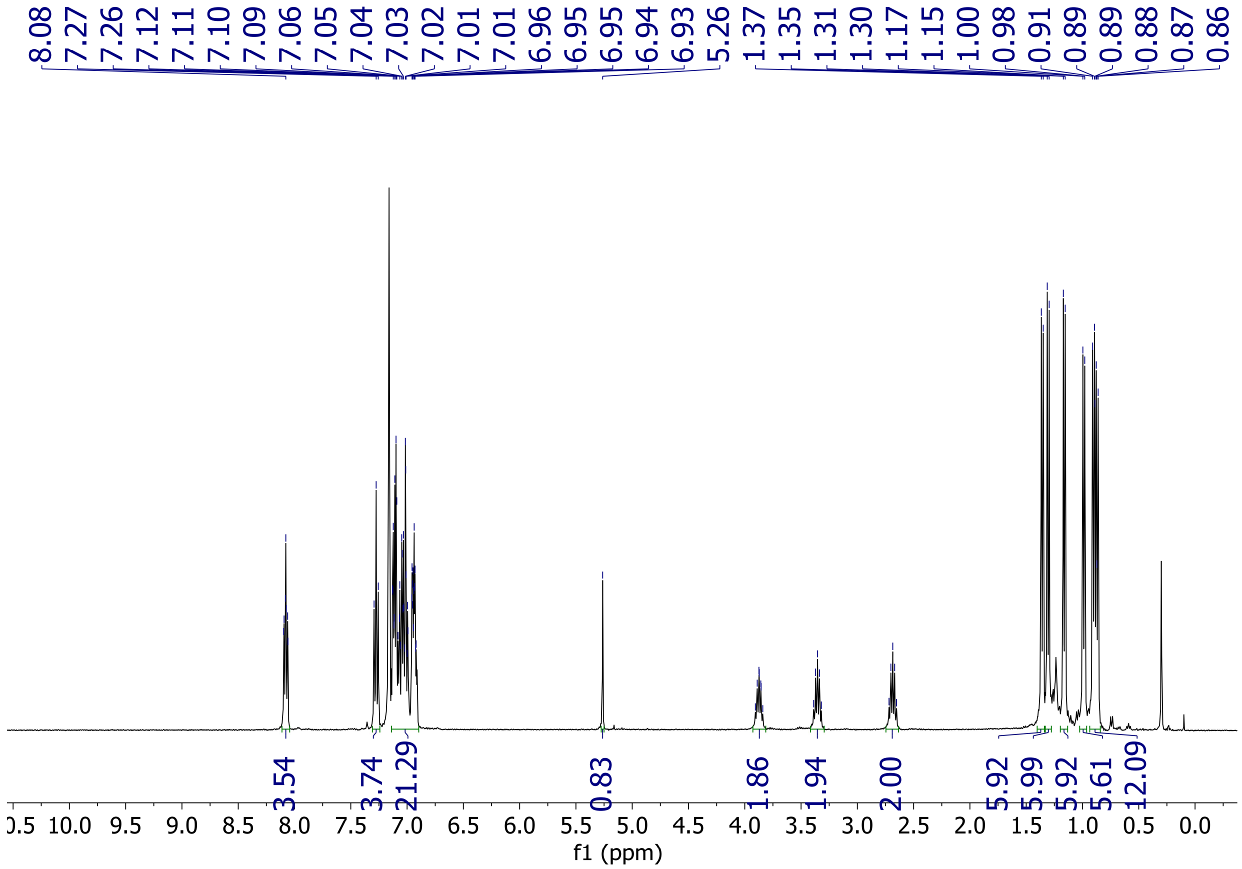

Figure S1. ^{1}H NMR spectrum (400.1 MHz, C_6D_6, 294 K) of [(^{iPrDip}NacNac)Mg($OPPh_3$)($OPPh_2$)] **3**.

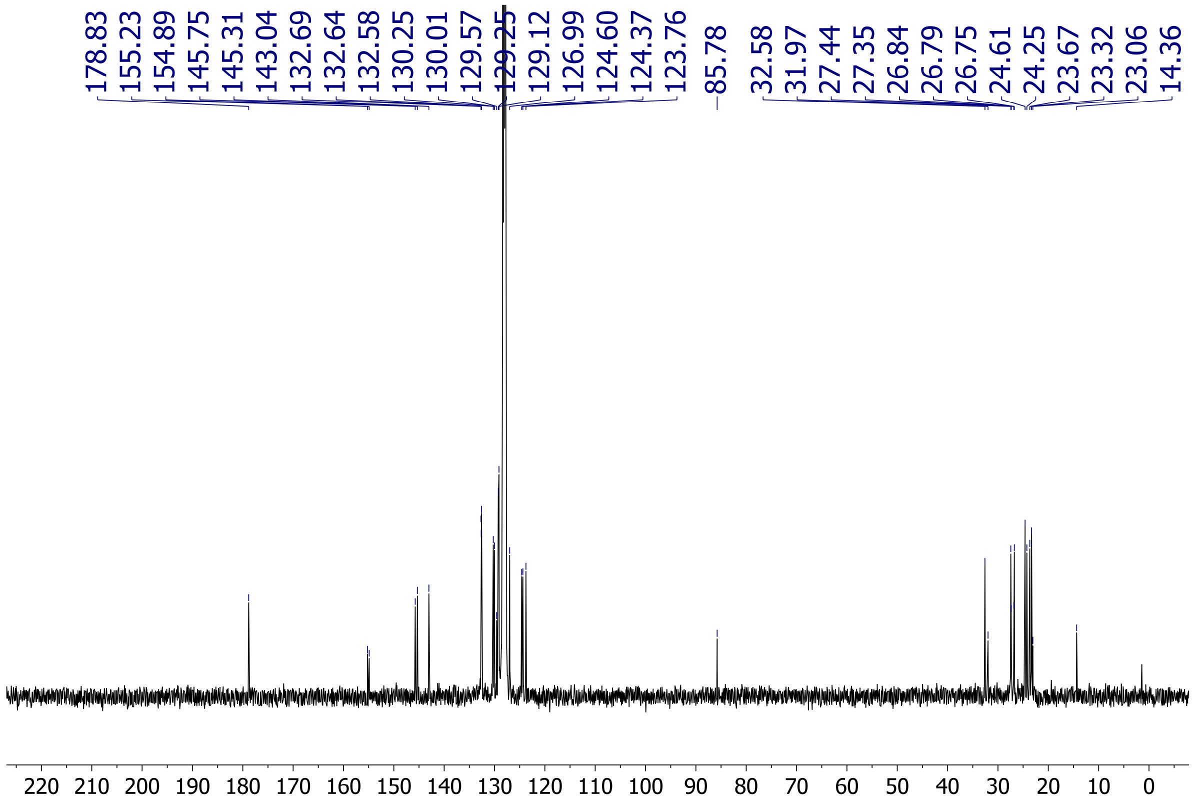

Figure S2. $^{13}C\{^{1}H\}$ NMR spectrum (100.5 MHz, C_6D_6, 294 K) of **3**.

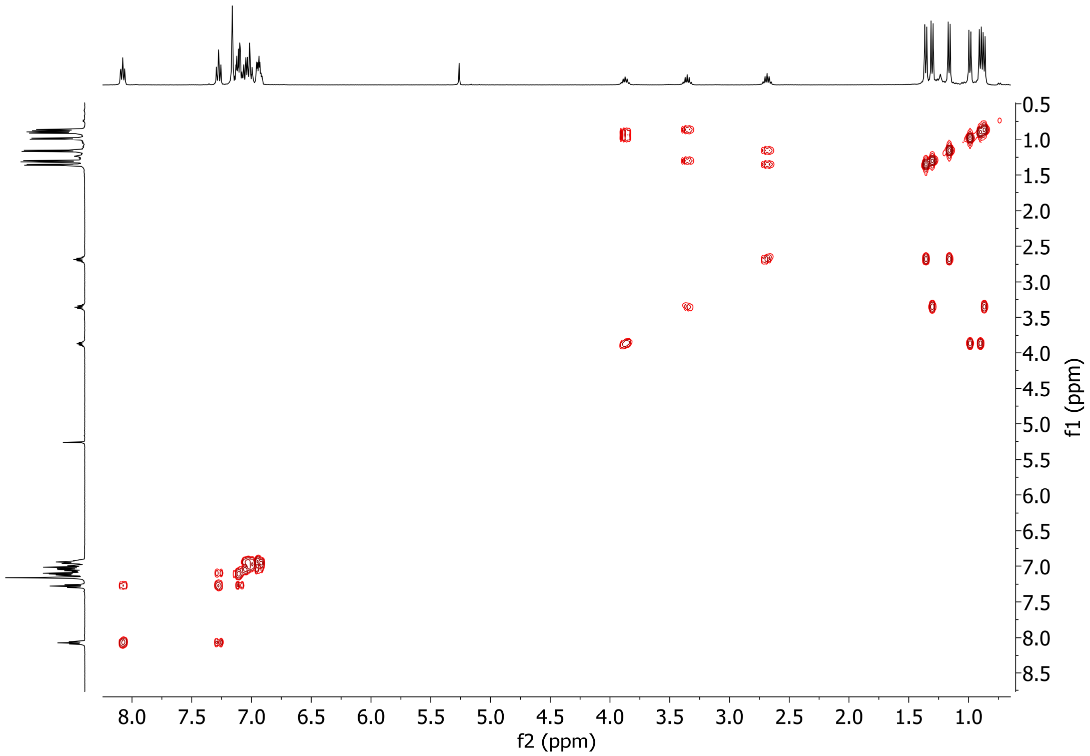

Figure S3. ^{1}H-^{1}H COSY NMR spectrum of **3**.

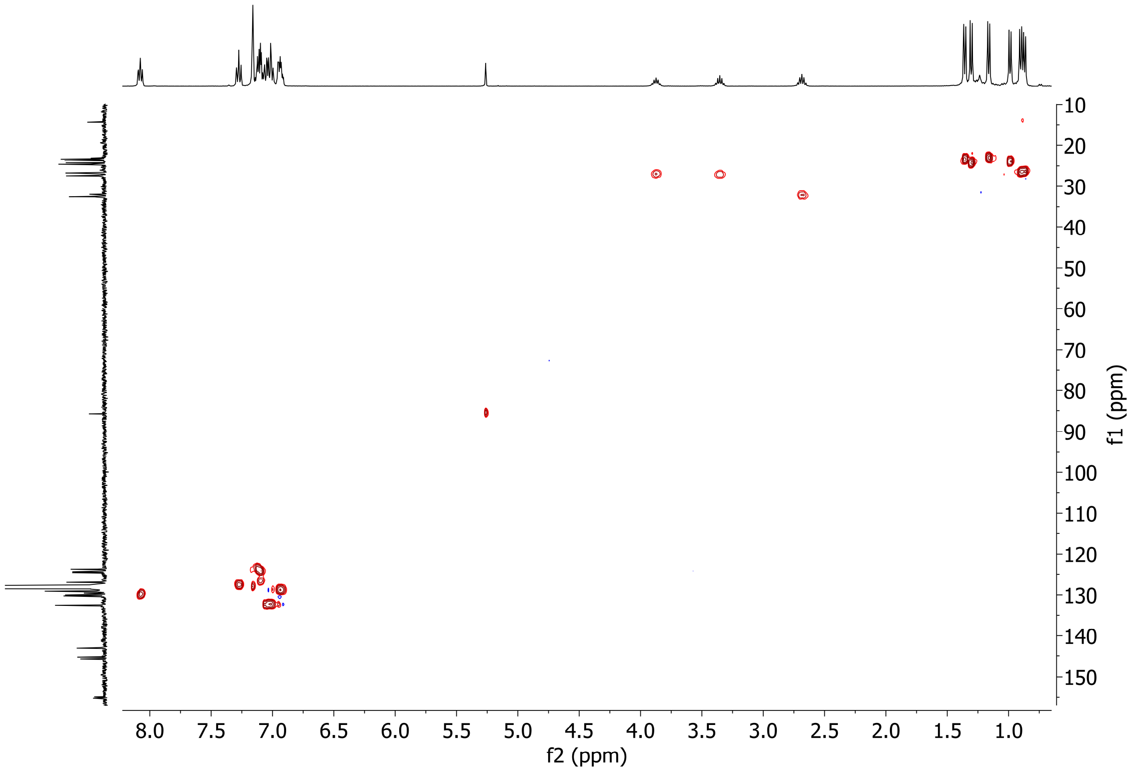

Figure S4. ^{1}H-^{13}C HSQC NMR spectrum of **3**.

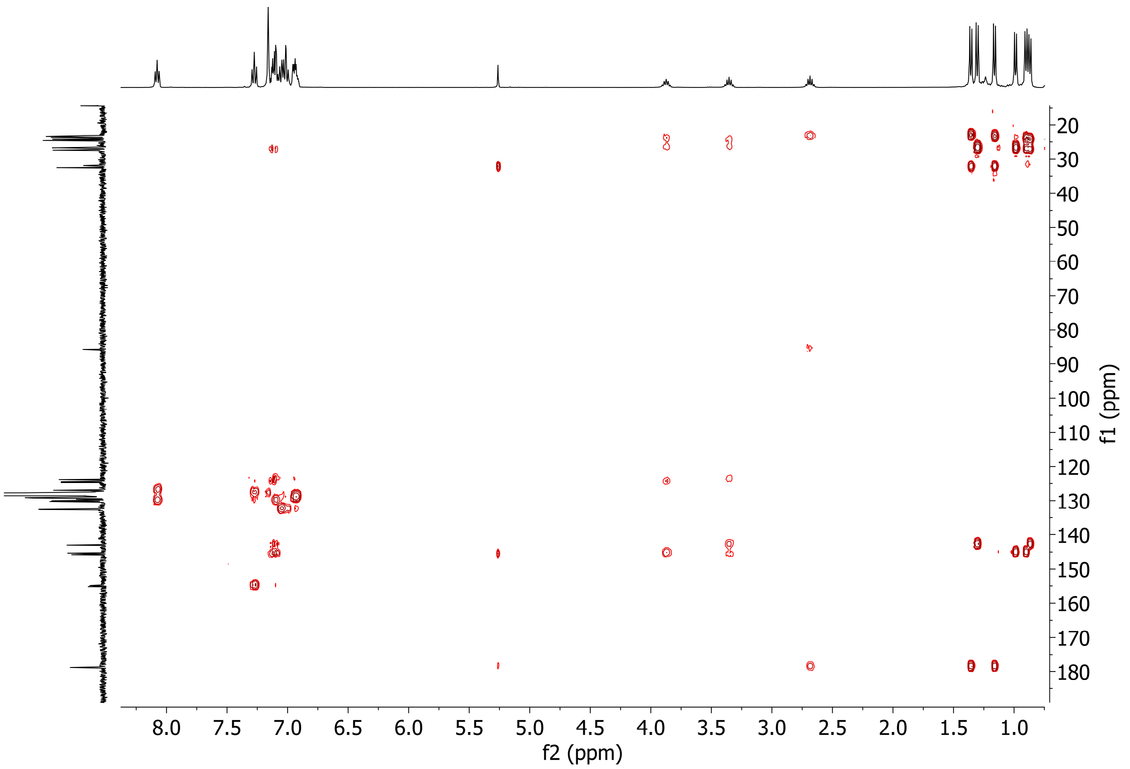

Figure S5. ^{1}H-^{13}C HMBC NMR spectrum of **3**.

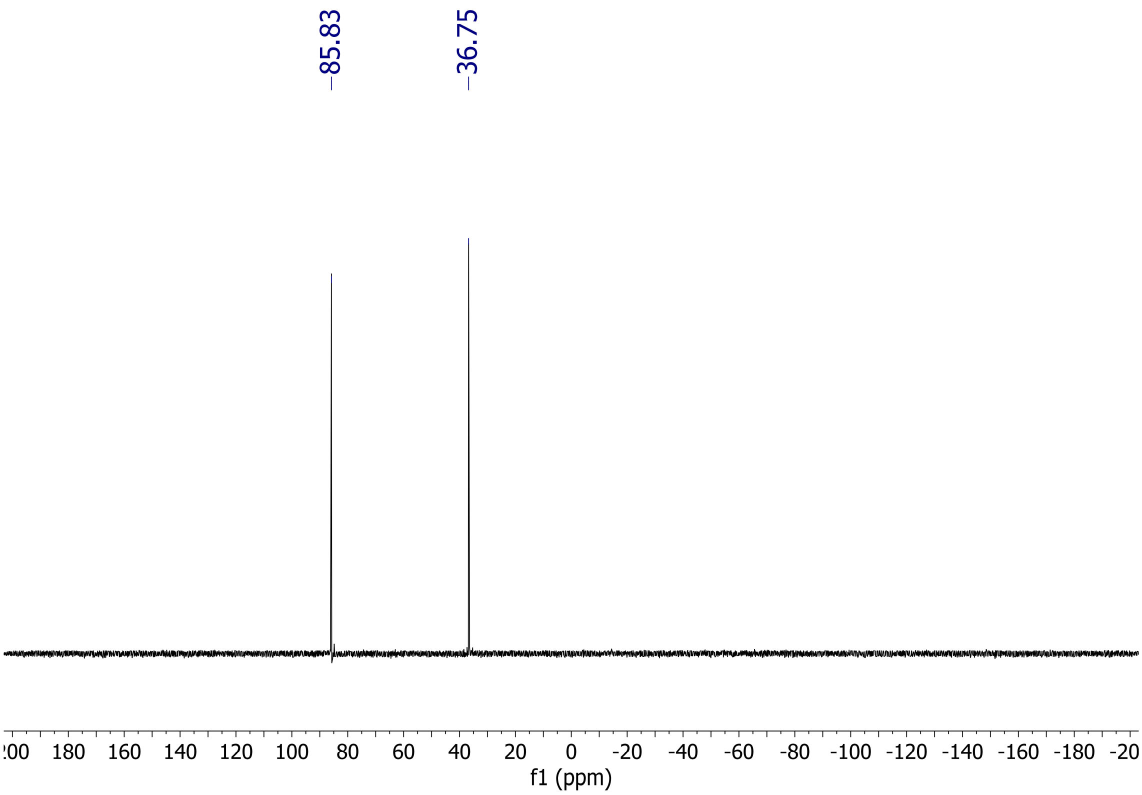

Figure S6. ^{31}P{^{1}H} NMR spectrum (162.0 MHz, C_6D_6, 294 K) of **3**.

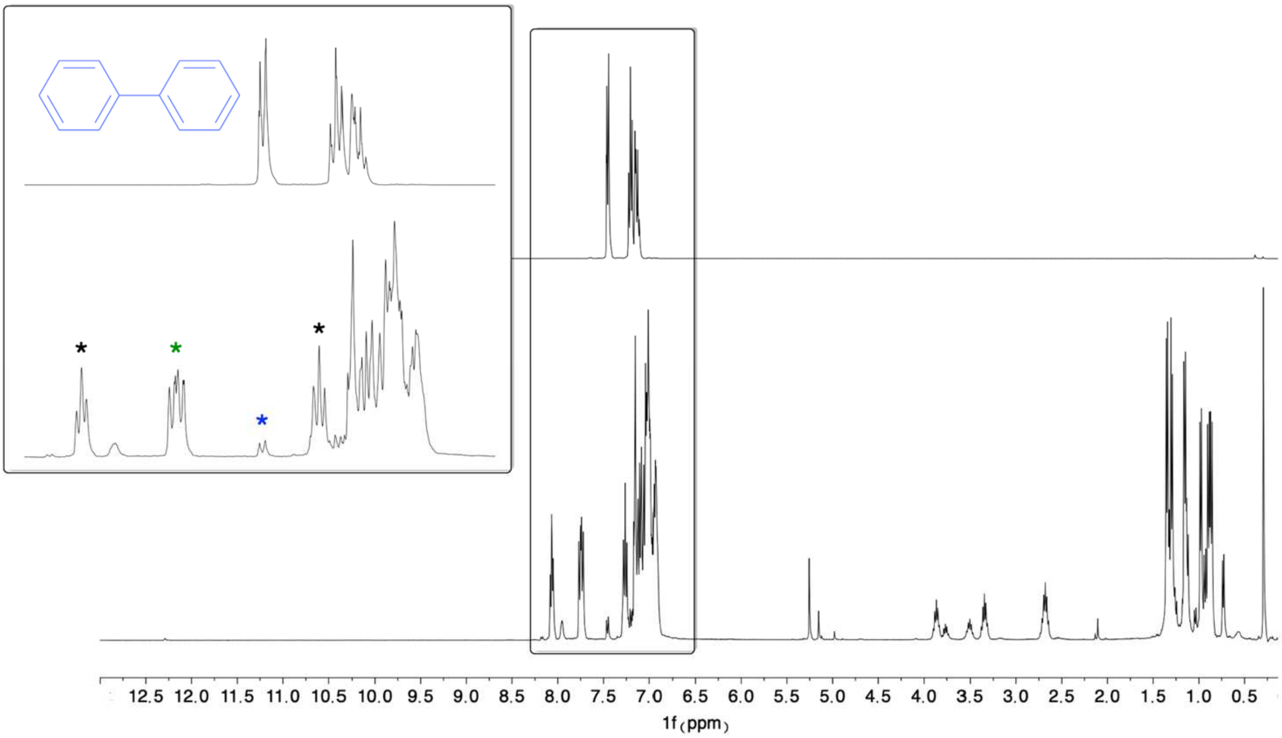

Figure S7. ^{1}H NMR spectrum (400.1 MHz, C_6D_6, 294 K) of *in-situ* reaction of $[\{(^{iPrDip}NacNac)Mg\}_2]$ **1** with $OPPh_3$ forming $[(^{iPrDip}NacNac)Mg(OPPh_3)(OPPh)_2]$ **3** after approx. 2 d at 100°C (bottom), and ^{1}H NMR spectrum of biphenyl (top, same conditions) for comparison. The insert shows the aromatic region and the black asterisks mark **3**, the green asterisk marks $OPPh_3$, and the blue asterisk marks biphenyl.

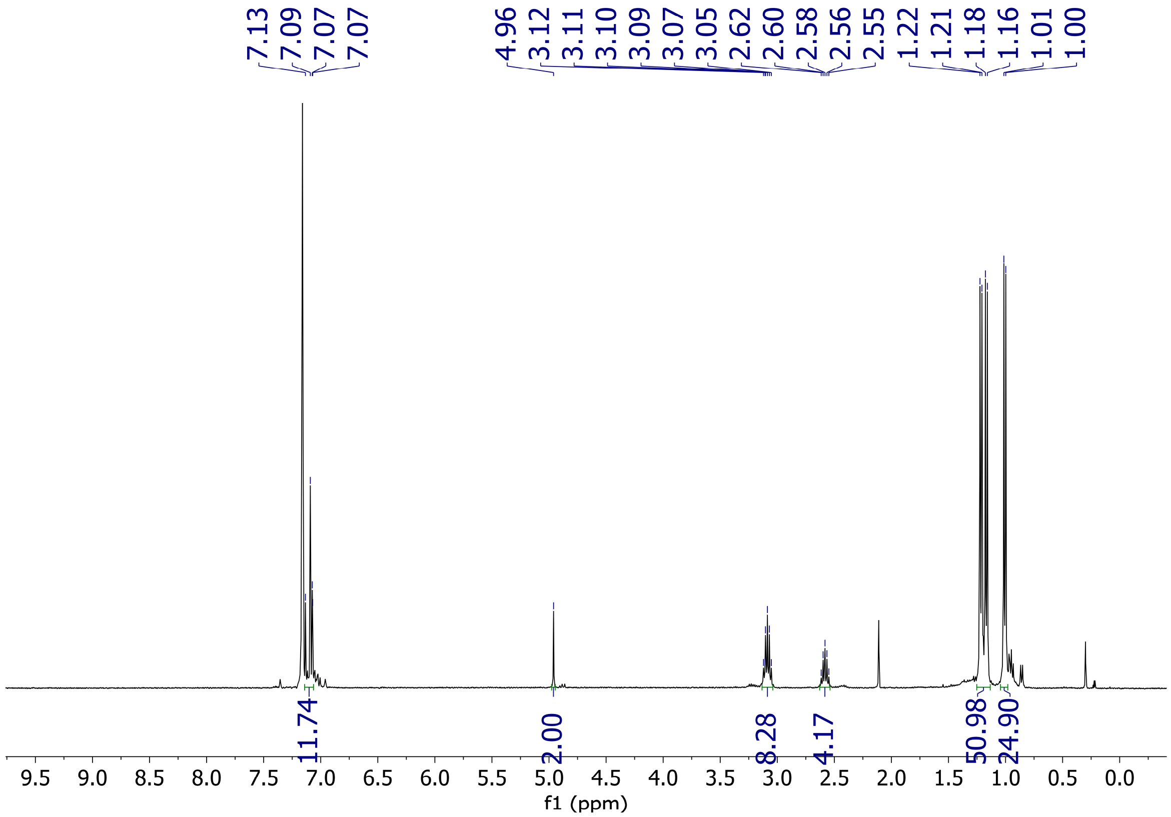

Figure S8. ^{1}H NMR spectrum (400.1 MHz, C_6D_6, 294 K) of [{(^{iPrDip}NacNac)Mg}$_2$(μ-S)] **4**.

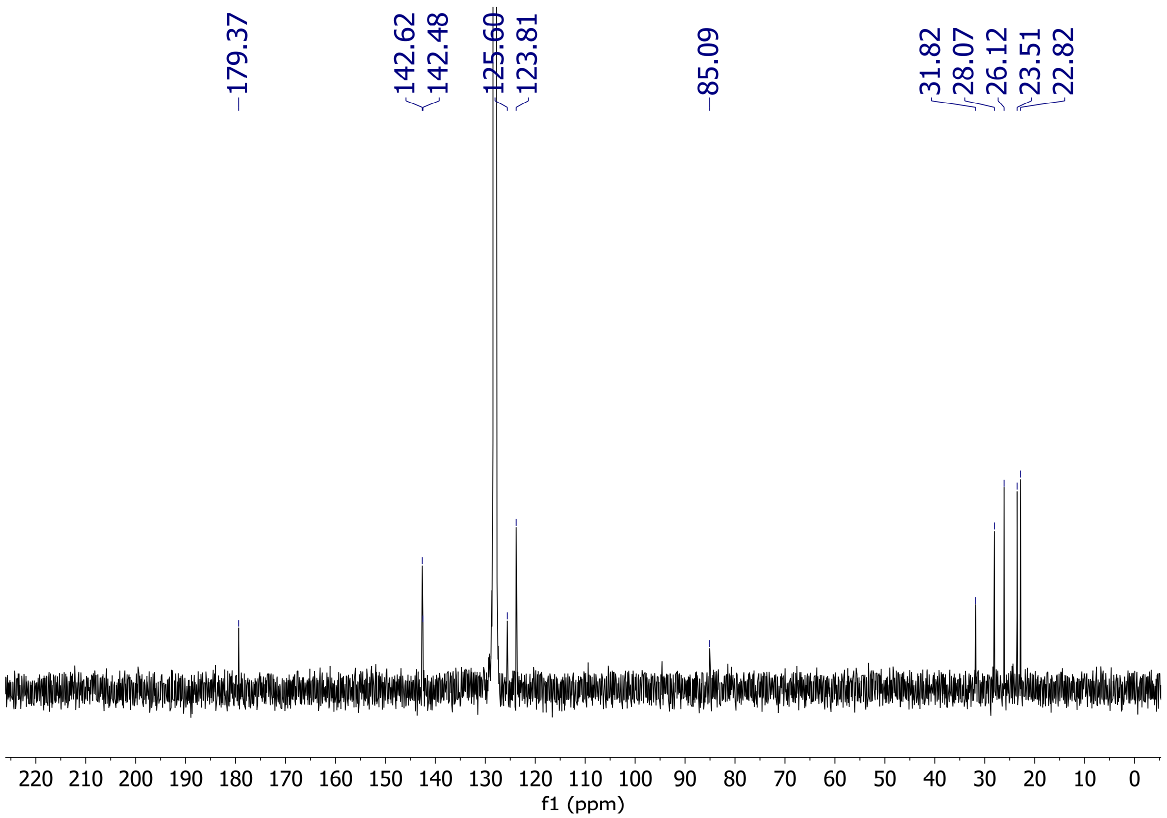

Figure S9. ^{13}C{^{1}H} NMR spectrum (100.5 MHz, C_6D_6, 294 K) of **4**.

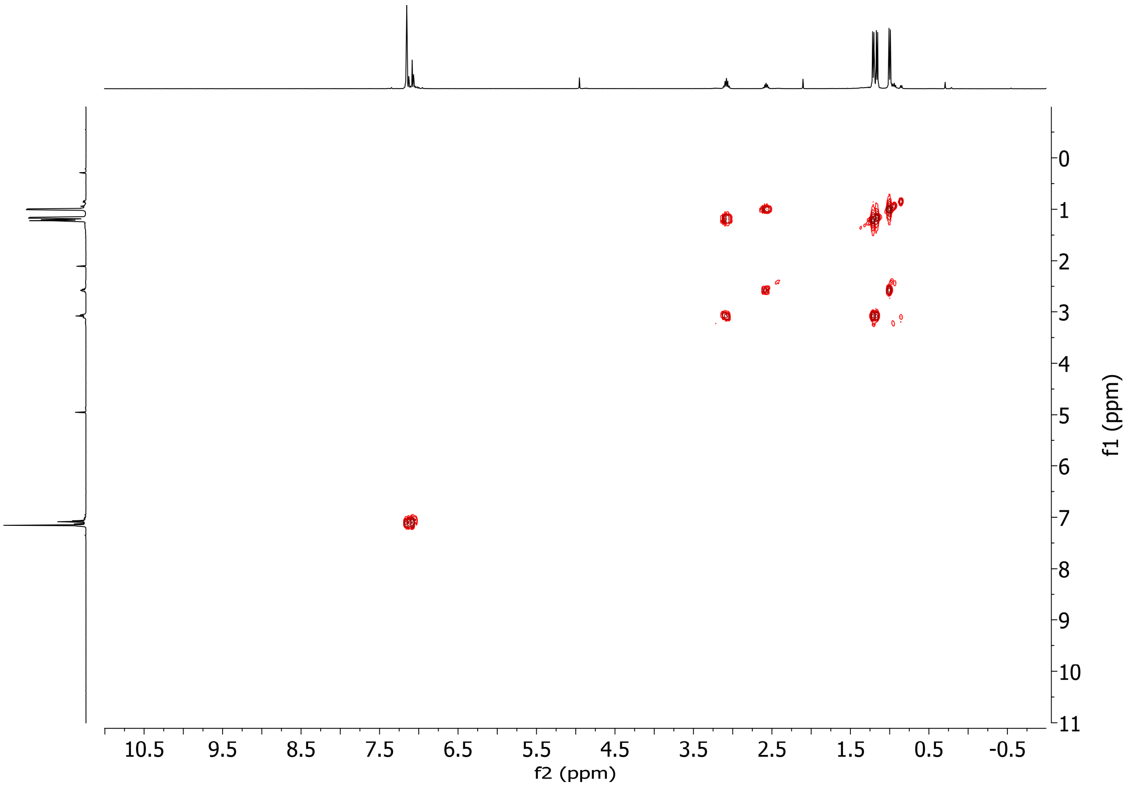

Figure S10. ^{1}H-^{1}H COSY NMR spectrum of **4**.

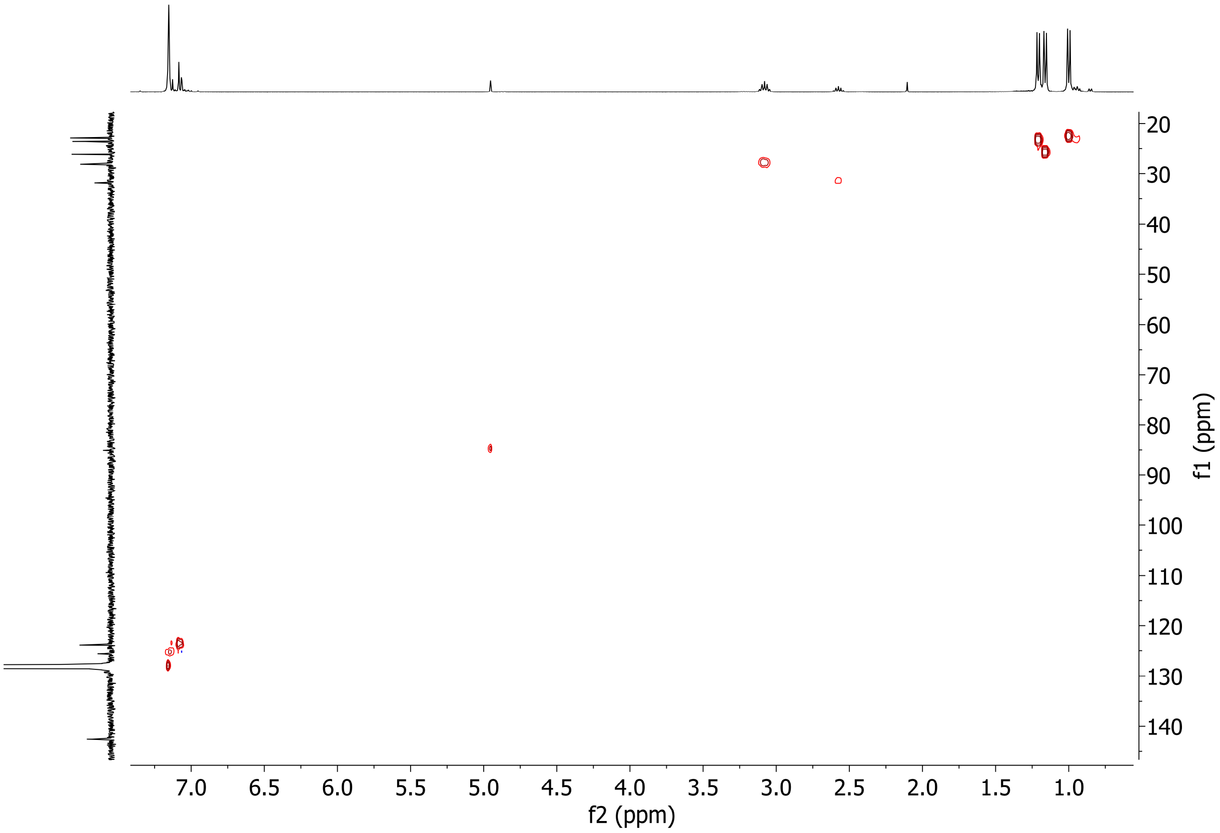

Figure S11. ^{1}H-^{13}C HSQC NMR spectrum of **4**.

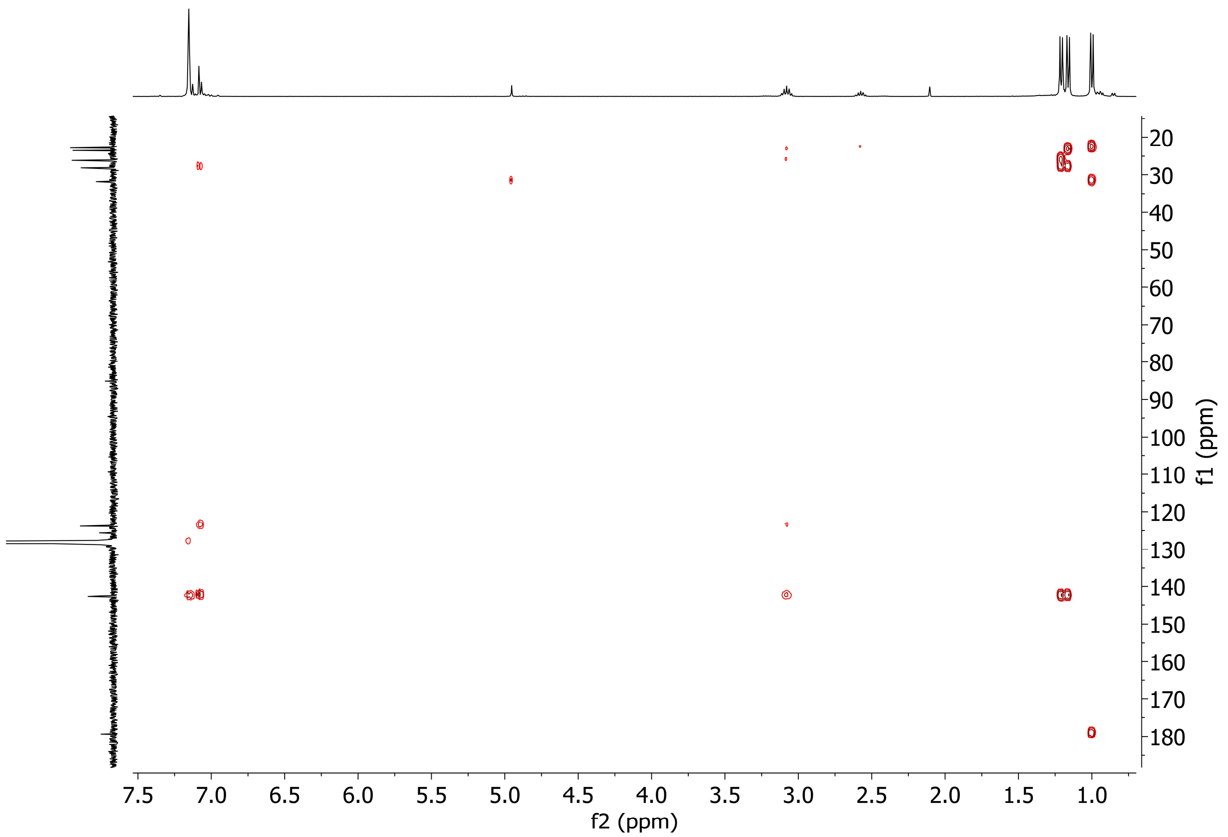

Figure S12. ^{1}H-^{13}C HMBC NMR spectrum of **4**.

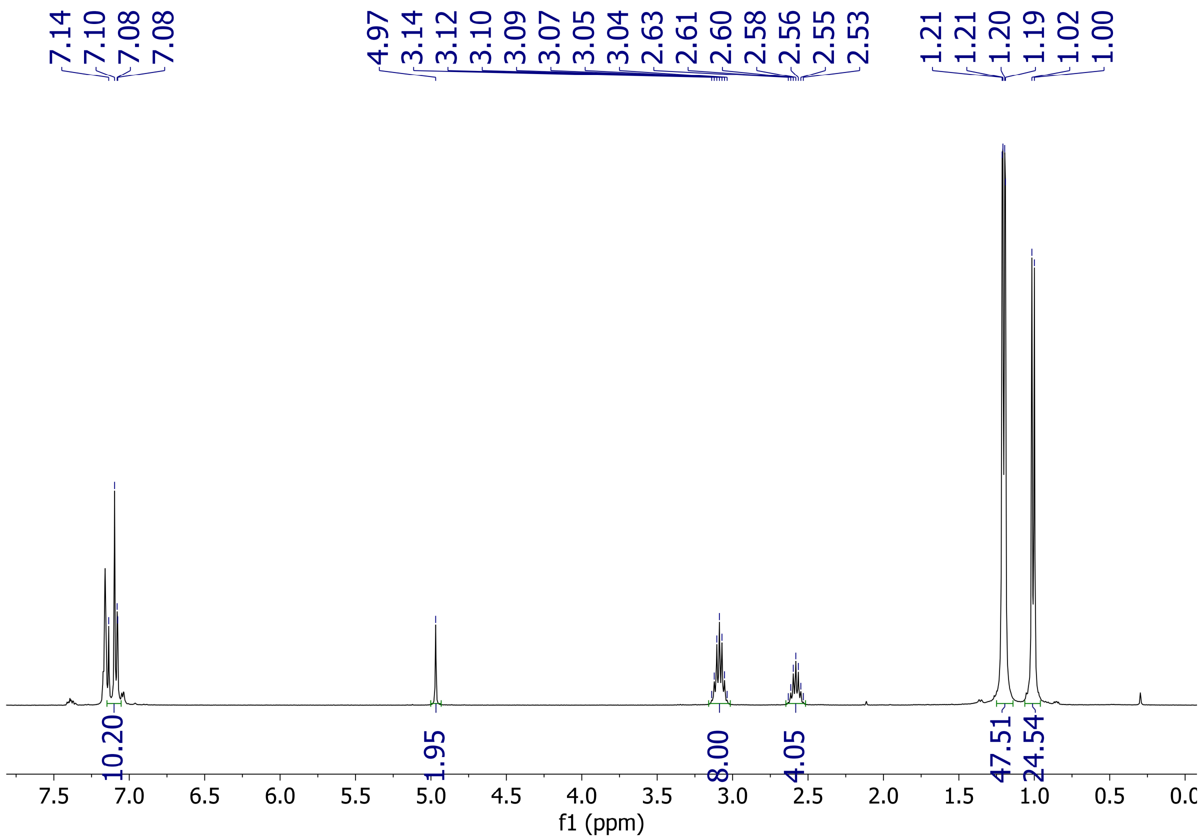

Figure S13. ^{1}H NMR spectrum (400.1 MHz, C_6D_6, 294 K) of [{(^{iPrDip}NacNac)Mg}$_2$(μ-Se)] **5**.

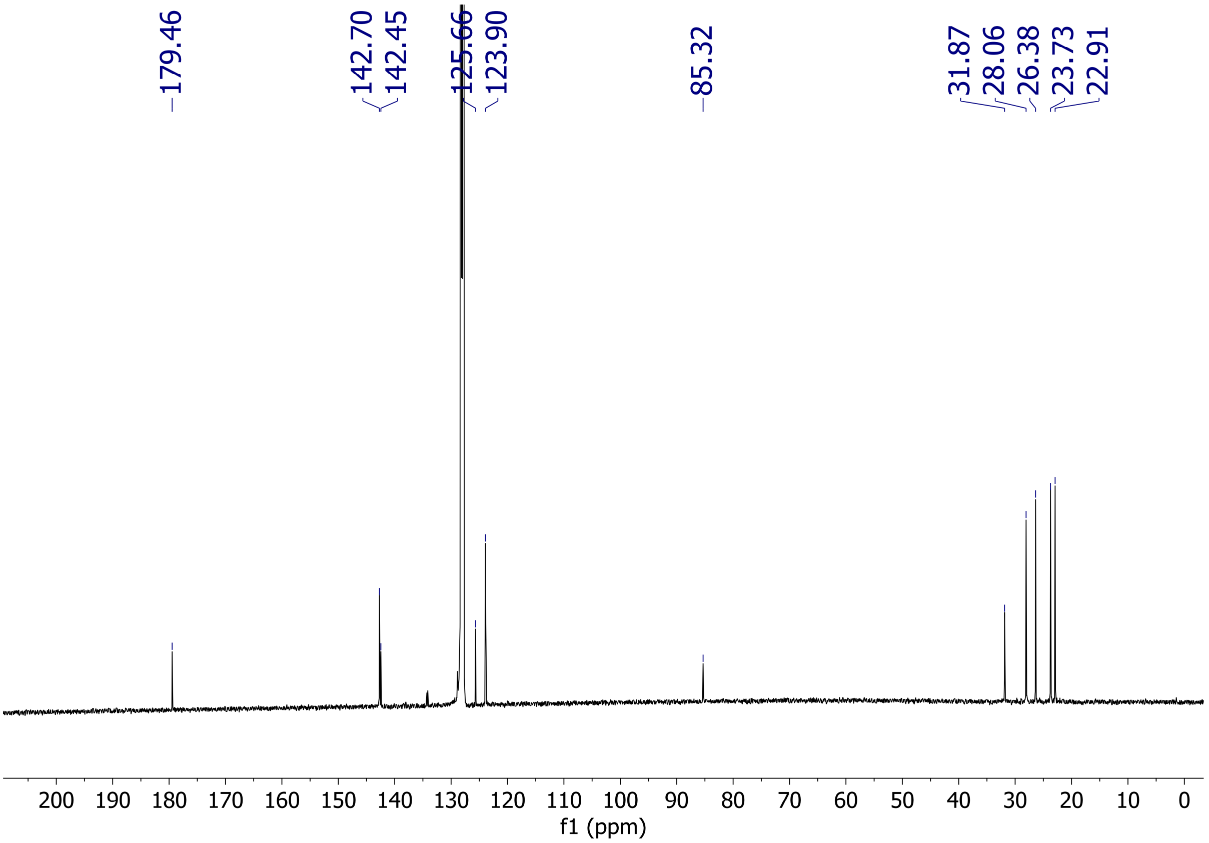

Figure S14. $^{13}C\{^{1}H\}$ NMR spectrum (100.5 MHz, C_6D_6, 294 K) of **5**.

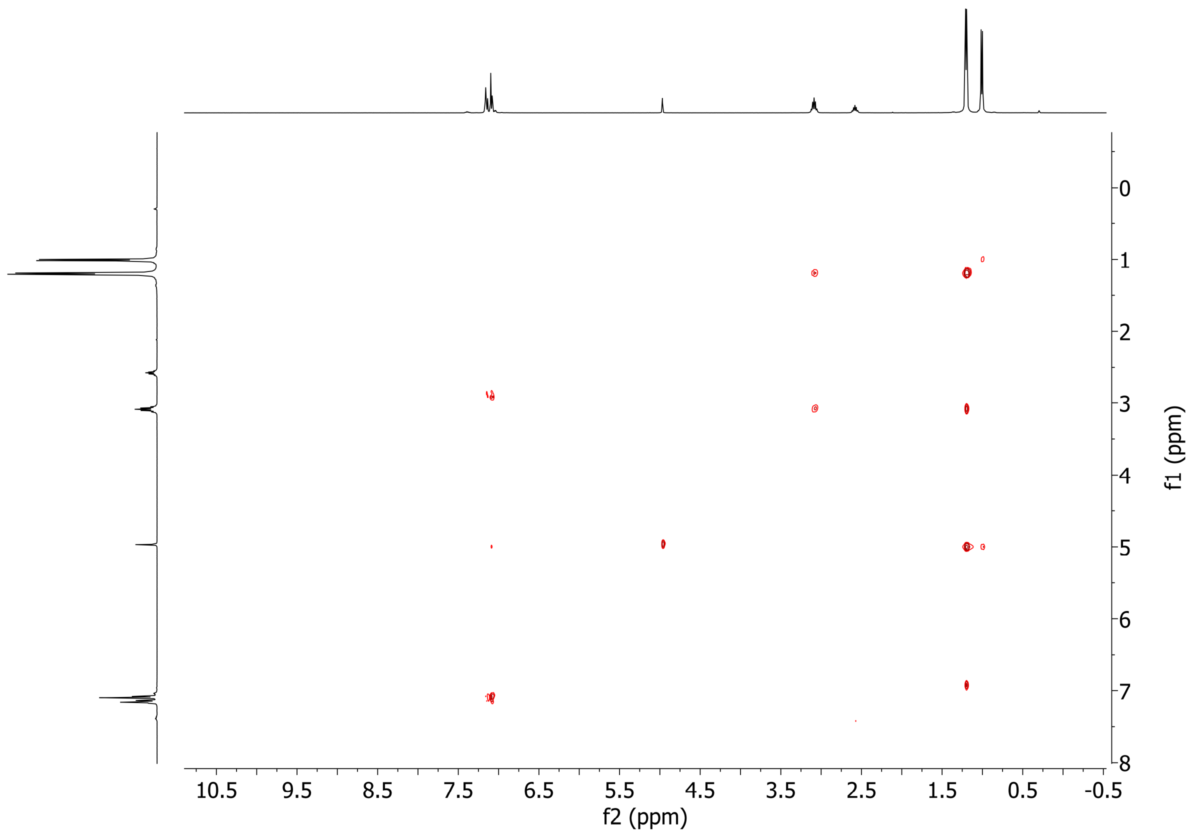

Figure S15. ^{1}H-^{1}H COSY NMR spectrum of **5**.

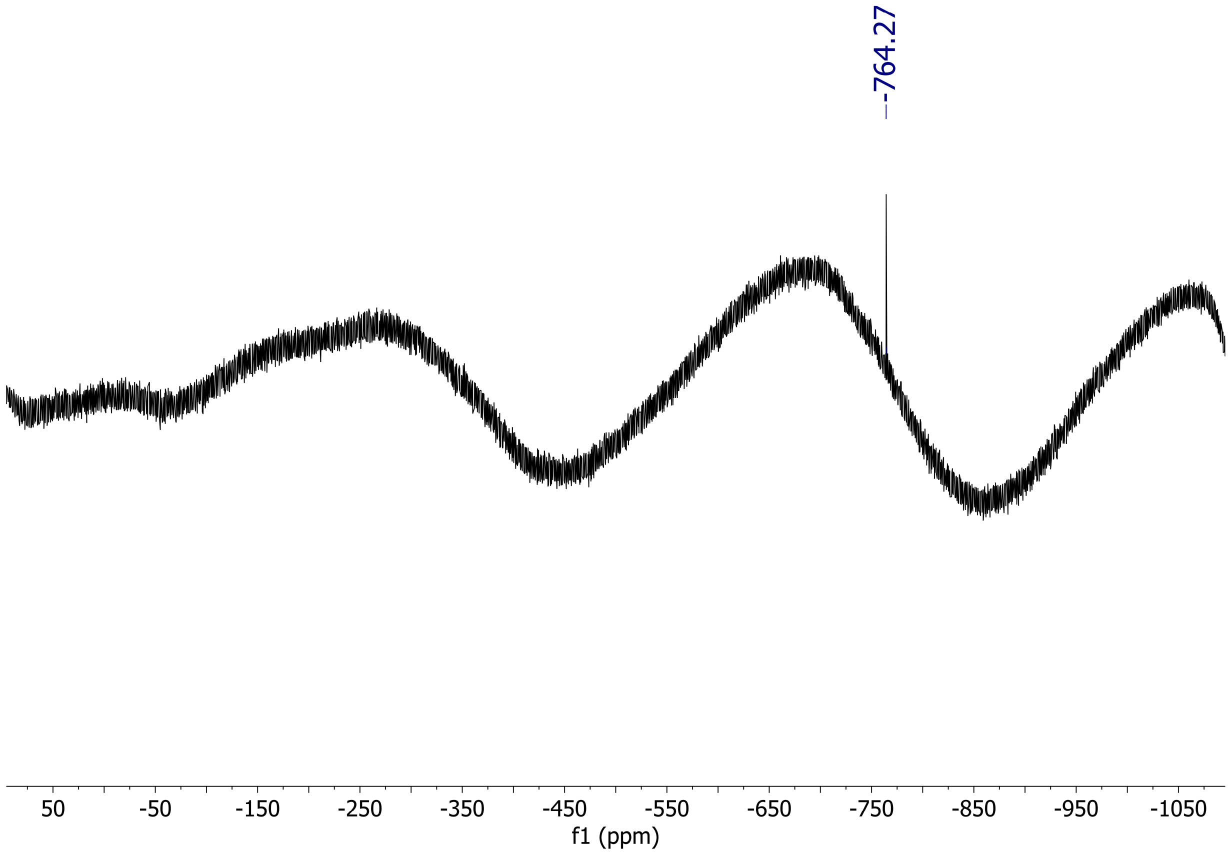

Figure S16. $^{77}Se\{^{1}H\}$ NMR spectrum (95.4 MHz, C_6D_6, 294 K) of **5**.

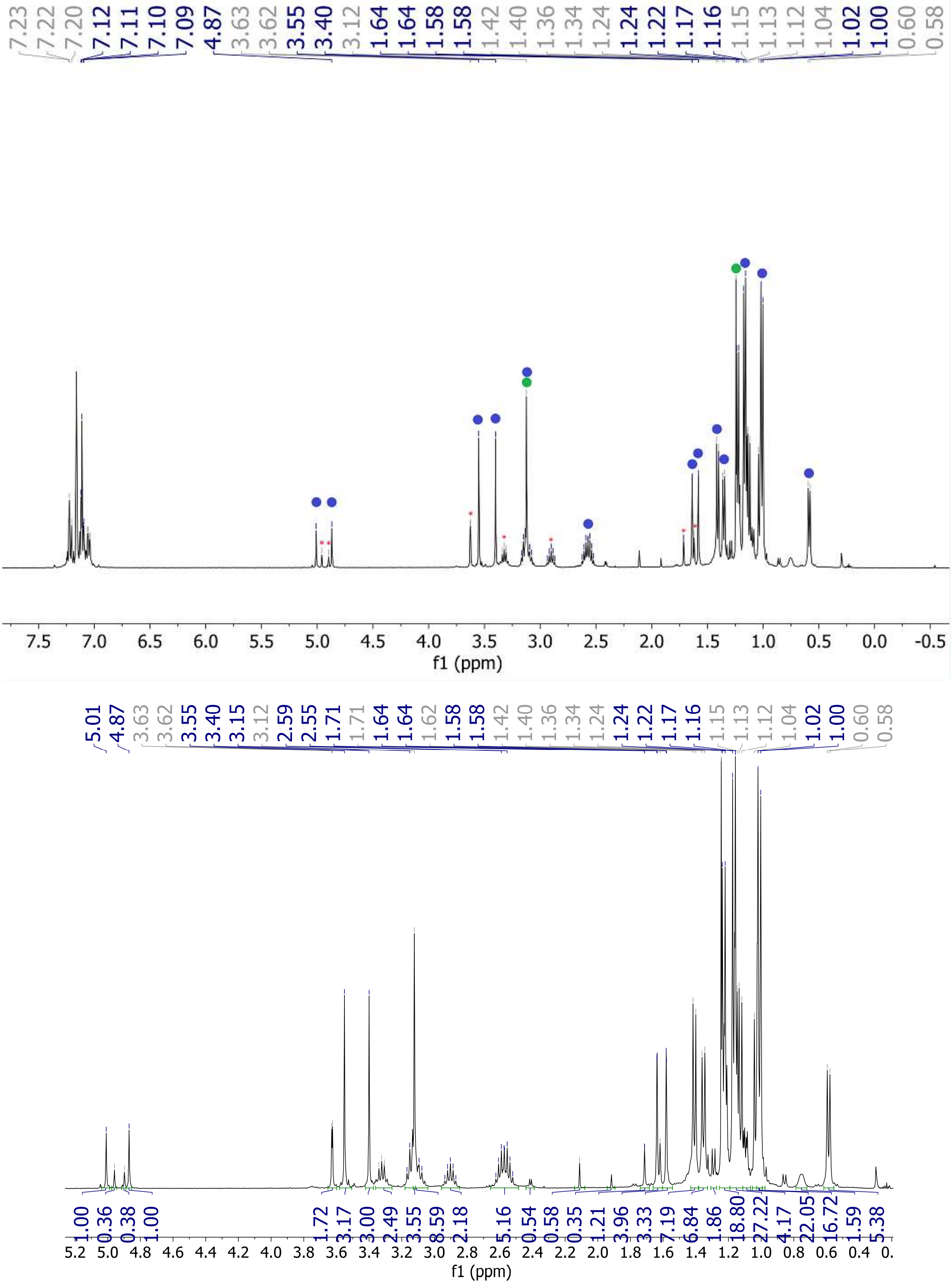

Figure S17. ^{1}H NMR spectrum (400.1 MHz, C_6D_6, 294 K) of *in-situ* generated [{(^{iPrDip}NacNac)Mg(^{Me}NHC)}(μ-S){Mg(^{iPrDip}NacNac)}] **6a** from the reaction of [{(^{iPrDip}NacNac)Mg}$_2$] **1** with ^{Me}NHC=S. Resonances labelled with a blue dot belong to **6a**, those

labelled with a green dot belong to unreacted ^{Me}NHC=S, while those labelled with a red asterisk belong to a currently unknown impurity (possibly [{(^{iPrDip}NacNac)Mg(^{Me}NHC=S)}(μ-S){Mg(^{iPrDip}NacNac)}]).

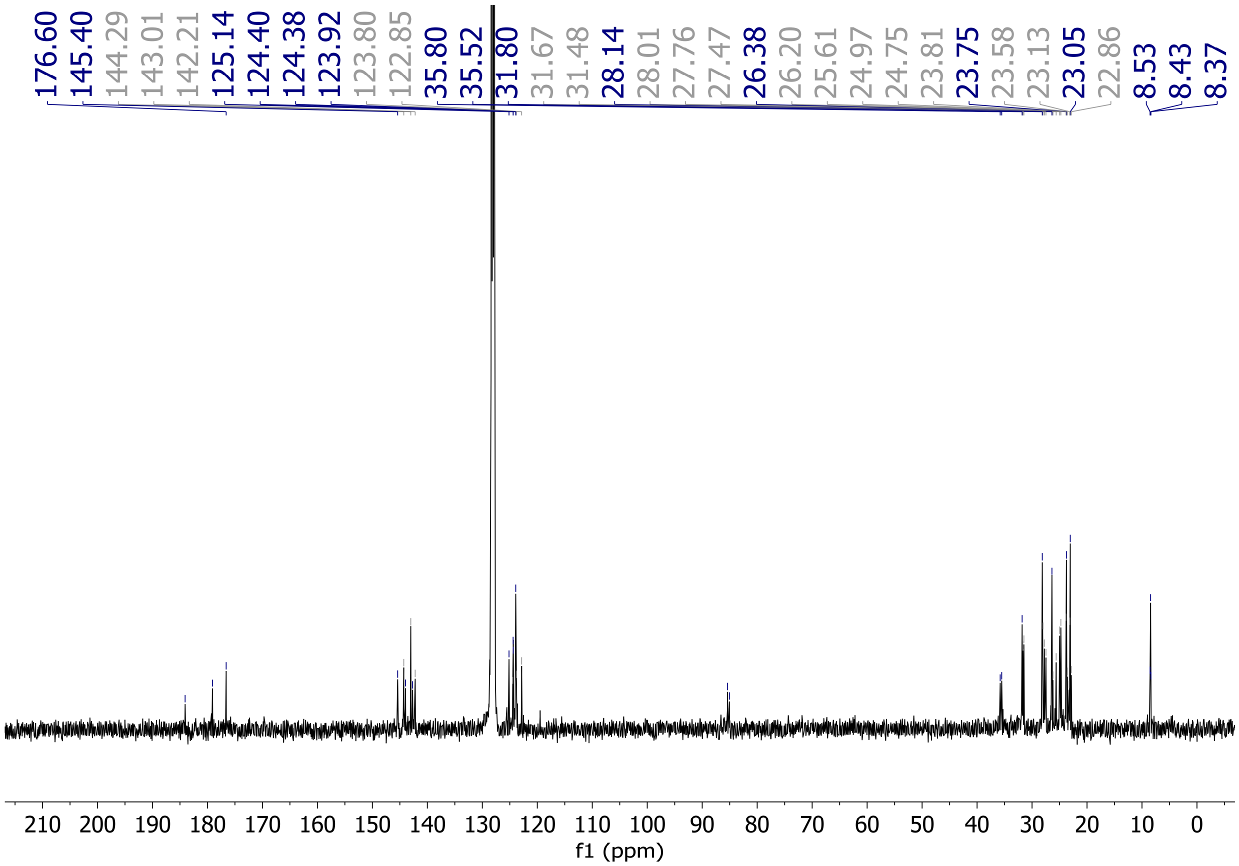

Figure S18. $^{13}C\{^1H\}$ NMR spectrum (125.7 MHz, C_6D_6, 294 K) of **6a**.

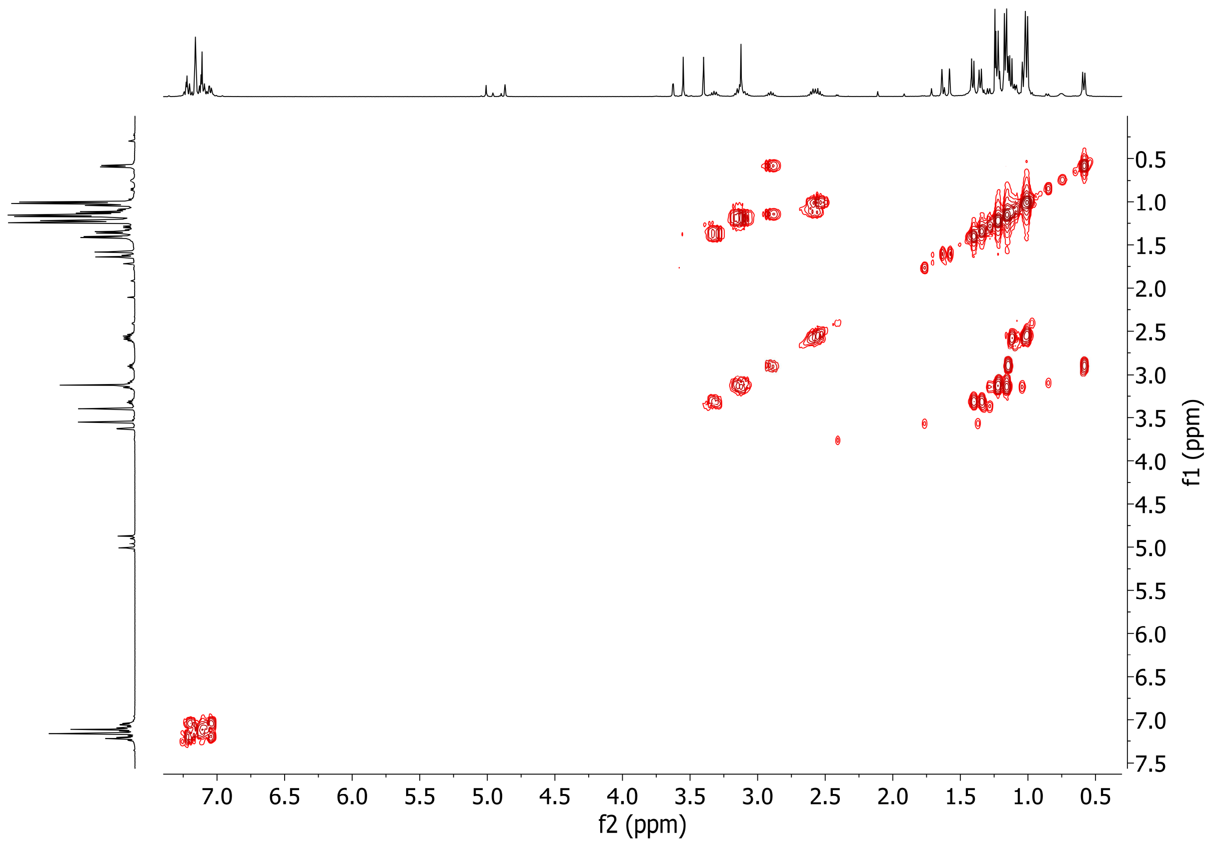

Figure S19. ^{1}H-^{1}H COSY NMR spectrum of **6a**.

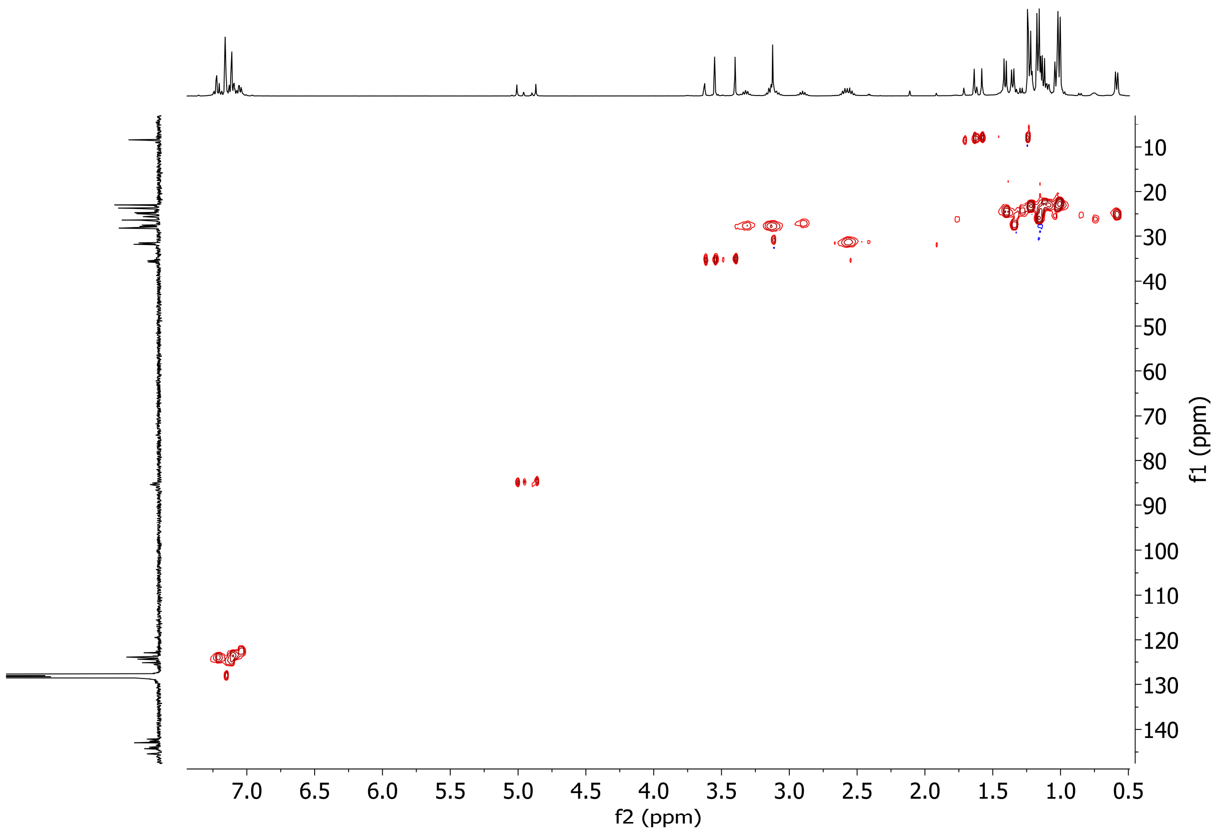

Figure S20. ^{1}H-^{13}C HSQC NMR spectrum of **6a**.

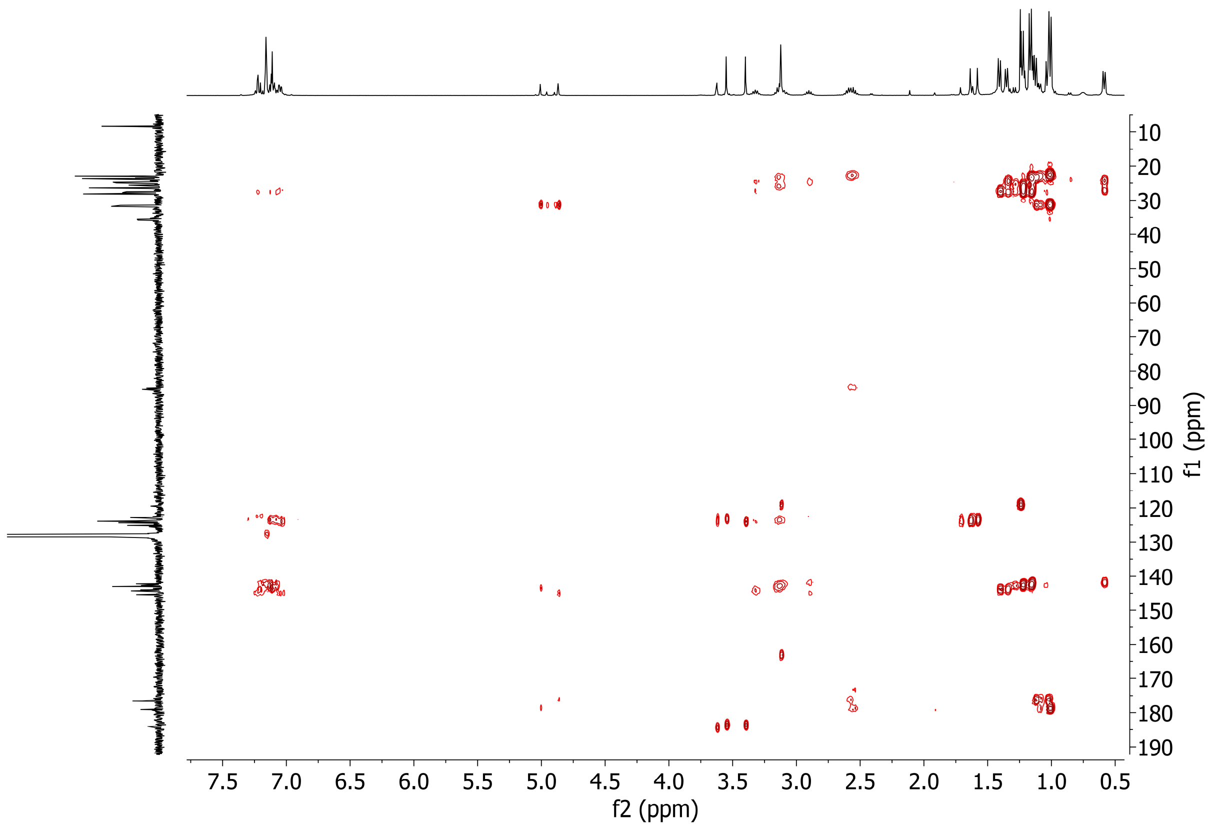

Figure S21. ^{1}H-^{13}C HMBC NMR spectrum of **6a**.

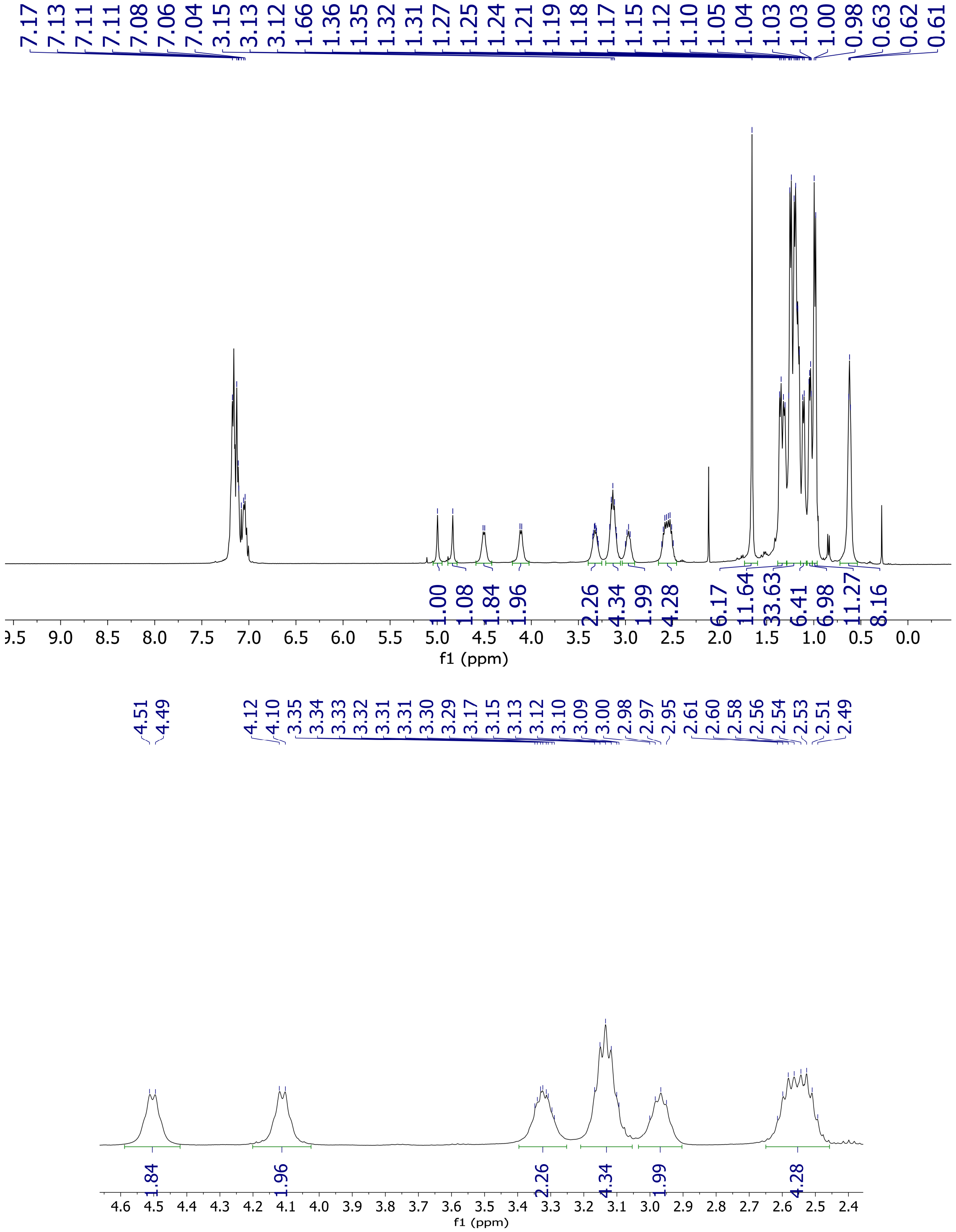

Figure S22. ^{1}H NMR spectrum (400.1 MHz, C_6D_6, 294 K) of *in-situ* generated [{(^{iPrDip}NacNac)Mg(^{Et}NHC)}(μ-S){Mg(^{iPrDip}NacNac)}] **6b** from the reaction of [{(^{iPrDip}NacNac)Mg}$_2$] **1** with ^{Et}NHC=S.

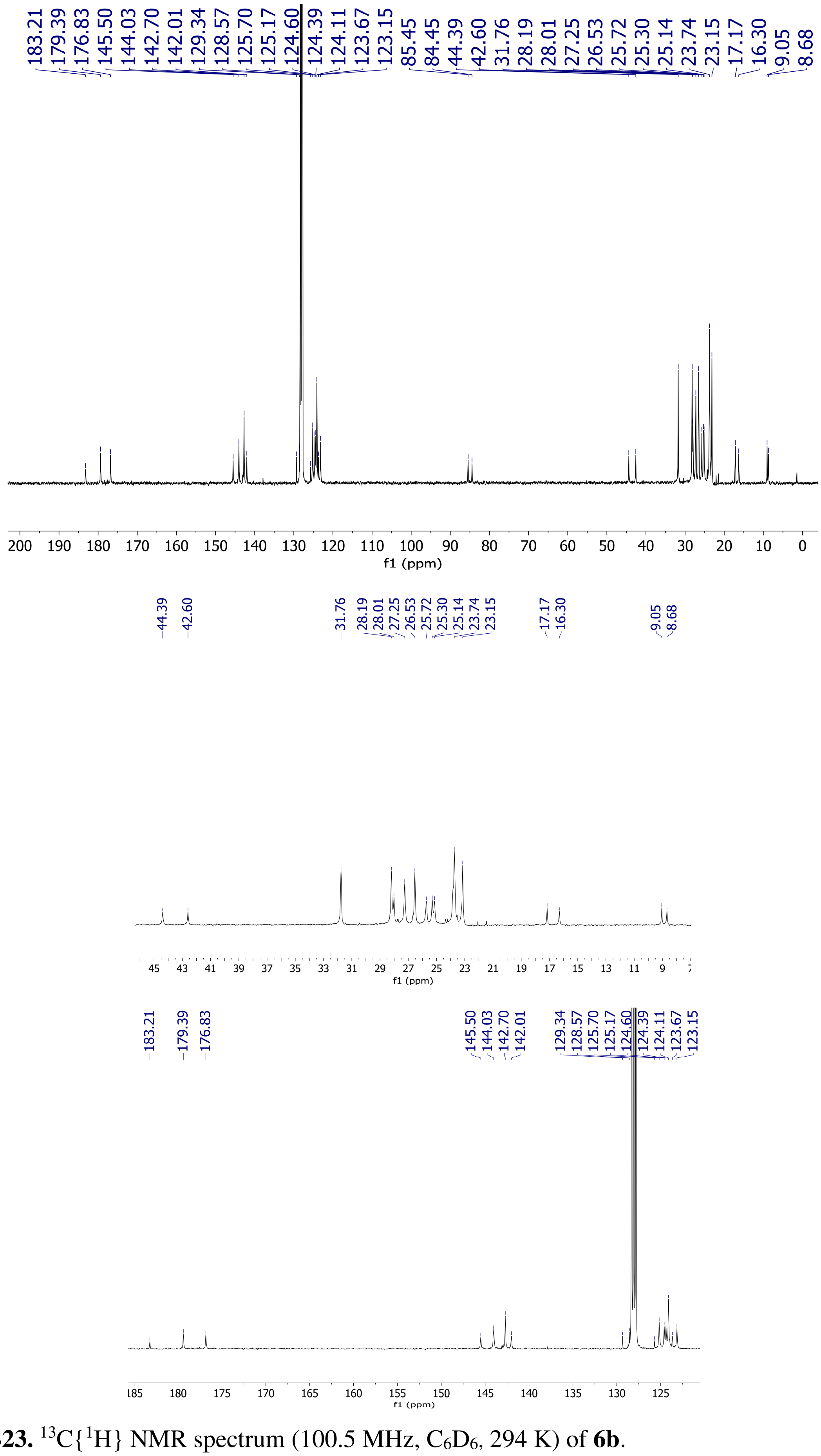

Figure S23. $^{13}C\{^{1}H\}$ NMR spectrum (100.5 MHz, C_6D_6, 294 K) of **6b**.

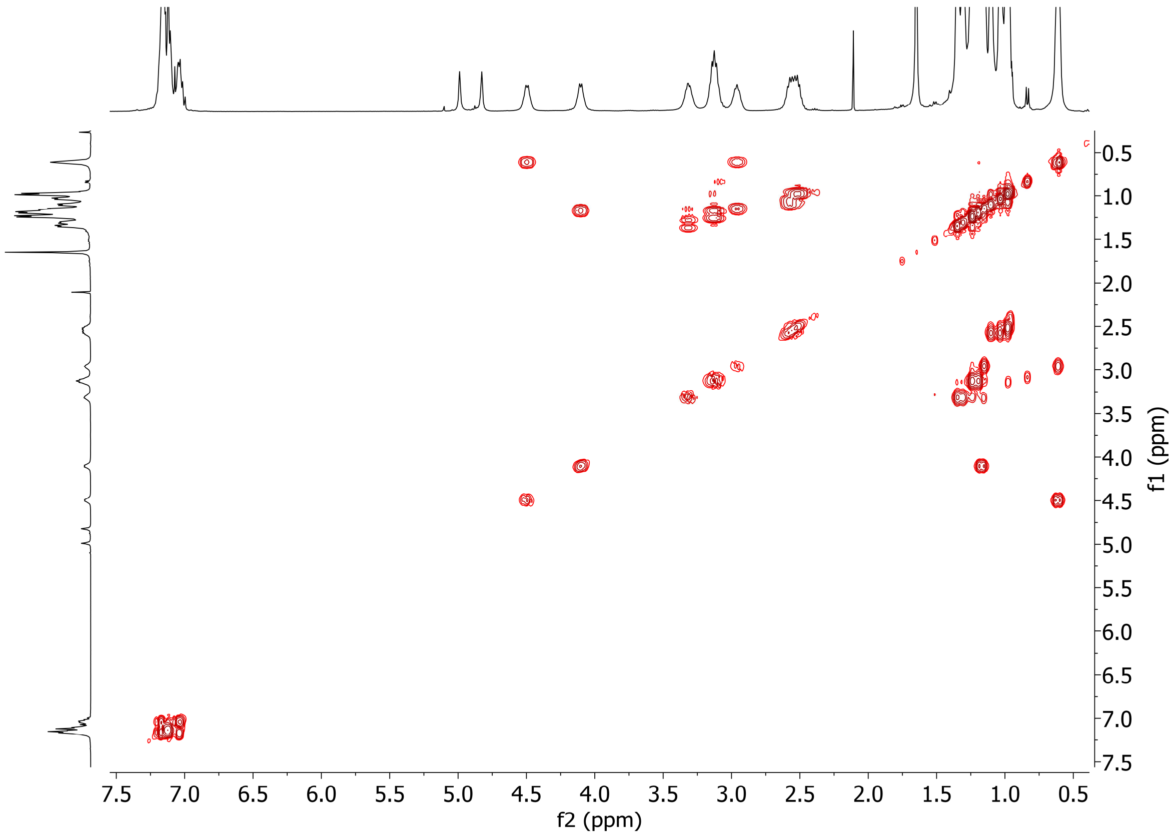

Figure S24. ^{1}H-^{1}H COSY NMR spectrum of **6b**.

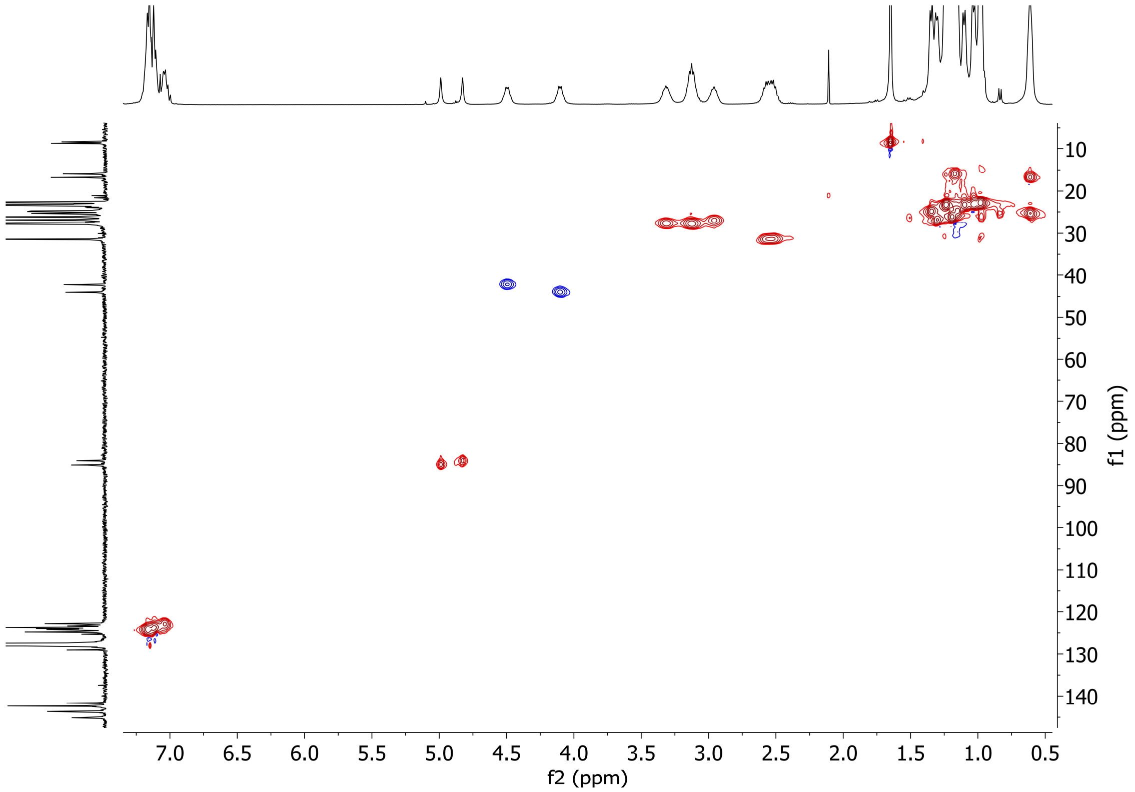

Figure S25. ^{1}H-^{13}C HSQC NMR spectrum of **6b**.

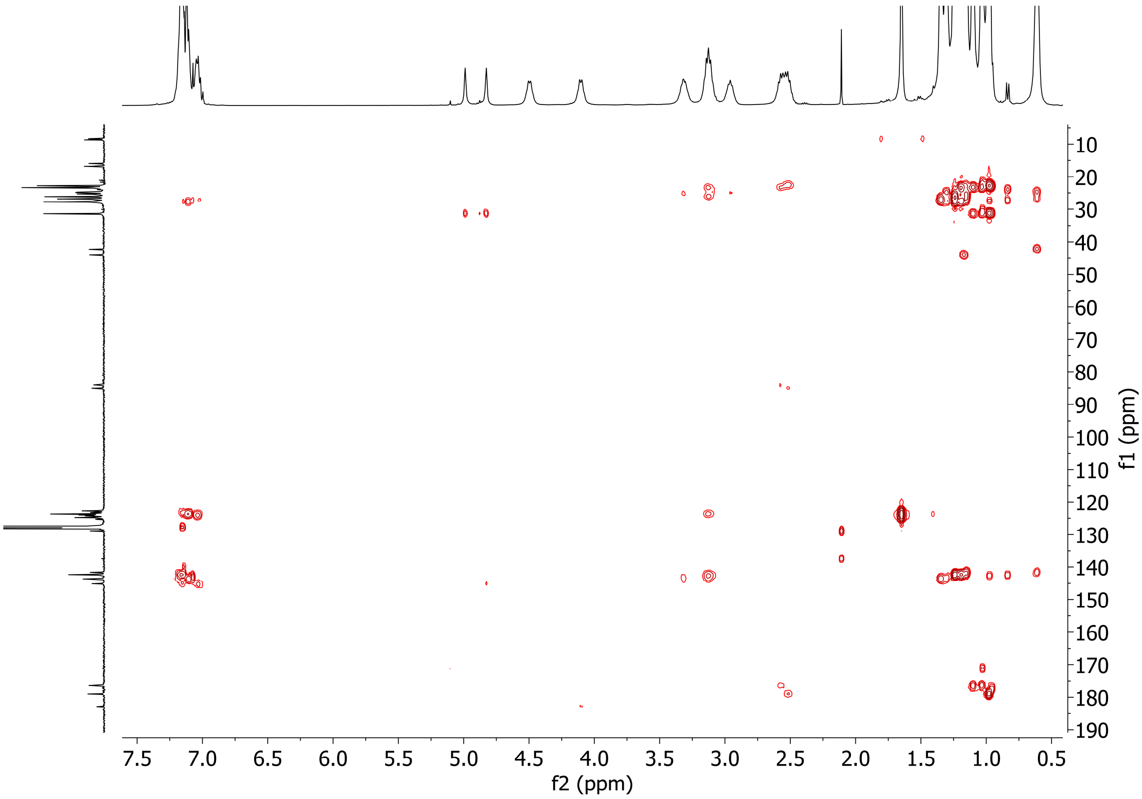

Figure S26. ^{1}H-^{13}C HMBC NMR spectrum of **6b**.

.

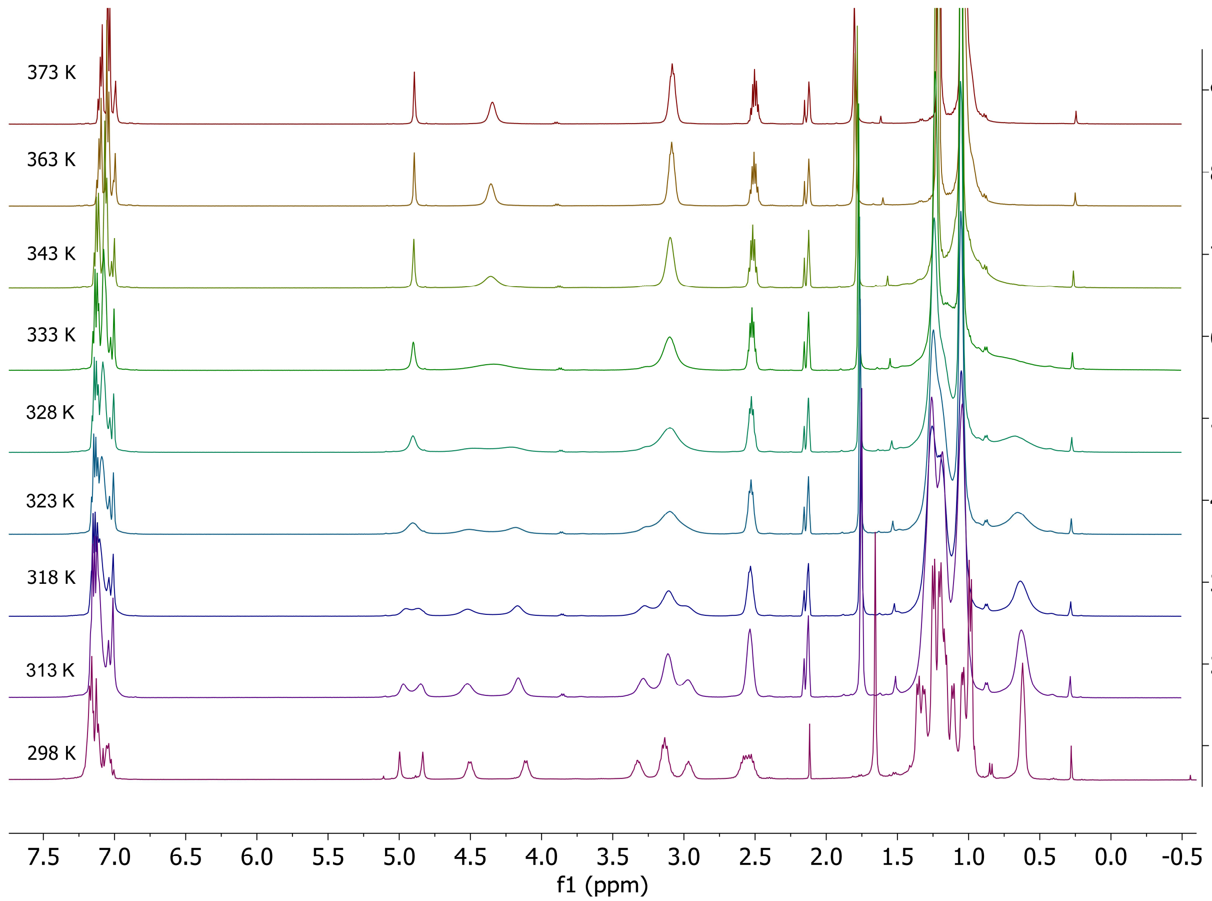

Figure S27. VT NMR spectrum (499.9 MHz, C_7D_8) of **6b**.

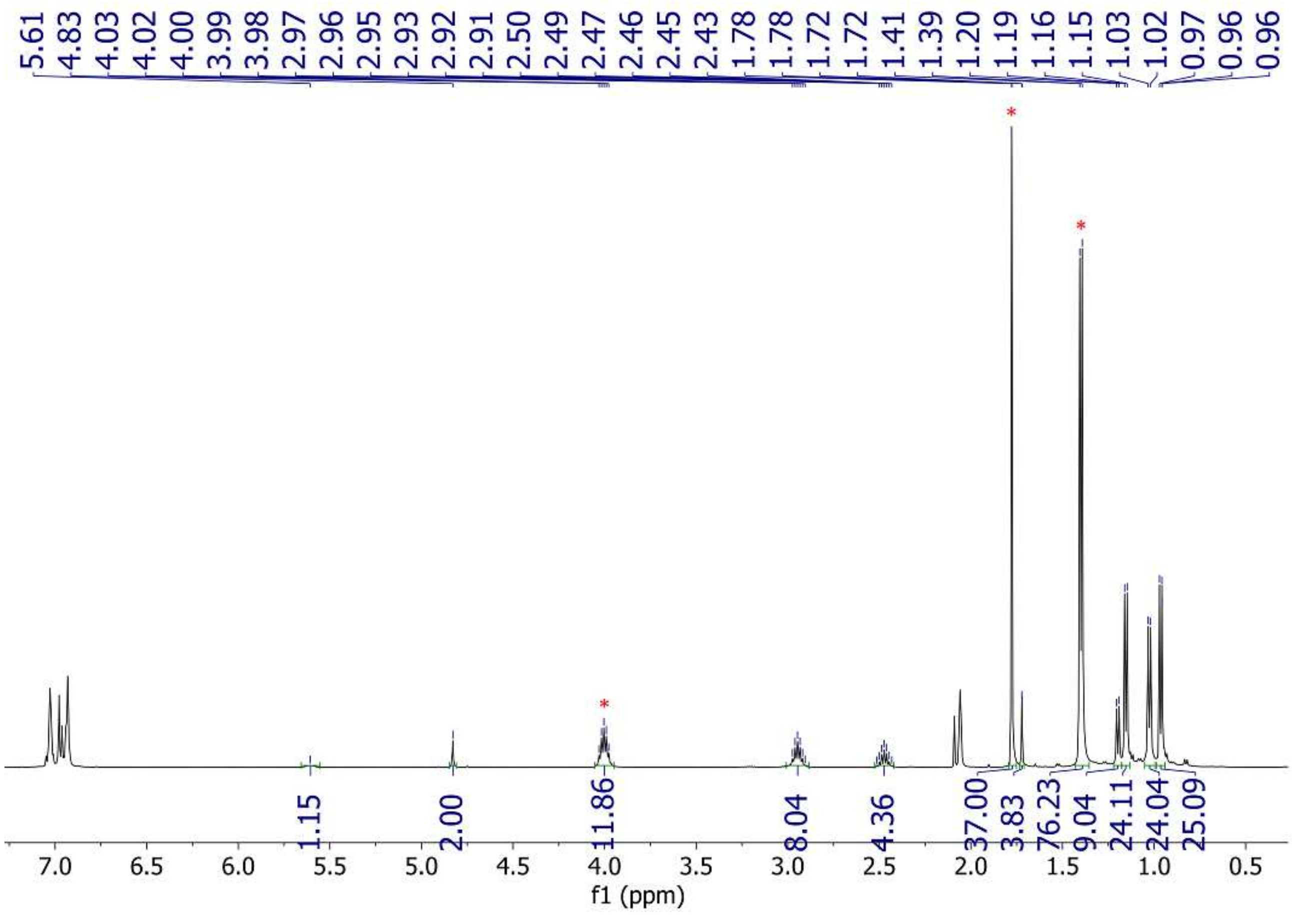

Figure S28. ^{1}H NMR spectrum (499.9 MHz, C_6D_6, 373 K) of *in-situ* generated [{(^{iPrDip}NacNac)Mg(^{iPr}NHC)}(μ-S){Mg(^{iPrDip}NacNac)}] **6c** from the reaction of [{(^{iPrDip}NacNac)Mg}$_2$] **1** with ^{iPr}NHC=S. Resonances labelled with a red asterisk belong to excess ^{iPr}NHC=S.

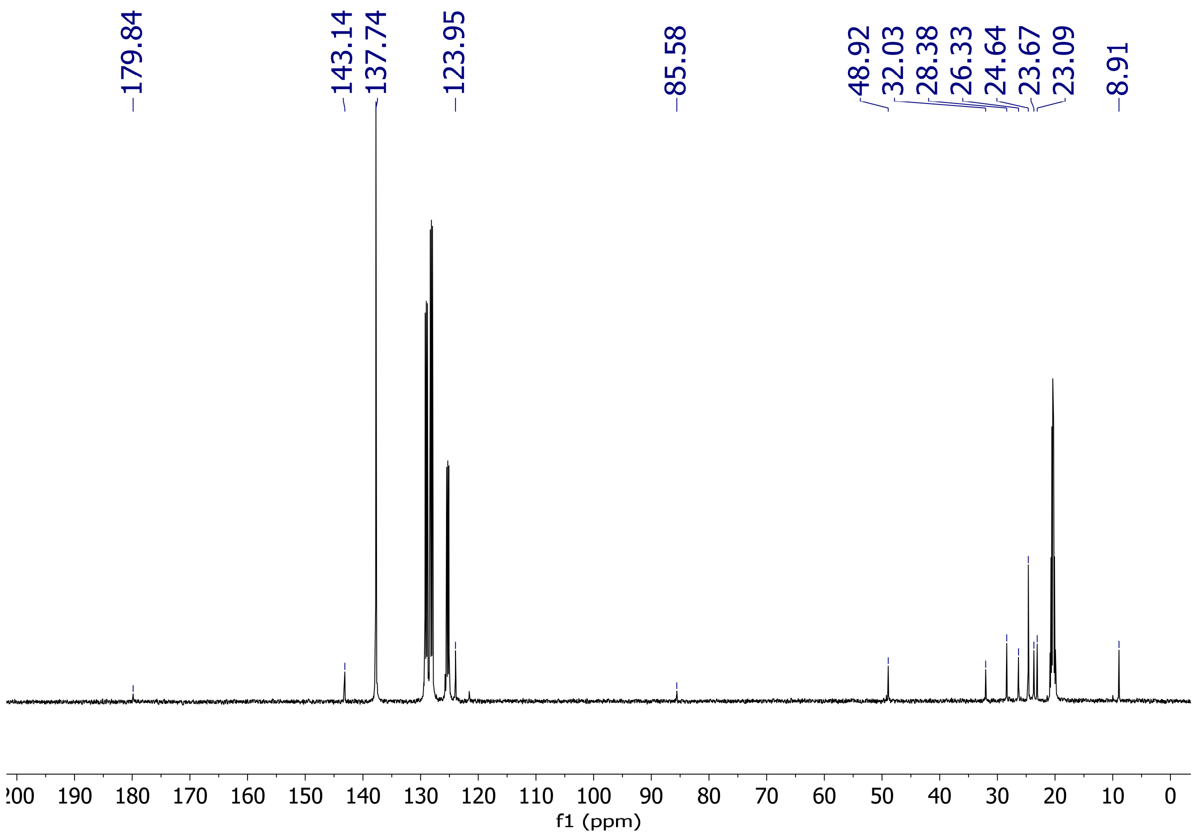

Figure S29. $^{13}C\{^{1}H\}$ NMR spectrum (125.7 MHz, C_7D_8, 373 K) of **6c**.

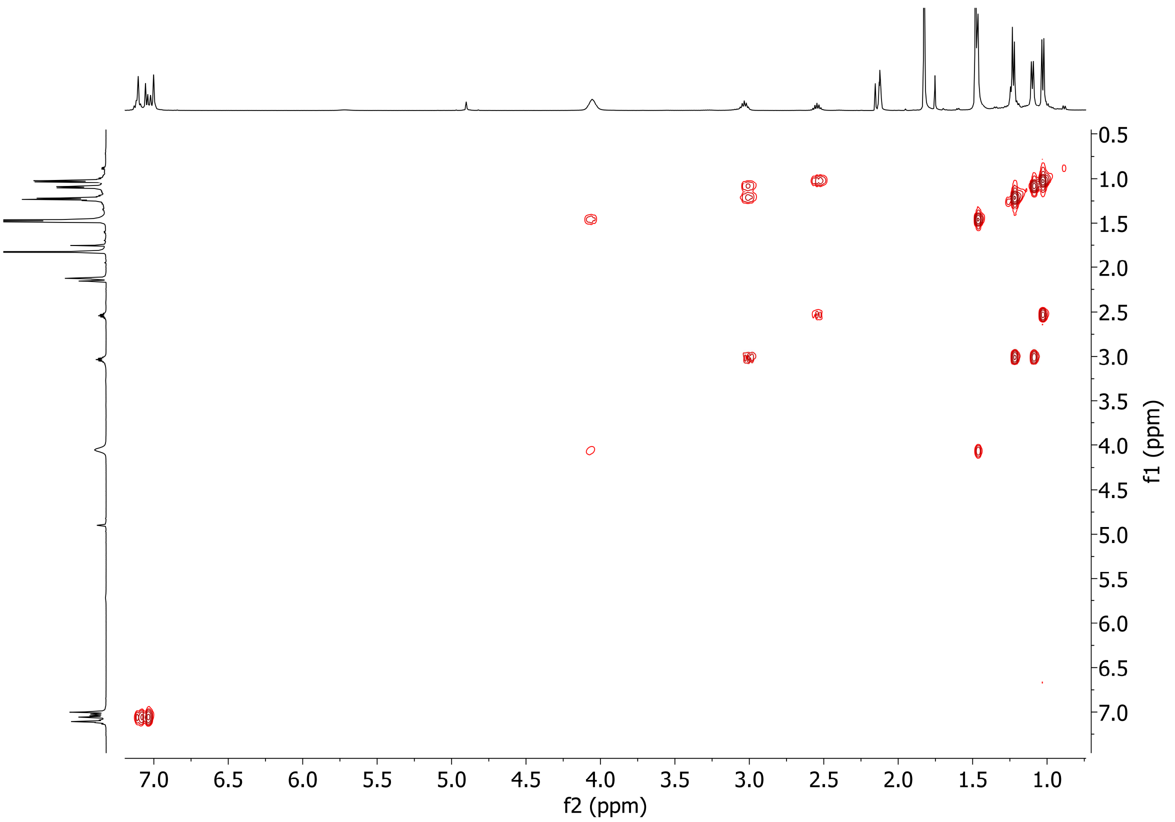

Figure S30. ^{1}H-^{1}H COSY NMR spectrum of **6c**.

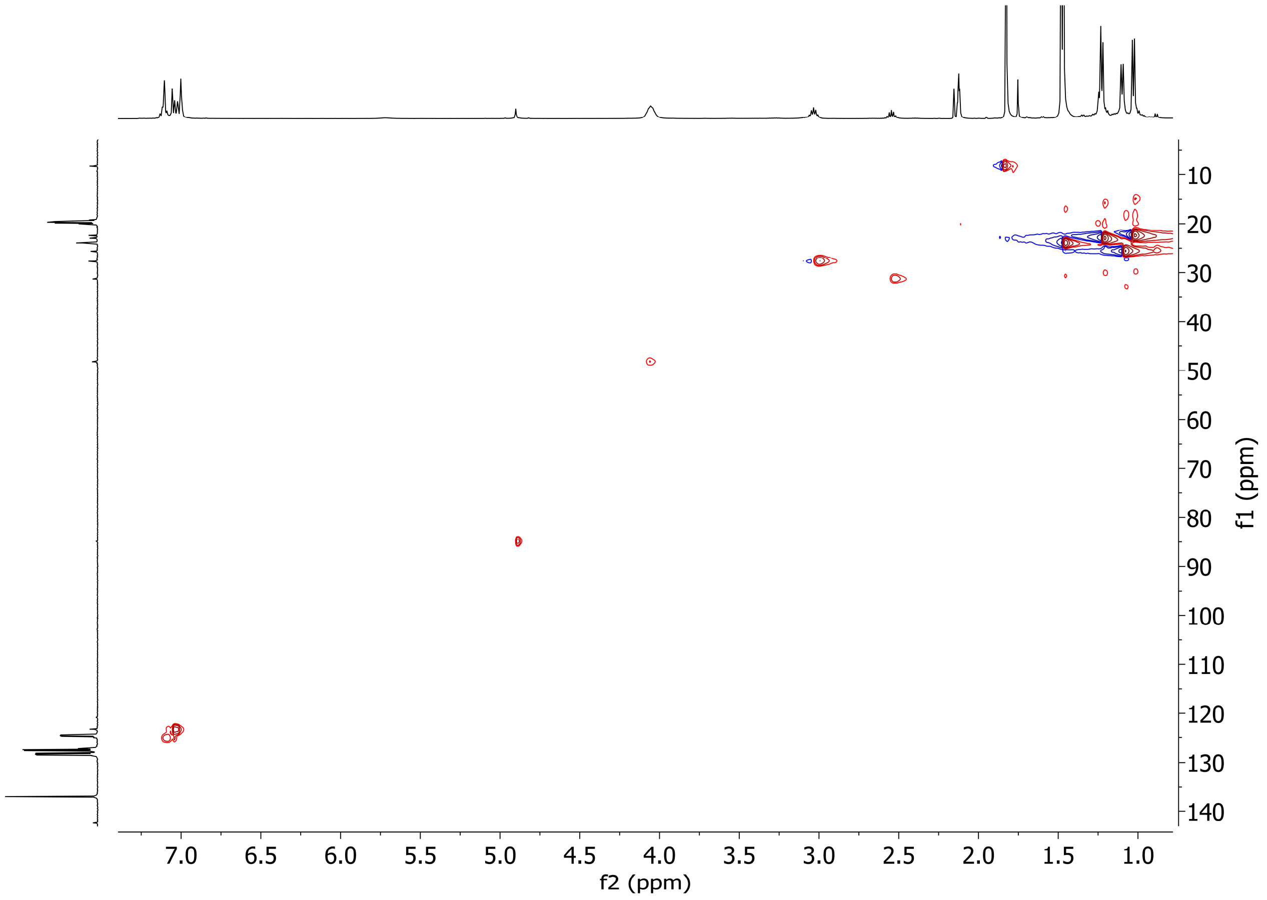

Figure S31. ^{1}H-^{13}C HSQC NMR spectrum of **6c**.

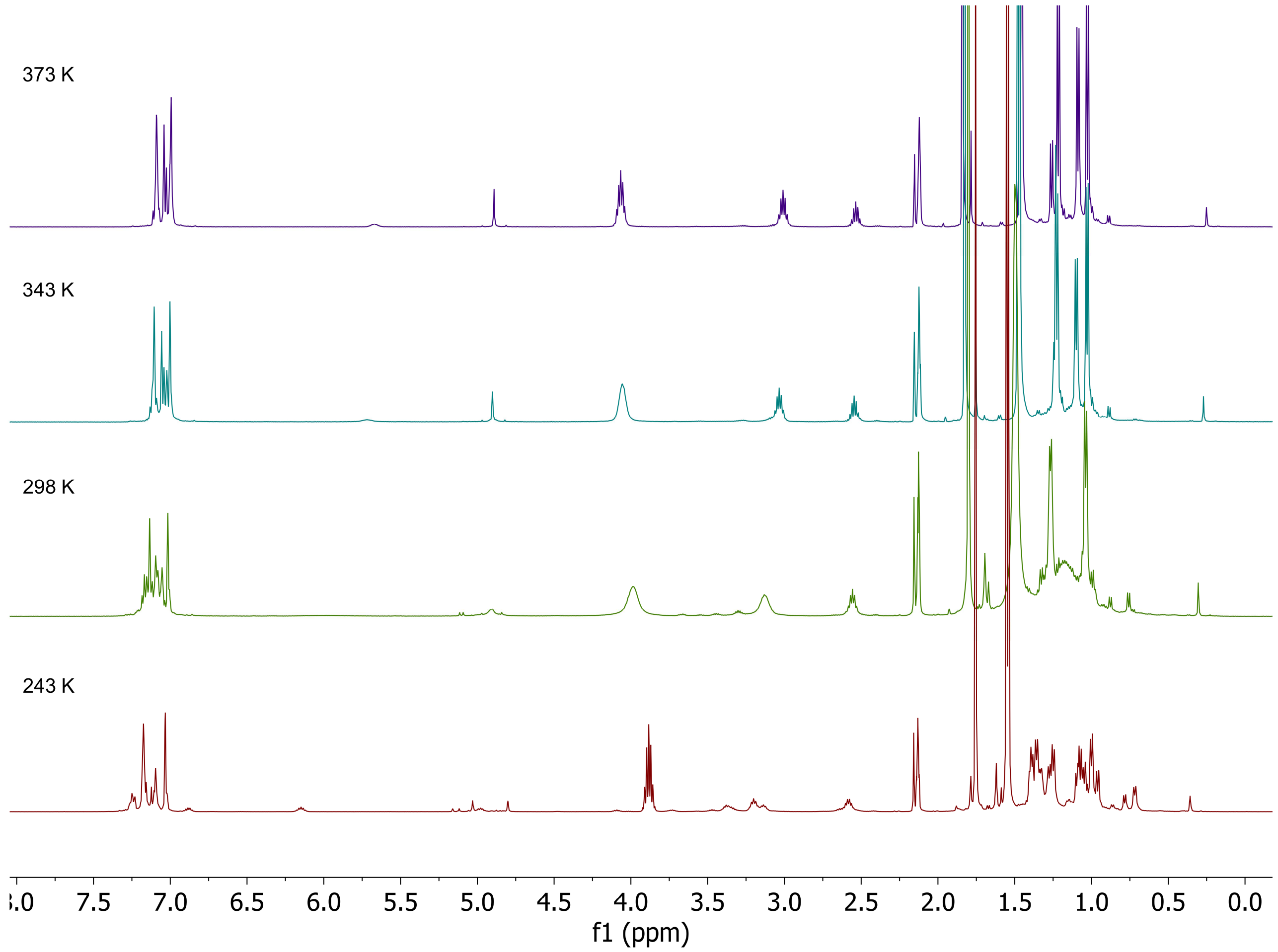

Figure S32. VT NMR spectrum (499.9 MHz, C_7D_8) of **6c**.

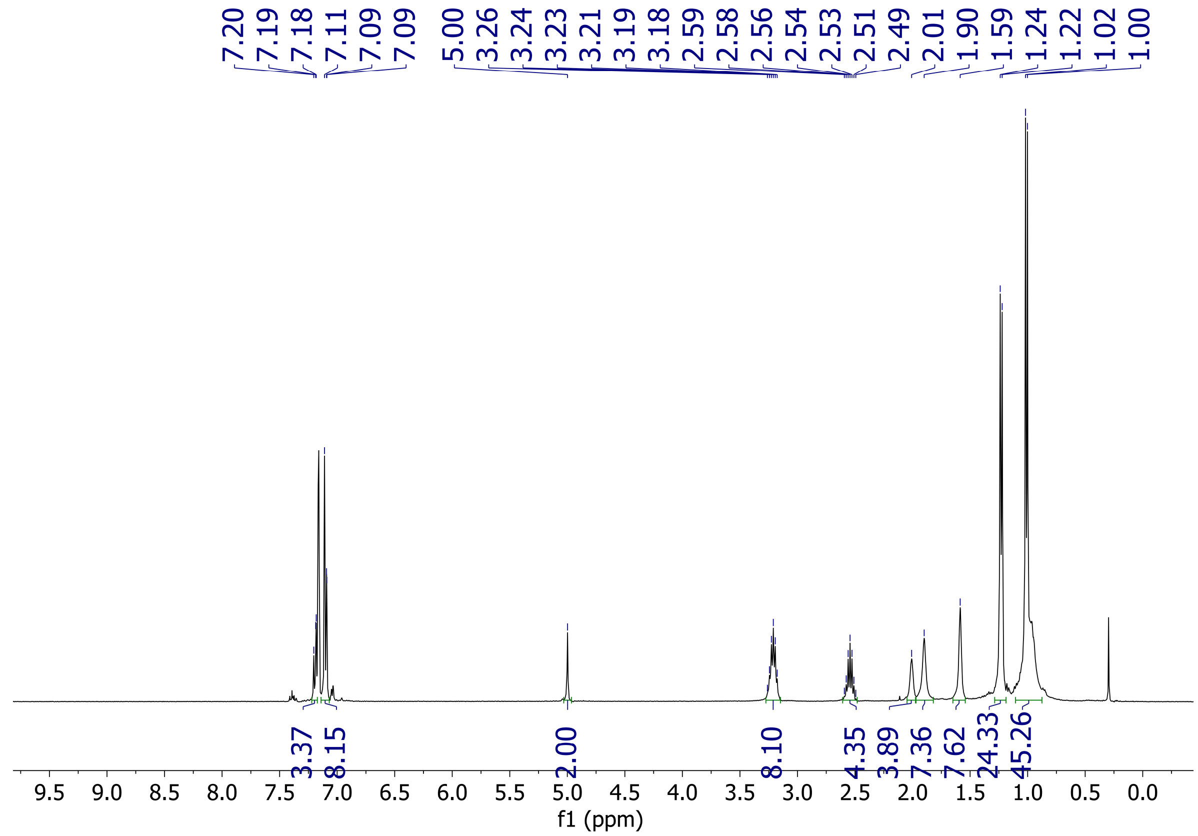

Figure S33. ^{1}H NMR spectrum (400.1 MHz, C_6D_6, 294 K) of [{(^{iPrDip}NacNac)Mg}$_2$(μ-SN_3Ad)] **7**.

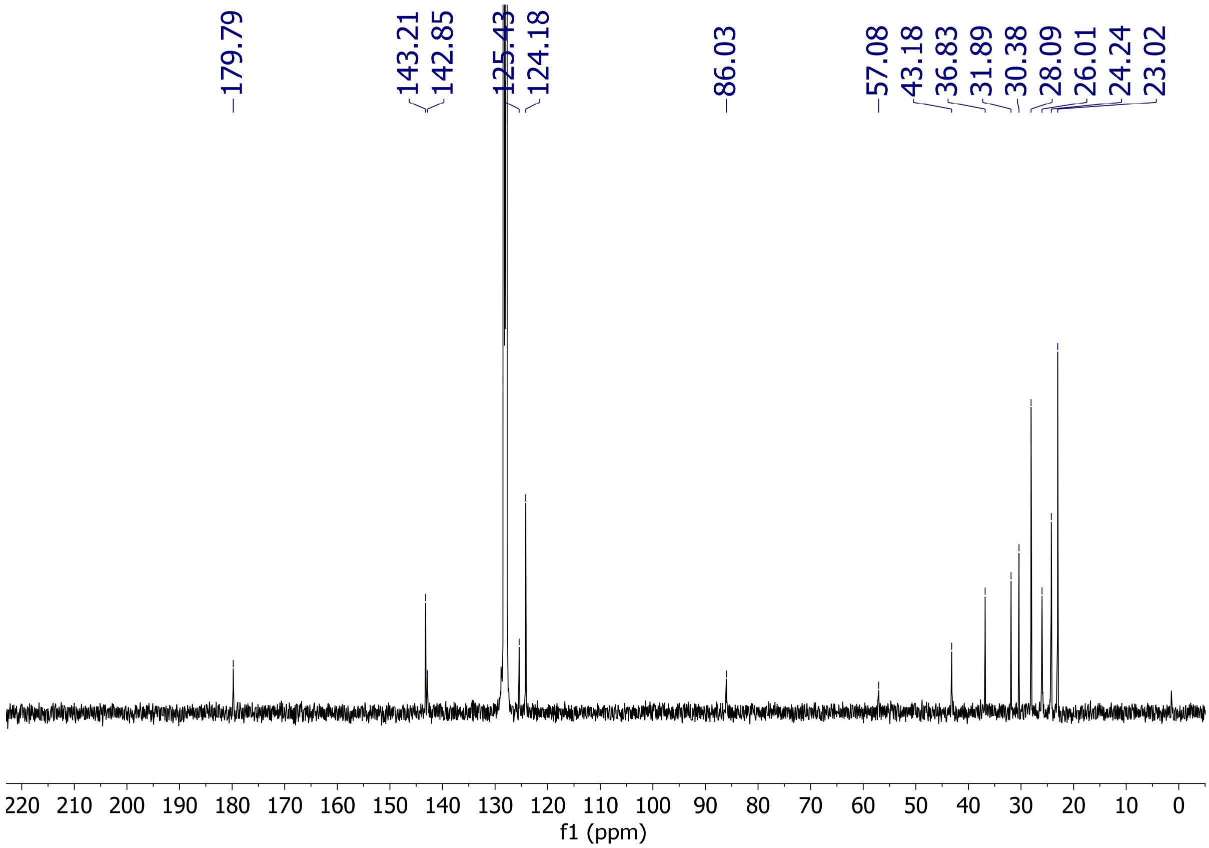

Figure S34. ^{13}C{^{1}H} NMR spectrum (100.5 MHz, C_6D_6, 294 K) of **7**.

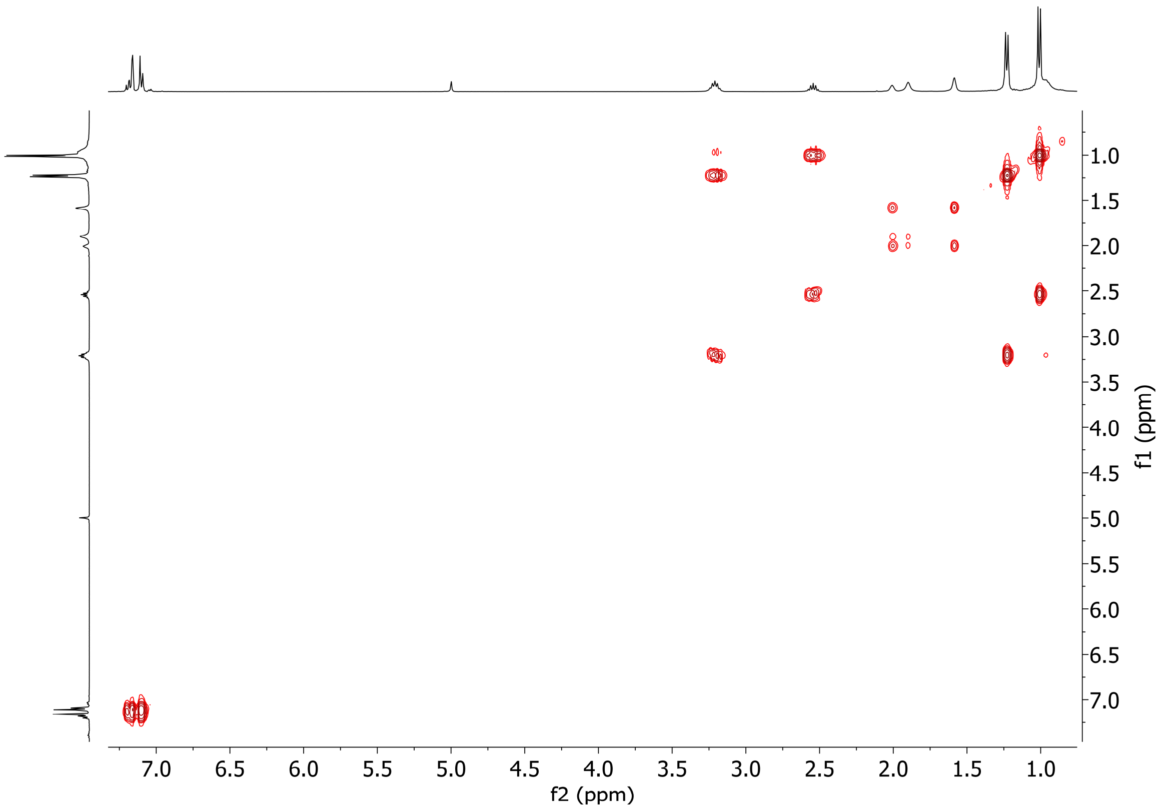

Figure S35. ^{1}H-^{1}H COSY NMR spectrum of **7**.

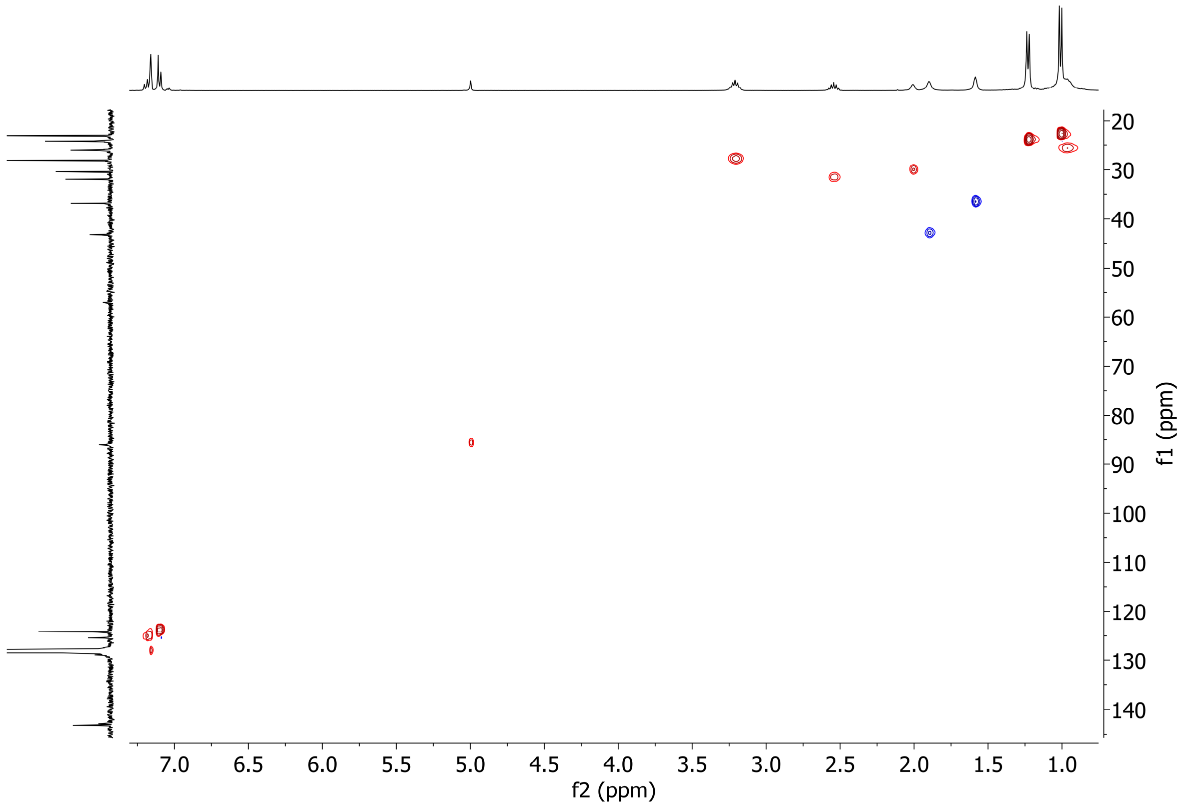

Figure S36. ^{1}H-^{13}C HSQC NMR spectrum of **7**.

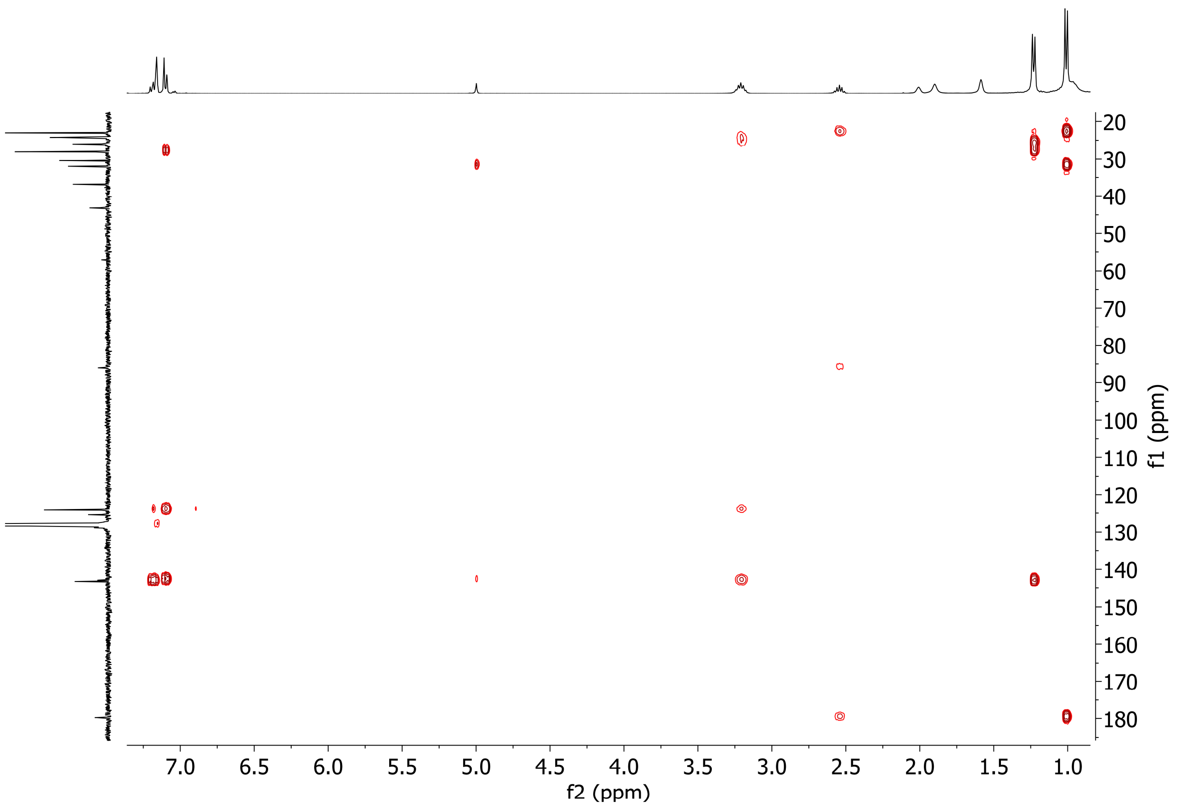

Figure S37. ^{1}H-^{13}C HMBC NMR spectrum of **7**.

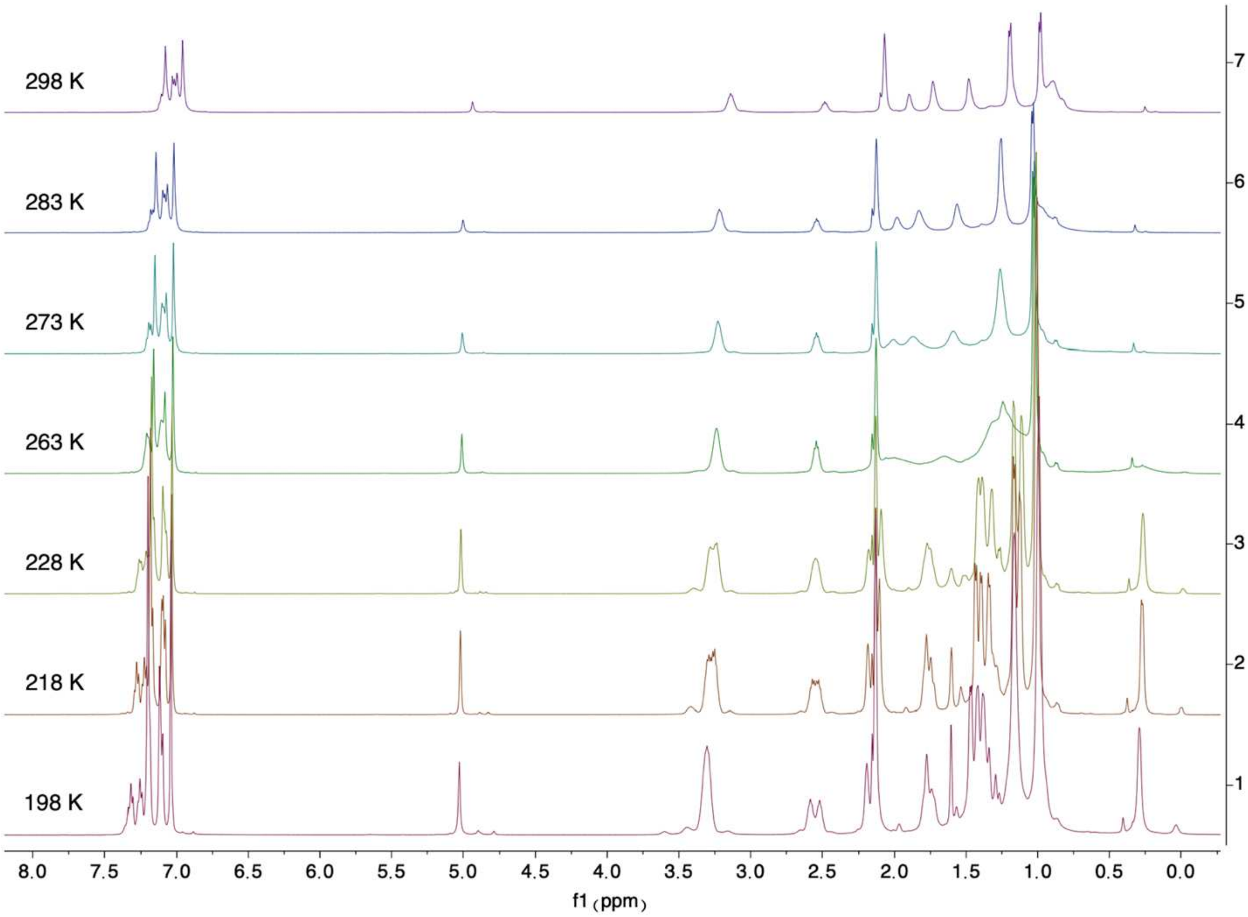

Figure S38. VT NMR spectrum (499.9 MHz, C_7D_8) of **7**.

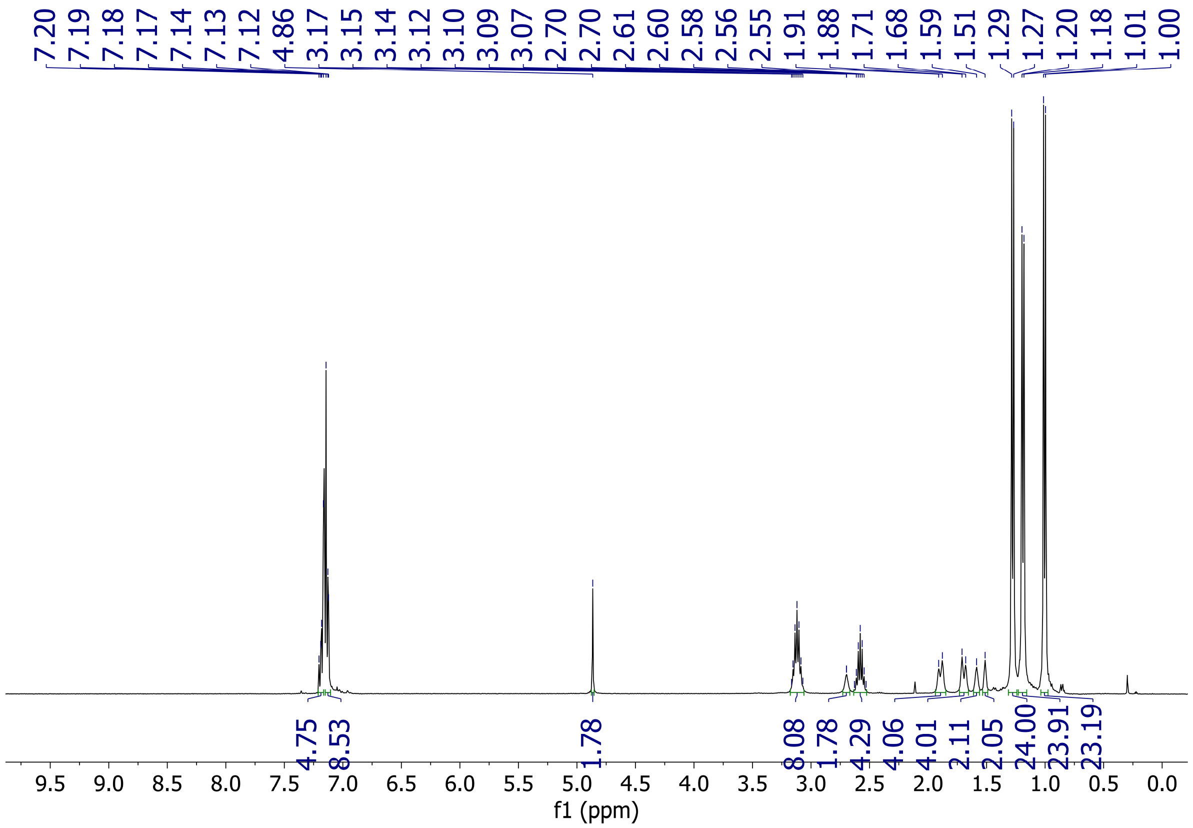

Figure S39. ^{1}H NMR spectrum (400.1 MHz, C_6D_6, 294 K) of *in-situ* generated [(^{iPrDip}NacNac)Mg(OAd)}$_2$(μ-S)] **8a** from the reaction of [{(^{iPrDip}NacNac)Mg}$_2$(μ-S)] **4** with two equivalents of 2-adamantanone (OAd).

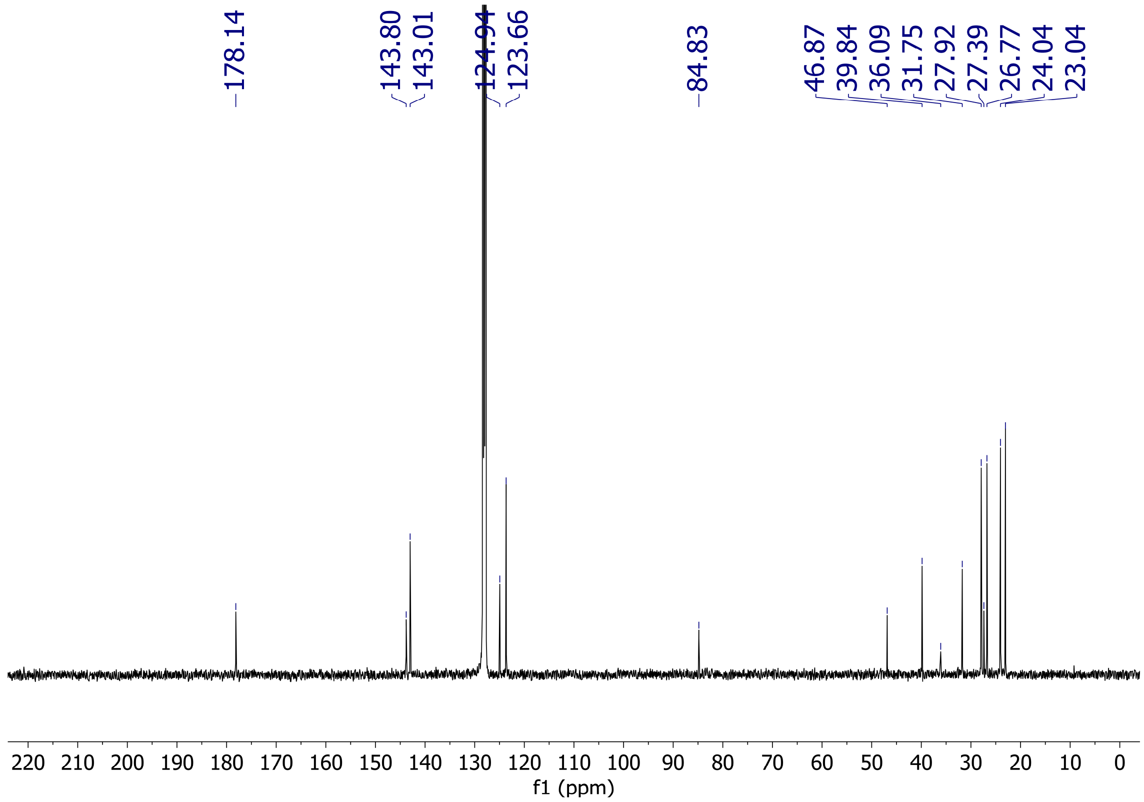

Figure S40. $^{13}C\{^{1}H\}$ NMR spectrum (100.5 MHz, C_6D_6, 294 K) of **8a**.

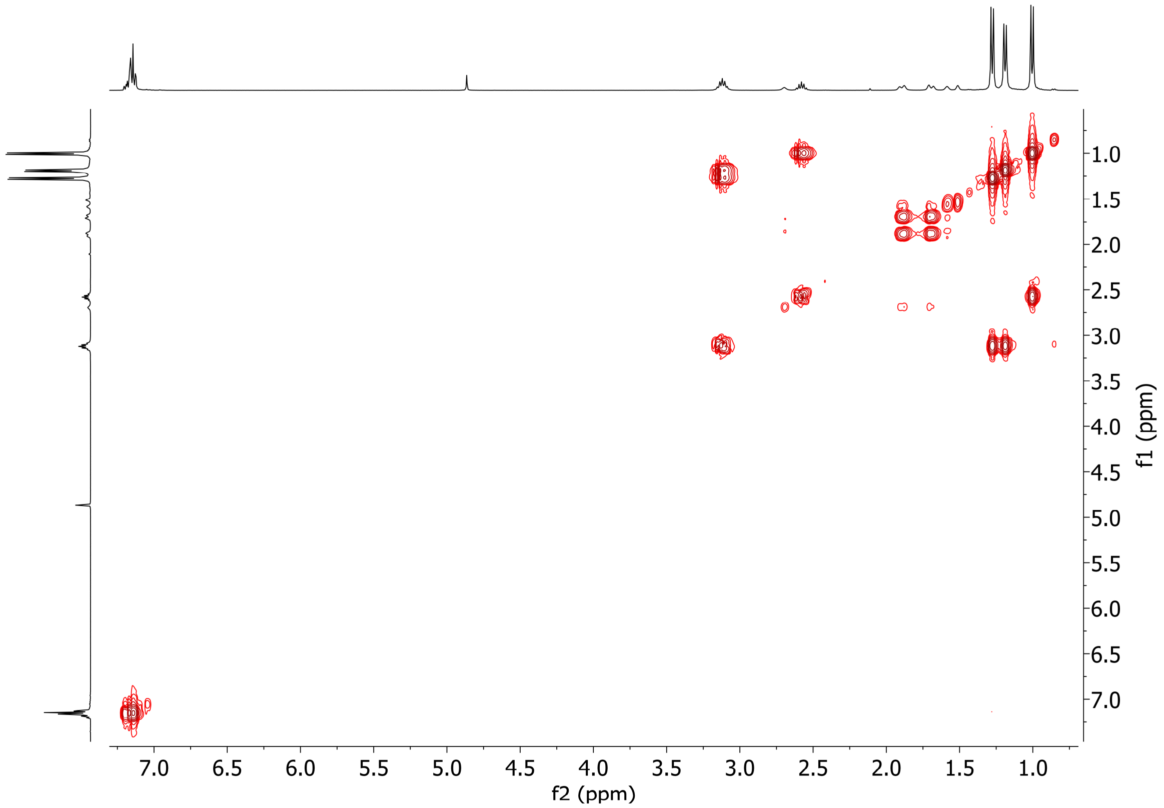

Figure S41. ^{1}H-^{1}H COSY NMR spectrum of **8a**.

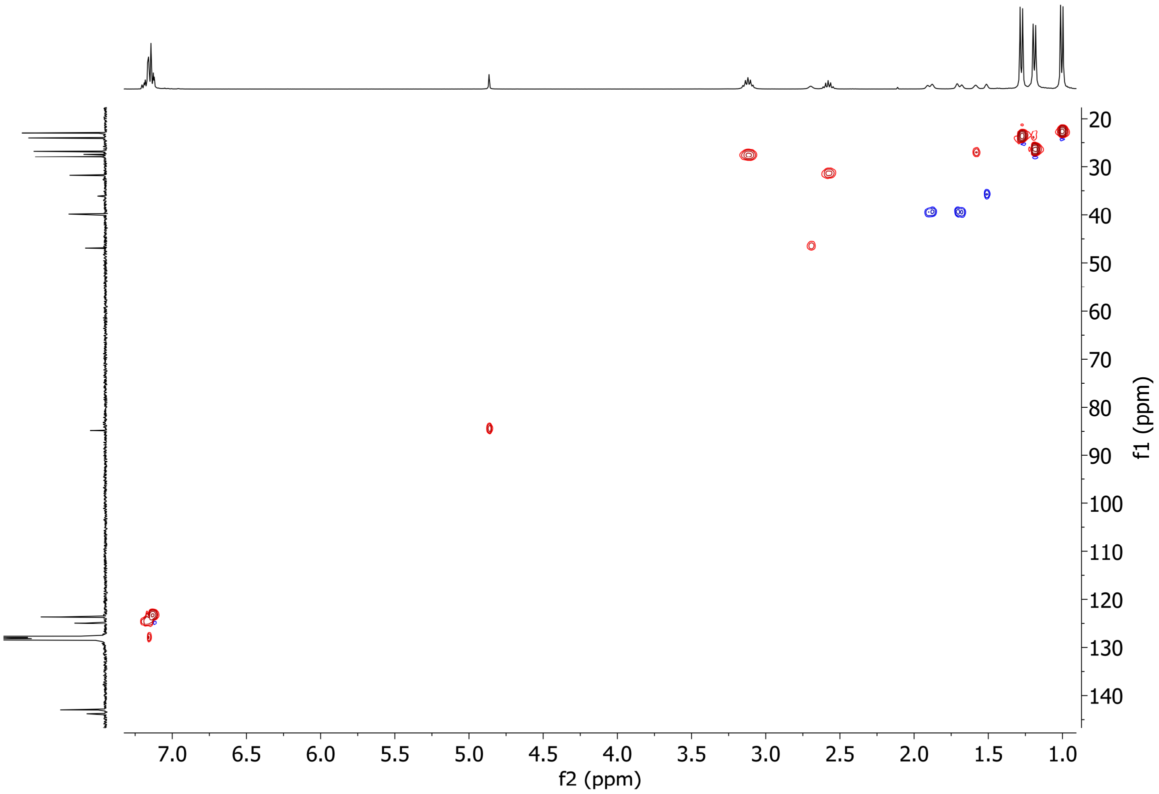

Figure S42. ^{1}H-^{13}C HSQC NMR spectrum of **8a**.

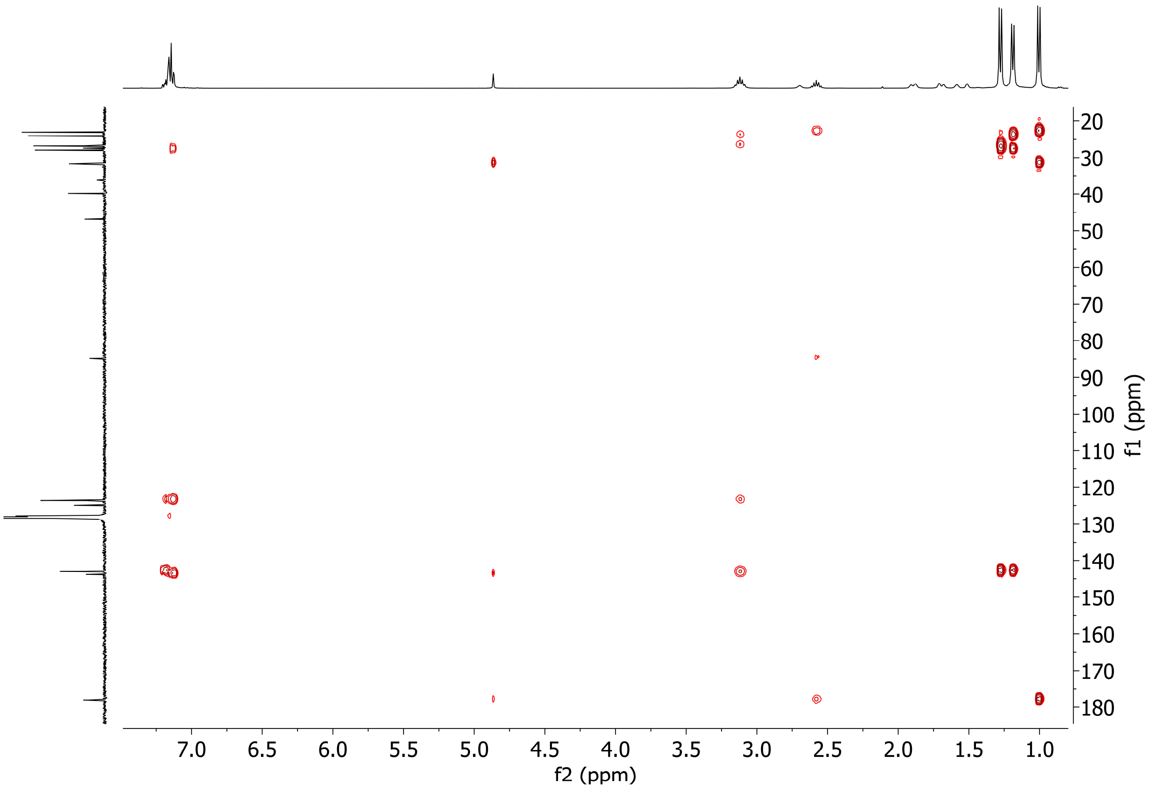

Figure S43. ^{1}H-^{13}C HMBC NMR spectrum of **8a**.

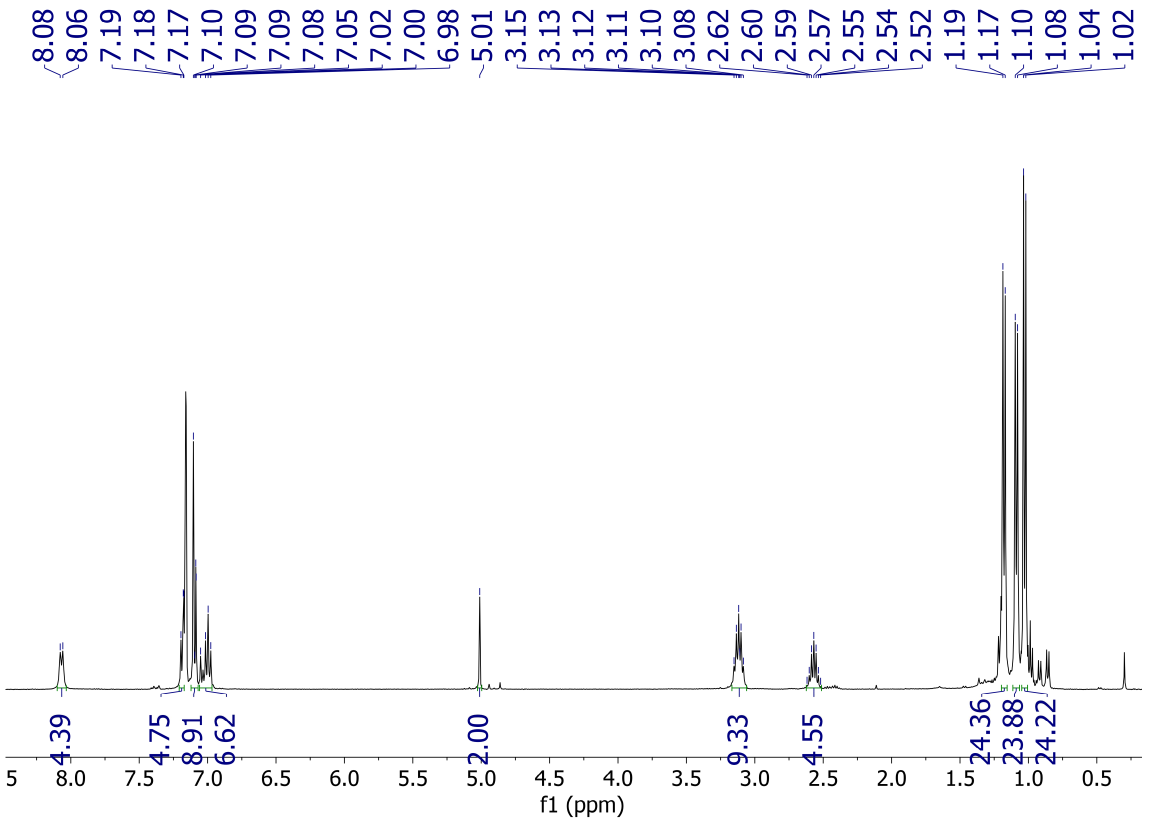

Figure S44. ^{1}H NMR spectrum (400.1 MHz, C_6D_6, 294 K) of *in-situ* generated [{(^{iPrDip}NacNac)Mg($OCPh_2$)}$_2$(μ-S)] **8b** from the reaction of [{(^{iPrDip}NacNac)Mg}$_2$(μ-S)] **4** with two equivalents of benzophenone ($OCPh_2$).

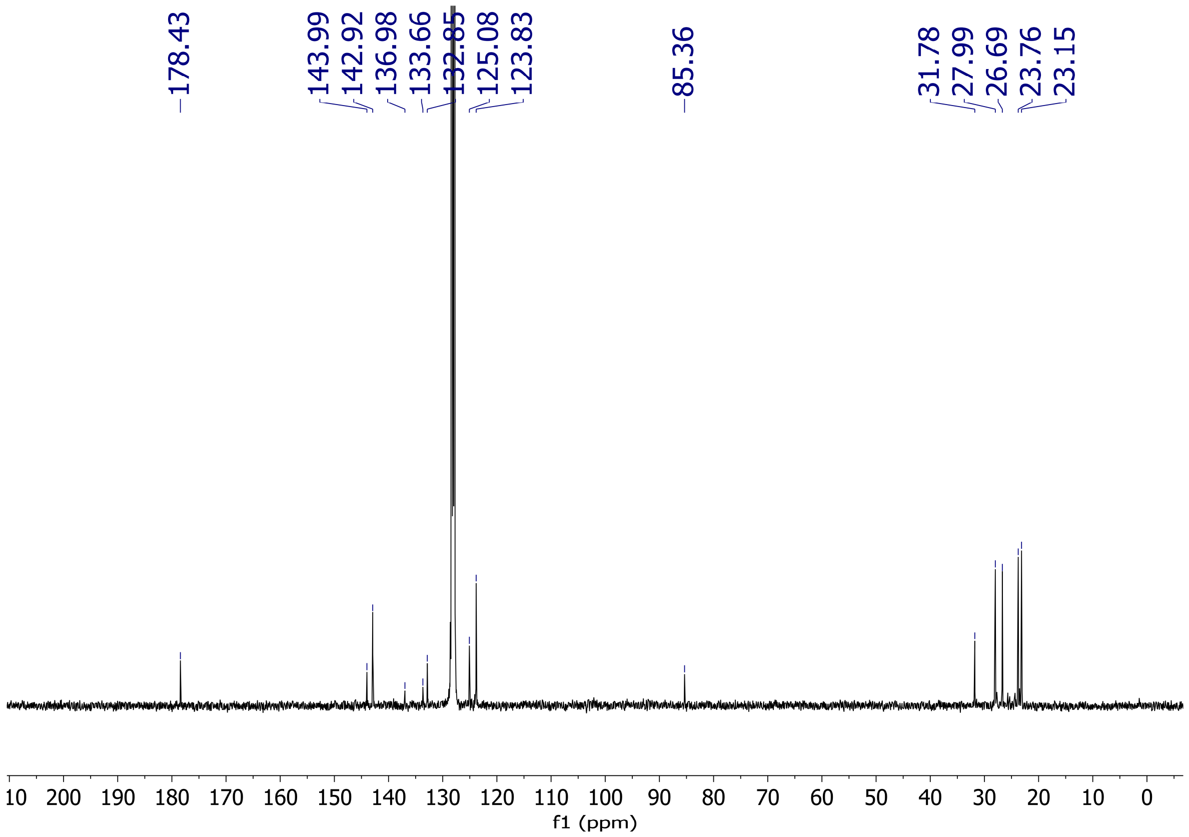

Figure S45. $^{13}C\{^{1}H\}$ NMR spectrum (100.5 MHz, C_6D_6, 294 K) of **8b**.

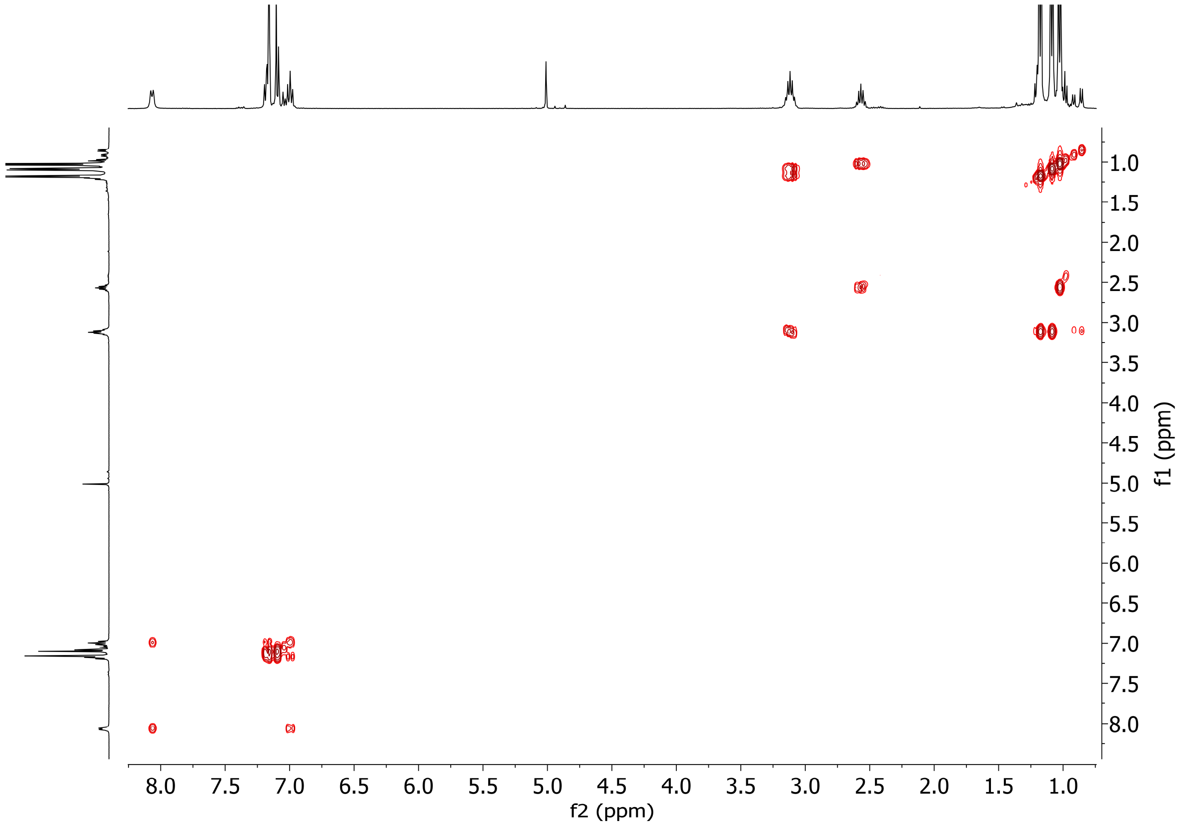

Figure S46. ^{1}H-^{1}H COSY NMR spectrum of **8b**.

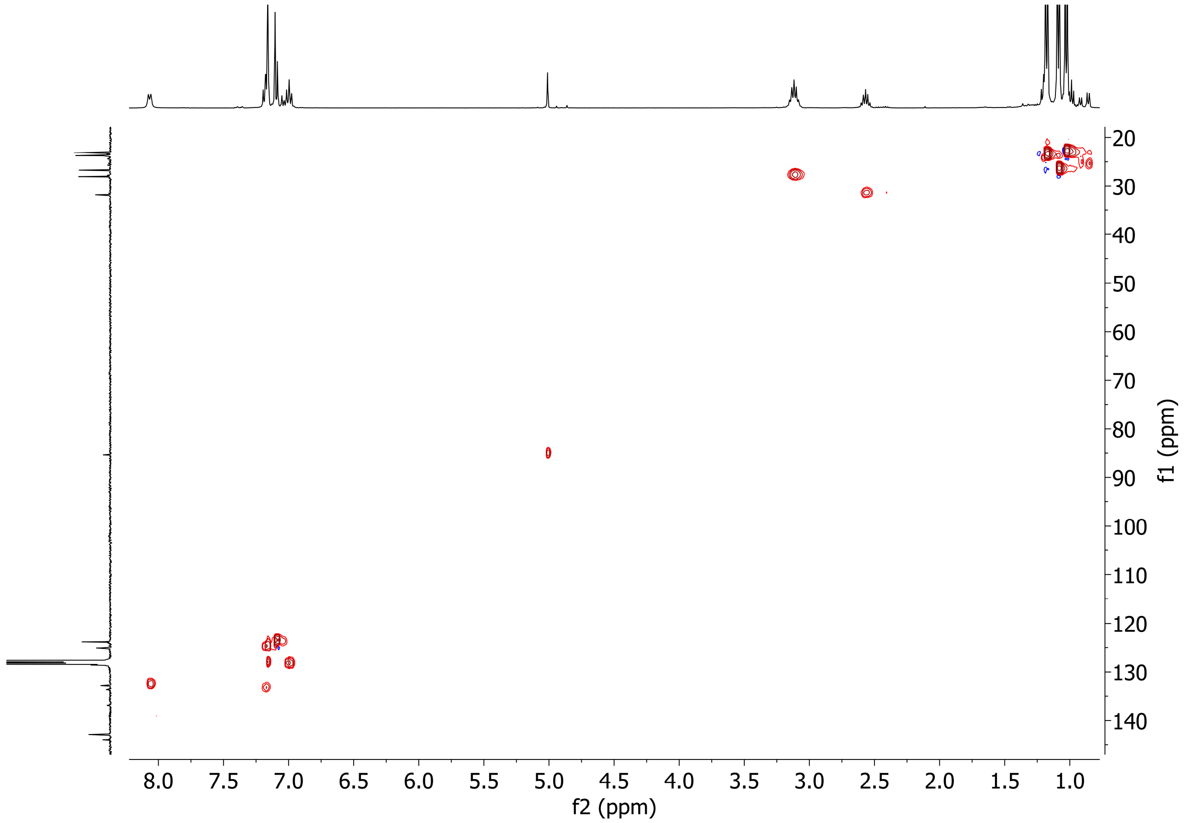

Figure S47. ^{1}H-^{13}C HSQC NMR spectrum of **8b**.

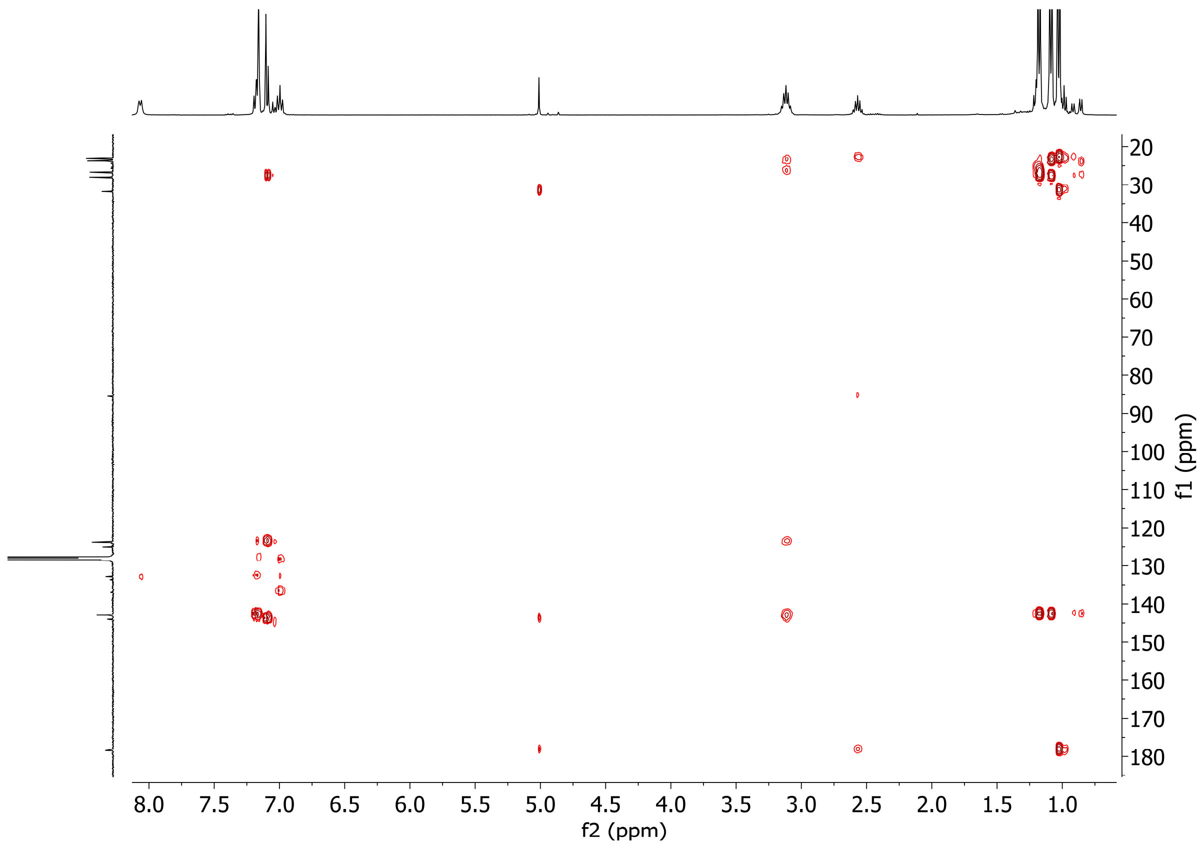

Figure S48. ^{1}H-^{13}C HMBC NMR spectrum of **8b**.

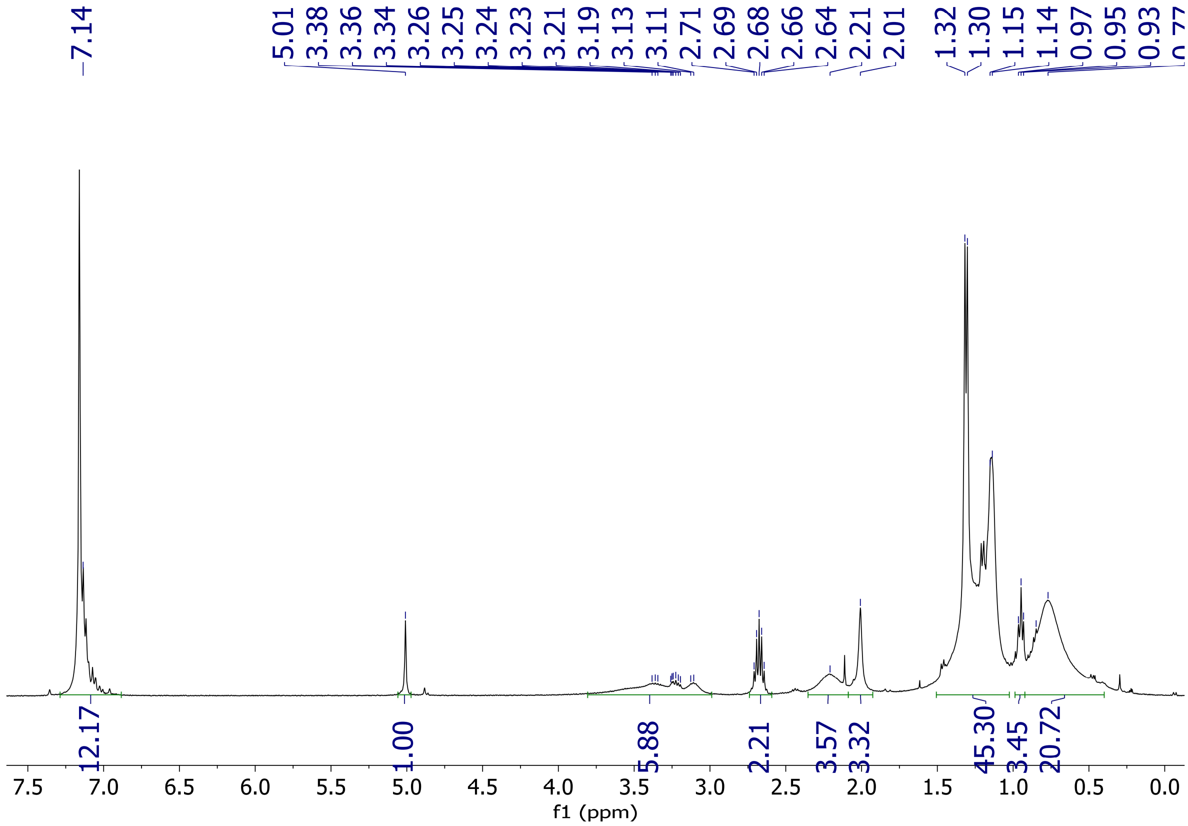

Figure S49. ^{1}H NMR spectrum (400.1 MHz, C_6D_6, 294 K) of *in-situ* generated [{(^{iPrDip}NacNac)Mg(OC*i*Pr$_2$)}$_2$(μ-S)] **8c** from the reaction of [{(^{iPrDip}NacNac)Mg}$_2$(μ-S)] **4** with two equivalents of diisopropylketone. Attempts to further resolve resonances by high temperature NMR spectroscopy were thwarted due to the unstable nature of the species at elevated temperatures. No ^{13}C NMR or 2D NMR spectra were obtained due to the broad nature of the ^{1}H NMR spectrum at this temperature.

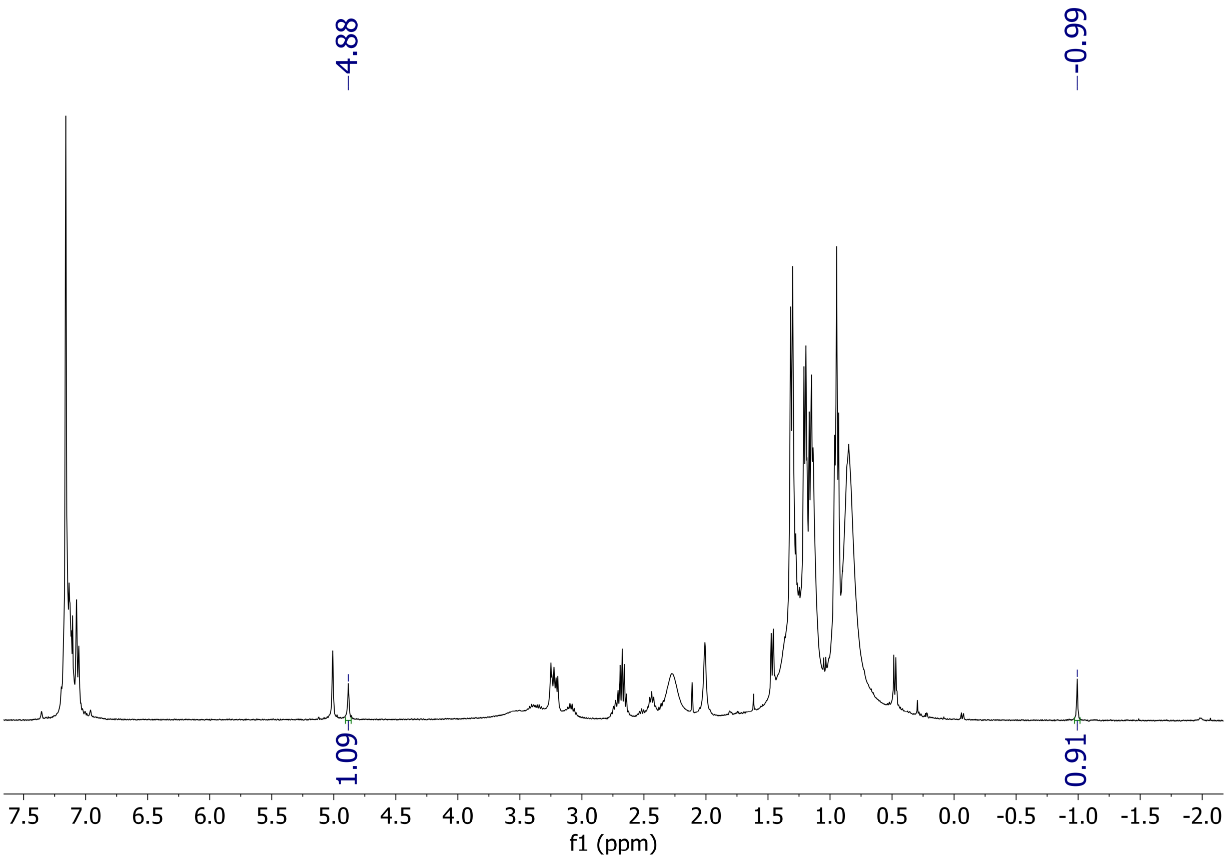

Figure S50. ^{1}H NMR spectrum (400.1 MHz, C_6D_6, 294 K) of [{(^{iPrDip}NacNac)Mg(OC*i*Pr_2)}$_2$(μ-S)] **8c** after heating six hours at 80°C. The two labelled and integrated resonances are likely from the formation of [{(^{iPrDip}NacNac)Mg(μ-SH}$_2$] **9** in the reaction mixture which formed few crystals from the mixture.

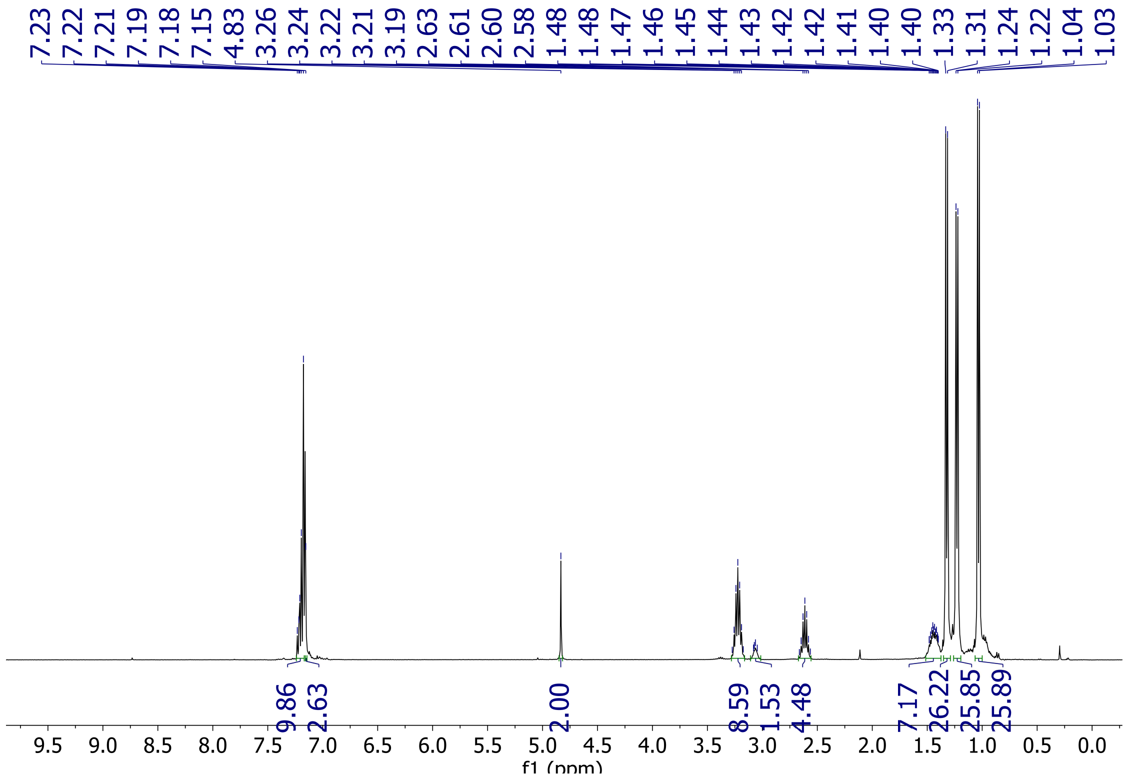

Figure S51. ^{1}H NMR spectrum (400.1 MHz, C_6D_6, 294 K) of *in-situ* generated [{(^{iPrDip}NacNac)Mg(CNCy)}$_2$(μ-S)] **8d** from the reaction of [{(^{iPrDip}NacNac)Mg}$_2$(μ-S)] **4** with two equivalents of cyclohexylisonitrile (CNCy).

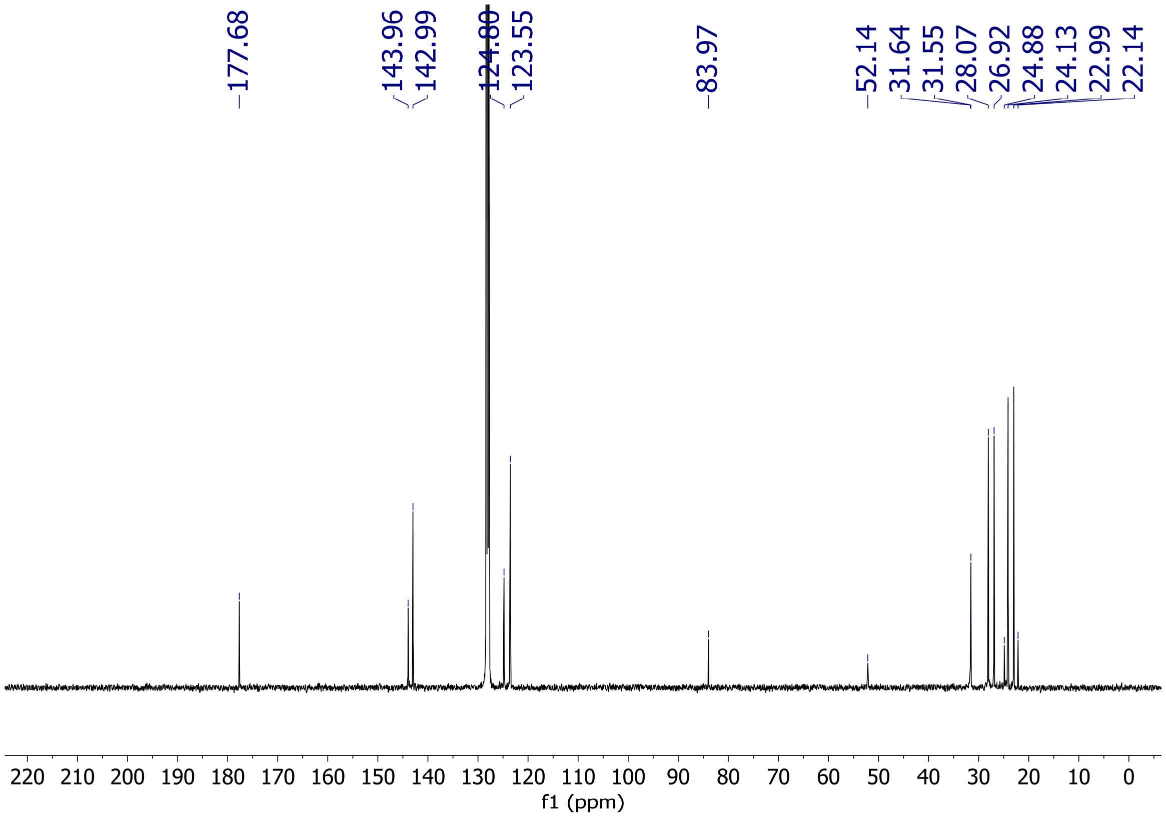

Figure S52. ^{13}C{^{1}H} NMR spectrum (100.5 MHz, C_6D_6, 294 K) of **8d**.

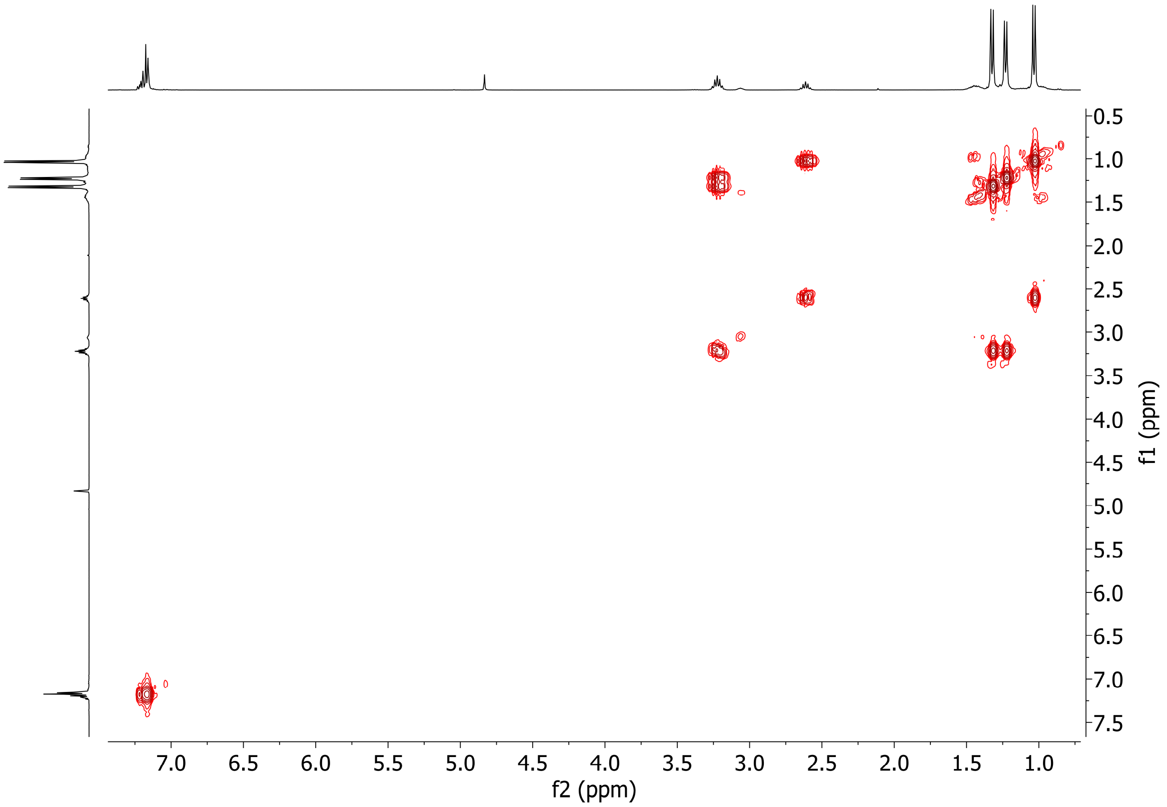

Figure S53. ^{1}H-^{1}H COSY NMR spectrum of **8d**.

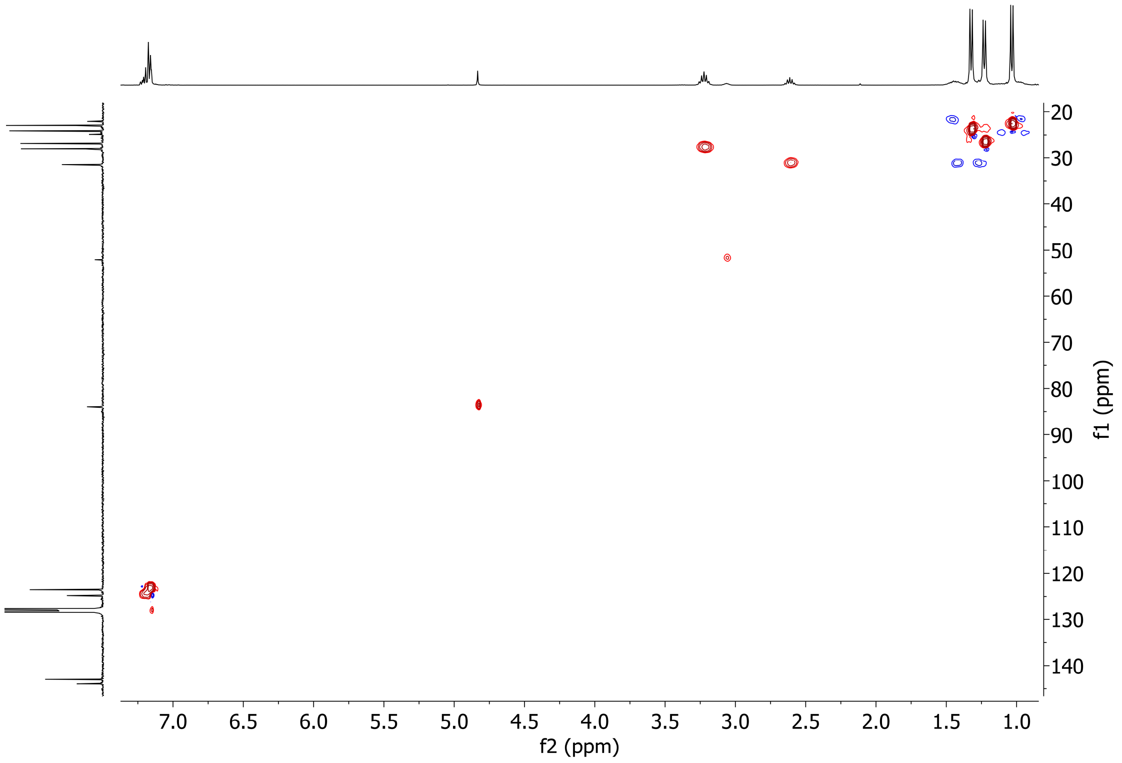

Figure S54. ^{1}H-^{13}C HSQC NMR spectrum of **8d**.

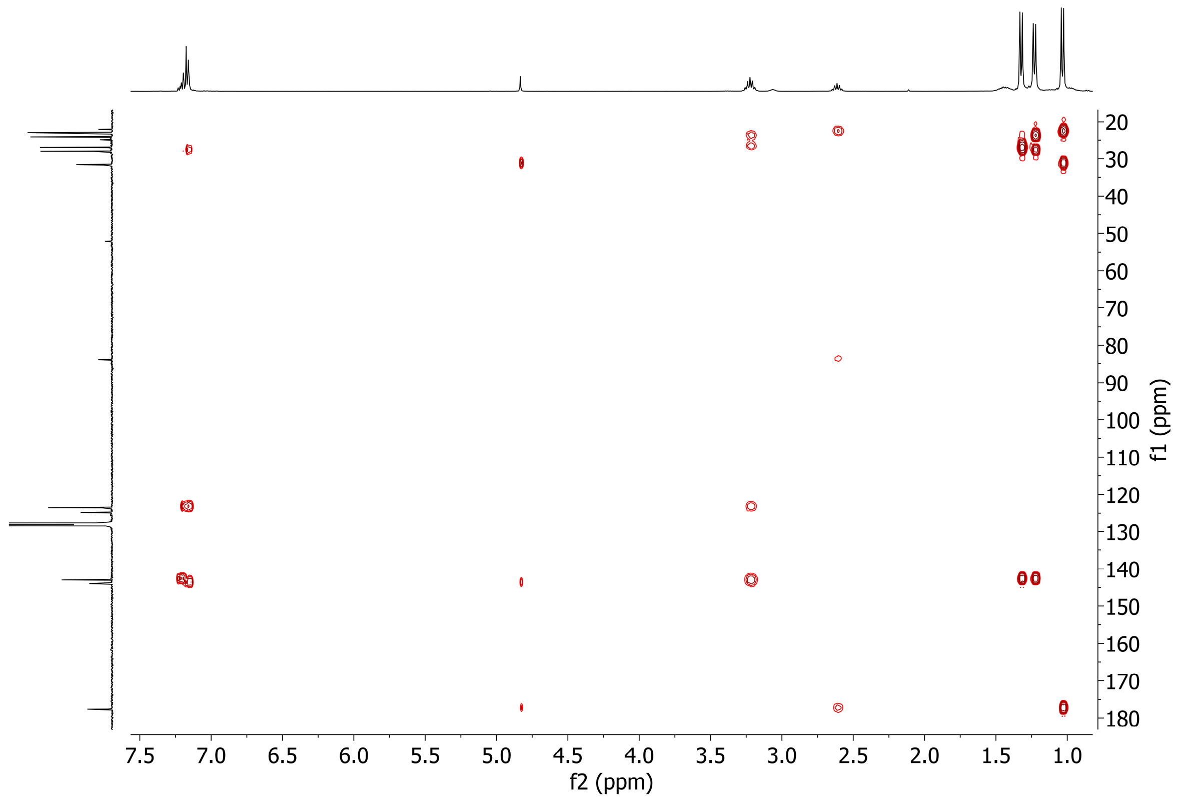

Figure S55. ^{1}H-^{13}C HMBC NMR spectrum of **8d**.

3 X-ray Crystallography

Suitable crystals were mounted in paratone oil and were measured using either a Rigaku FR-X Ultrahigh brilliance Microfocus RA generator/confocal optics with XtaLAB P200 diffractometer (Mo Kα radiation), a Rigaku SCX Mini (Mo Kα radiation), or a Rigaku MM-007HF High Brilliance RA generator/confocal optics with XtaLAB P200 diffractometer (Cu Kα radiation). Data for all compounds analysed were collected using CrystalClear.[6] Data were processed (including correction for Lorentz, polarization, and absorption) using either CrystalClear[6] or CrysAlisPro.[7] Structures were solved by a dual-space method (SHELXT)[8] and refined by full-matrix least-squares against F^2 using SHELXL.[9] All non-hydrogen atoms were refined anisotropically except in selected cases as described below. Hydrogen atoms were placed in calculated positions (riding model) except in one case (**9**) as described below. Crystallographic data is summarised in Table S1 and images of the molecular structures are provided in Figures S53-S59. Details on individual crystal structure determinations and refinements are given below. Further experimental and refinement details are given in the CIF-files. CCDC 2276315-2276321 contains the supplementary crystallographic data for this paper. These data can be obtained free of charge via https://www.ccdc.cam.ac.uk/structures/.

Table S1 Crystallographic data.

Compound reference	**3**	**4**	**4'**	**5**
Chemical formula	$C_{63}H_{74}MgN_2O_2P_2$	$C_{66}H_{98}Mg_2N_4S$	$C_{66}H_{98}Mg_2N_4S$	$C_{66}H_{98}Mg_2N_4Se$
Formula weight	977.49	1028.16	1028.16	1075.06
Temperature/K	173	93	173	173
Radiation type	Mo	Mo	Mo	Cu
Wavelength/Å	0.71075	0.71075	0.71075	1.54184
Crystal system	orthorhombic	tetragonal	tetragonal	tetragonal
Space group	*Pbca*	$I4_1cd$	$I4_1cd$	$I4_1cd$
a/Å	20.8015(10)	17.295(4)	17.2691(7)	17.31100(10)
b/Å	22.7337(11)	17.295(4)	17.2691(7)	17.31100(10)
c/Å	23.8320(13)	43.658(9)	43.5036(18)	43.4612(3)
α/°	90	90	90	90
β/°	90	90	90	90
γ/°	90	90	90	90
Unit cell volume/Å^3	11270.0(10)	13059(7)	12973.7(12)	13024.05(18)
No. of formula units per unit cell, Z	8	8	8	8
Density (calc)/ Mg/m^3	1.152	1.046	1.053	1.097
Absorption coefficient, μ/mm^{-1}	0.132	0.108	0.109	1.254
F(000)	4192	4496	4496	4640
Theta range/°	1.709 to 25.342	1.866 to 25.407	1.872 to 25.357	4.069 to 75.393
Reflections collected	70192	68455	36138	73533
Independent reflections	10297	5928	5721	6647
R_{int}	0.0377	0.0366	0.0273	0.0350
Completeness (to theta)/%	99.9 (max)	99.5 (max)	99.9 (max)	99.7 (max)
Data / restraints / parameter	10297 / 36 / 701	5928 / 1 / 360	5721 / 10 / 356	5721 / 10 / 356
Goodness of fit on F^2	1.017	1.054	1.046	1.052
Final R_1 values ($I > 2\sigma(I)$)	0.0464	0.0270	0.0268	0.0316
Final $wR(F^2)$ values ($I > 2\sigma(I)$)	0.1276	0.0705	0.0683	0.0863
Final R_1 values (all data)	0.0692	0.0317	0.0317	0.0334
Final $wR(F^2)$ values (all data)	0.1410	0.0724	0.0699	0.0879
Largest diff. peak and hole/e·Å^{-3}	0.424 and -0.242	0.119 and -0.112	0.107 and -0.135	0.138 and -0.239
CCDC number	2276318	2276321	2276316	2276320

Table S1 continued Crystallographic data.

Compound reference	**7**·C_6H_6	**8a**·4C_6H_6	**9**·2 C_6H_6
Chemical formula	$C_{82}H_{119}Mg_2N_7S$	$C_{110}H_{150}Mg_2N_4O_2S$	$C_{78}H_{112}Mg_2N_4S_2$
Formula weight	1283.51	1641.01	1218.45
Temperature/K	173	125	173
Radiation type	Cu	Cu	Mo
Wavelength/Å	1.54184	1.54184	0.71075
Crystal system	monoclinic	monoclinic	monoclinic
Space group	$P2_1/c$	$I2/a$	$P2_1/n$
a/Å	12.69600(10)	17.2490(2)	15.2241(10)
b/Å	22.46710(10)	21.6070(2)	13.2463(9)
c/Å	28.3671(2)	26.5064(3)	19.5886(13)
α/°	90	90	90
β/°	102.5820(10)	97.3030(10)	106.876(6)
γ/°	90	90	90
Unit cell volume/Å^3	7897.18(10)	9798.77(18)	3780.2(5)
No. of formula units per unit cell, Z	4	4	2
Density (calc)/ Mg/m^3	1.080	1.112	1.070
Absorption coefficient, μ/mm^{-1}	0.854	0.795	0.109
F(000)	2800	3576	1328
Theta range/°	2.533 to 75.419	2.647 to 75.432	2.078 to 27.486
Reflections collected	93114	54976	38195
Independent reflections	16007	9899	8633
R_{int}	0.0327	0.0218	0.0714
Completeness (to theta)/%	97.8 (max)	97.6 (max)	99.6 (max)
Data / restraints / parameter	16007 / 66 / 907	9899 / 47 / 572	8633 / 9 / 422
Goodness of fit on F^2	1.078	1.037	1.017
Final R_1 values ($I > 2\sigma(I)$)	0.0451	0.0671	0.0618
Final $wR(F^2)$ values ($I > 2\sigma(I)$)	0.1217	0.1865	0.1637
Final R_1 values (all data)	0.0503	0.0675	0.0963
Final $wR(F^2)$ values (all data)	0.1257	0.1869	0.1913
Largest diff. peak and hole/e·Å^{-3}	0.374 and -0.462	0.597 and -0.421	0.963 and -0.429
CCDC number	2276319	2276317	2276315

[(^{iPrDip}NacNac)Mg(OPPh$_2$)(OPPh$_3$)] 3

The compound crystallised with a full molecule in the asymmetric unit (Pbca). The diphenylphosphinite unit is disordered and was modelled with two positions for the Ph$_2$P unit, that were freely refined to 82 and 18% using geometry restraints (DFIX, DANG, FLAT) for the minor part. The minor parts of the Ph groups were refined isotropically.

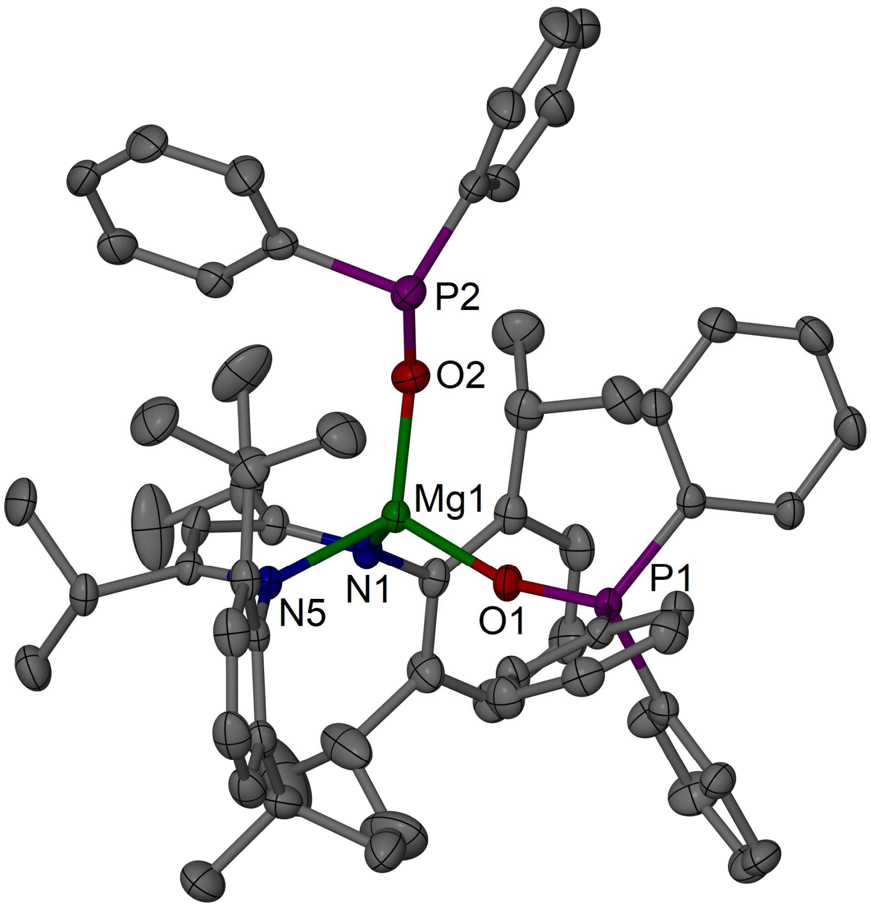

Figure S56. Molecular structure of [(^{iPrDip}NacNac)Mg(OPPh$_3$)(OPPh$_2$)] **3** (30% thermal ellipsoids). Only the major Ph$_2$P parts is shown. Hydrogen atoms are omitted. Selected bond lengths (Å) and angles (°): P1-O1 1.4972(13), P2-O2 1.5459(17), Mg1-O2 1.9000(16), Mg1-O1 1.9243(14), Mg1-N1 2.0511(17), Mg1-N5 2.0767(16); O2-Mg1-O1 109.13(7), N1-Mg1-N5 95.12(6), P1-O1-Mg1 162.83(9), P2-O2-Mg1 143.26(11).

[{(^{iPrDip}NacNac)Mg}$_2$(μ-S)] 4

Data for two similar datasets (**4**, **4'**) of isomorphous crystals are provided. The molecules crystallised in the tetragonal crystals system in space group $I4_1cd$ with half a molecule in the asymmetric unit. The sulfur positions were found to be disordered on or very close to special positions and were modelled with three positions in the asymmetric unit that are close to each other. The occupancies of the sulfur positions were assigned manually, and changed and refined in small steps, considering individual *U*iso values and overall *R* values and were adjusted to add to a total of one S per molecule. For the second dataset (**4'**), the RIGU command was used to refine the Mg and S atoms.

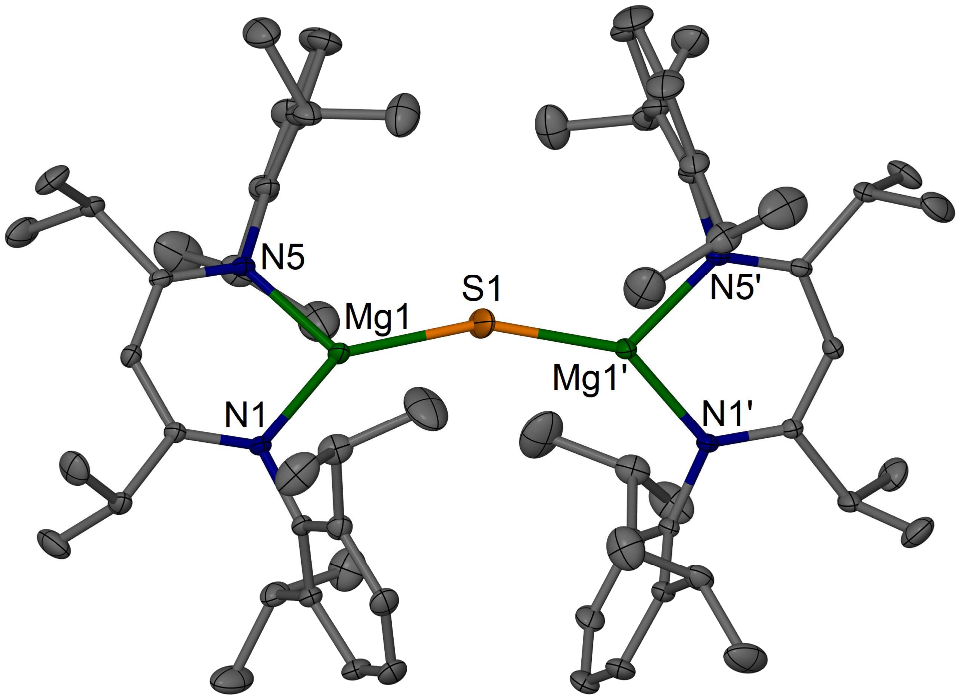

Figure S57. Molecular structure of [{(^{iPrDip}NacNac)Mg}$_2$(μ-S)] **4** (30% thermal ellipsoids). Hydrogen atoms are omitted, only one S position is shown. Selected bond lengths (Å) and angles (°): Shown structure (**4**): Mg1-S1 2.243(3), Mg1'-S2 2.327(18), Mg1-S2 2.22(2), Mg1-S3 2.21(4), Mg1'-S3 2.23(4), Mg1-N1 2.0126(16), Mg1-N5 2.0209(16); Mg1-S1-Mg1' 158.2(9), Mg1-S2-Mg1' 151.1(9), Mg1-S3-Mg1' 167(3), N1-Mg1-N5 95.87(6). Data for isomorphous structure (**4'**): Mg1-S1 2.255(9), Mg1'-S1 2.259(15), Mg1-N5 2.0072(15), Mg1-N1 2.0146(16), Mg1-S2 2.230(5), Mg1-S3 2.228(3); Mg1-S1-Mg1' 153.7(11), Mg1-S2-Mg1' 160.5(15), Mg1-S3-Mg1' 161.2(9), N5-Mg1-N1 95.78(6).

[{(^{iPrDip}NacNac)Mg}$_2$(μ-Se)] 5

The molecule crystallised isomorphous to that of the sulfur analogue **4**. The selenium positions were found to be disordered on or very close to special positions and were modelled with three positions in the asymmetric unit that are close to each other with Se1 in the main position. The occupancies of the Se positions were assigned manually, and changed and refined in small steps, considering individual *U*iso values and overall *R* values and were adjusted to add to a total of one Se per molecule.

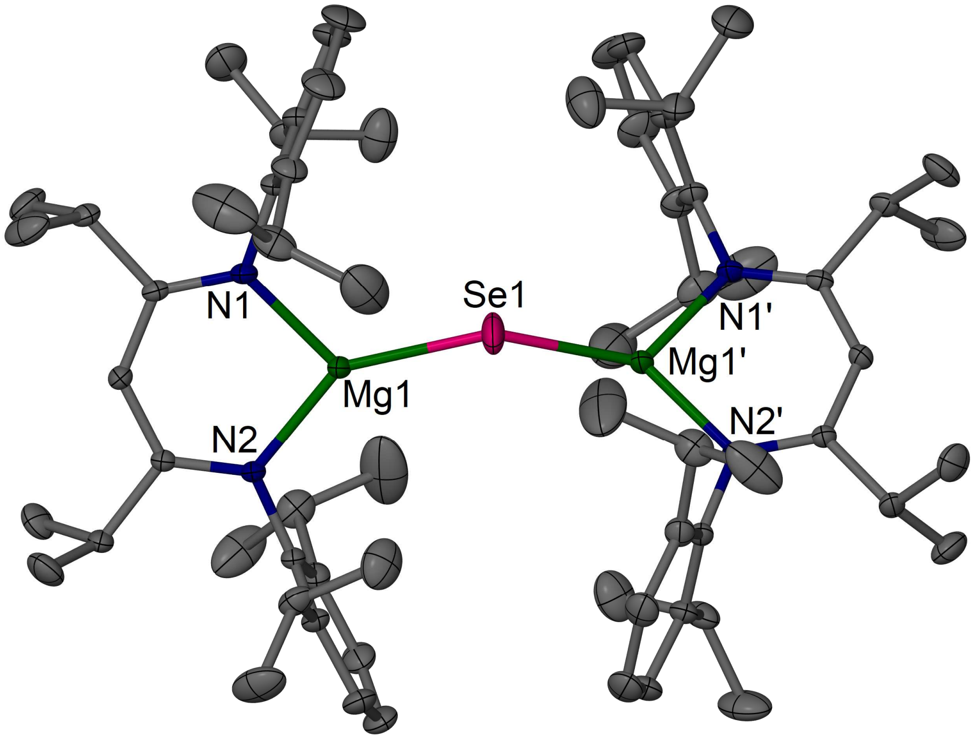

Figure S58. Molecular structure of [{(^{iPrDip}NacNac)Mg}$_2$(μ-Se)] **5** (30% thermal ellipsoids). Hydrogen atoms are omitted, only one Se position is shown. Selected bond lengths (Å) and angles (°): Mg1-Se1 2.3497(18), Mg1'-Se1 2.4739(18), Mg1-Se2 2.3185(10), Mg1-Se3 2.294(6), Mg1'-Se3 2.401(6), Mg1-N2 2.0071(16), Mg1-N1 2.0130(16); Mg1-Se1-Mg1' 137.89(6), Mg1-Se2-Mg1' 152.25(17), Mg1-Se3-Mg1' 147.0(3), N2-Mg1-N1 96.35(6).

[{(^{iPrDip}NacNac)Mg}$_2$(μ-SN$_3$Ad)] 7

The compound crystallised with a full molecule and one benzene molecule in asymmetric unit. The benzene molecule is significantly disordered and was modelled with two positions for each atom using geometry restraints (DFIX, DANG, FLAT) and refined anisotropically. The RIGU command was used to refine some aryl carbon atoms (C64-C69).

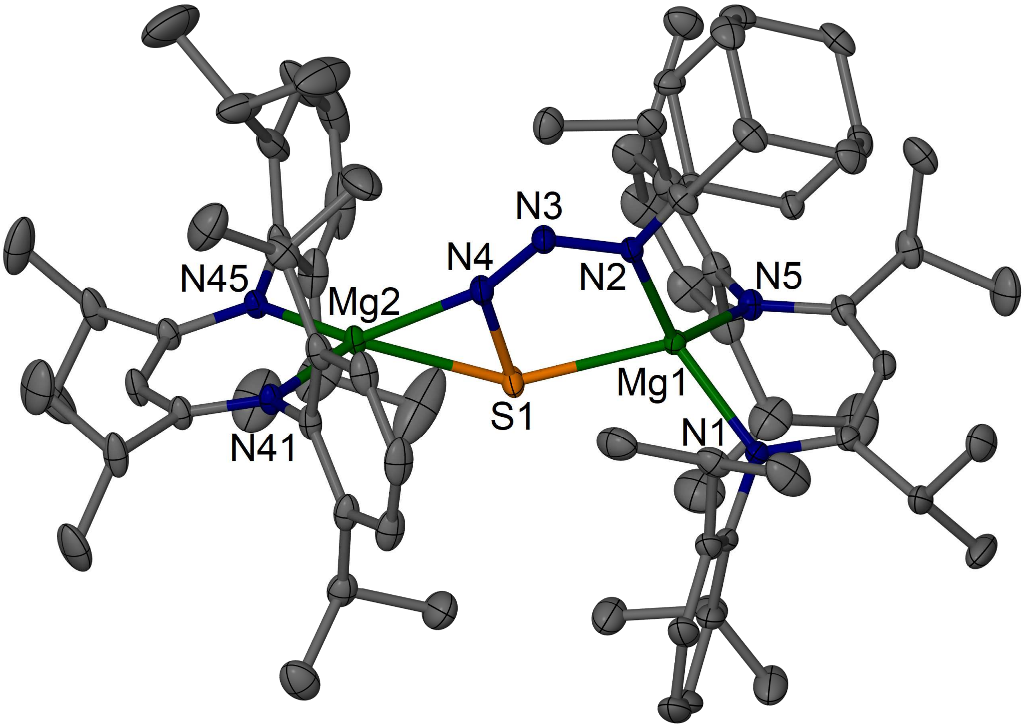

Figure S59. Molecular structure of [{(^{iPrDip}NacNac)Mg}$_2$(μ-SN$_3$Ad)]·C_6H_6, **7**·C_6H_6 (30% thermal ellipsoids). Hydrogen atoms and solvent molecule are omitted. Selected bond lengths (Å) and angles (°): Mg1-S1 2.3926(5), Mg2-S1 2.4343(5), Mg1-N1 2.0443(11), Mg1-N5 2.0523(11), Mg1-N2 2.0944(11), Mg2-N45 2.0111(11), Mg2-N41 2.0098(11), Mg2-N4 2.0123(11), S1-N4 1.8257(12), N2-N3 1.3061(15), N3-N4 1.2726(16), N2-C76 1.4936(15); Mg1-S1-Mg2 148.43(2), N4-S1-Mg1 94.98(4), N4-S1-Mg2 54.12(4), N1-Mg1-N5 95.48(5), N2-Mg1-S1 80.86(3), N41-Mg2-N45 97.27(4), N4-Mg2-S1 47.32(3), N4-N3-N2 120.52(11).

[(^{iPrDip}NacNac)Mg(OAd)}$_2$(μ-S)] 8a

The compound crystallised with half a molecule and some disordered benzene in the asymmetric unit. In the asymmetric unit, half a benzene molecule (C50-52) was refined anisotropically with geometry restraints, a full benzene was modelled with two positions for each atom set to 50% occupancy and refined using geometry restrains, one part isotropic, one part anisotropic. One half benzene molecule was modelled with six CH positions with 50% occupancy using geometry restraints.

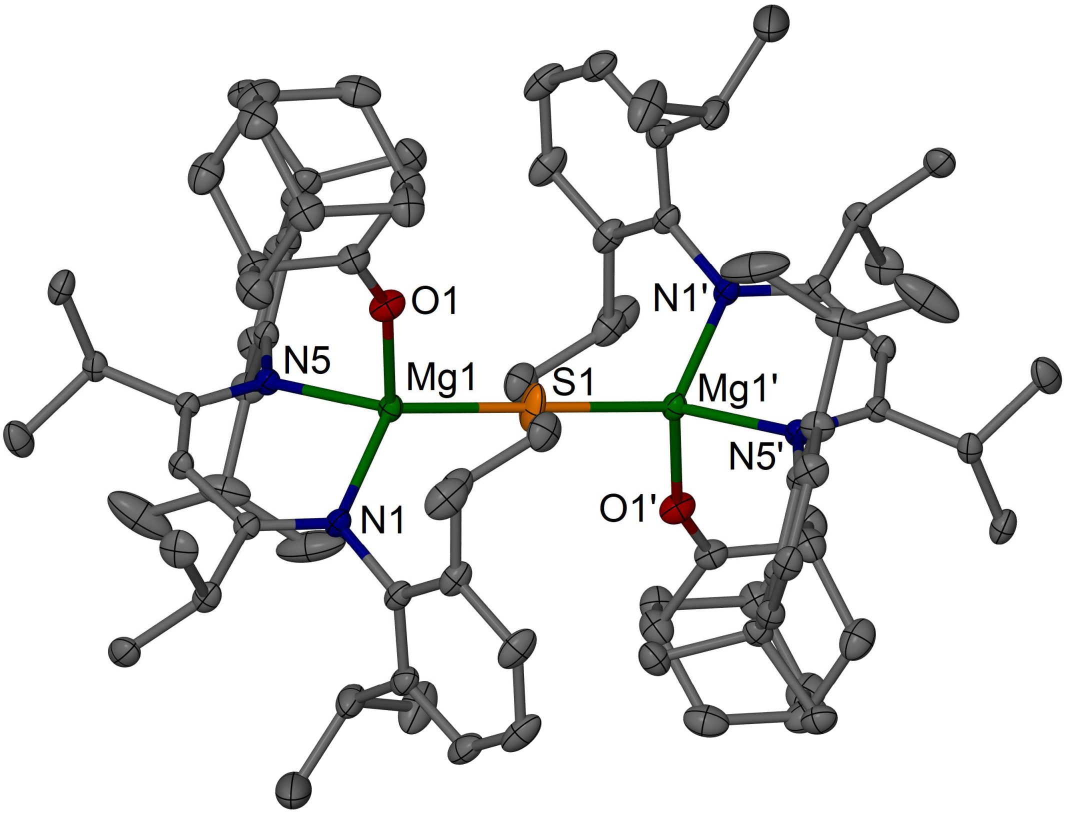

Figure S60. Molecular structure of [(^{iPrDip}NacNac)Mg(OAd)}$_2$(μ-S)] ·4C_6H_6, **8a** ·4C_6H_6 (30% thermal ellipsoids). Hydrogen atoms and solvent molecule are omitted. Selected bond lengths (Å) and angles (°): Mg1-S1 2.2610(5), Mg1'-S1 2.2610(5), Mg1-N1 2.0723(14), Mg1-N5 2.0733(14), Mg1-O1 2.0875(14), O1-C36 1.225(2); Mg1-S1-Mg1' 180.0, N1-Mg1-N5 94.14(5), N1-Mg1-O1 101.64(6), N5-Mg1-O1 101.49(6), N1-Mg1-S1 122.81(4), N5-Mg1-S1 124.03(4), O1-Mg1-S1 108.91(5).

[{(^{iPrDip}NacNac)Mg(μ-SH)}$_2$] 9

The compound crystallised with half a molecule in the asymmetric unit and one benzene molecule. The latter was modelled and refined as a perfect hexagon. One isopropyl group (C33-C35) is disordered and was modelled with two positions for the atoms using geometry restraints (DFIX, DANG) and the occupancies were freely refined anisotropically to 52 and 48% parts. The SH hydrogens were found and a geometry restraint for the S-H bond (DFIX) was used in their refinement.

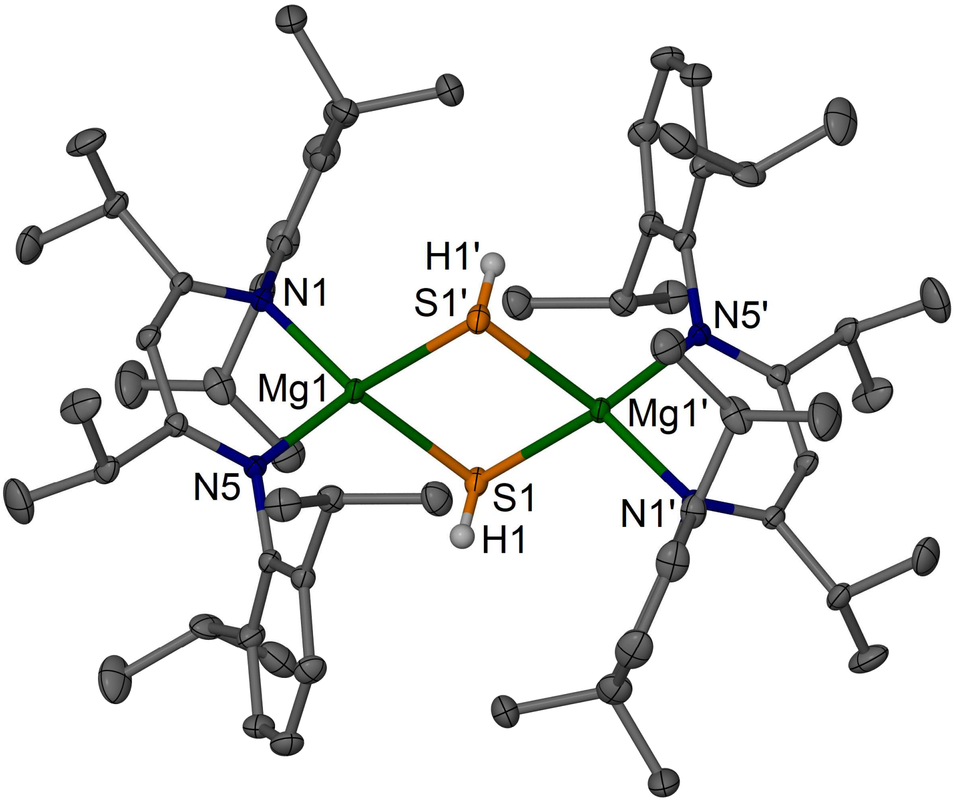

Figure S61. Molecular structure of [{(^{iPrDip}NacNac)Mg(μ-SH)}$_2$] $\cdot 2C_6H_6$, **9** $\cdot 2C_6H_6$ (30% thermal ellipsoids). Hydrogen atoms and solvent molecules are omitted. Selected bond lengths (Å) and angles (°): Mg1-S1 2.5109(9), Mg1'-S1 2.5183(9), Mg1-N5 2.0468(19), Mg1-N1 2.0593(18); Mg1-S1-Mg1' 89.52(3), N5-Mg1-N1 94.98(7), S1-Mg1-S1' 90.48(3).

4 DFT Computational Studies

The complexes [{(^{iPrDip}NacNac)Mg}$_2$(μ-E)], E = O (**2**), S (**4**), Se (**5**), and the cut-back models [{(^{MeMe}NacNac)Mg}$_2$(μ-E)], were optimised in the gas phase from the starting geometries obtained by X-ray diffraction using the M06-L[10] density functional coupled with the def2-TZVP basis set augmented with a D3 dispersion term,[11] followed by single point calculations at the M06/def2-TZVP level.[12] In addition, the small models [{(^{MeMe}NacNac)Mg}$_2$(μ-E)] were optimised at the same level without D3 dispersion addition. Natural Population Analysis (NPA) charges and Wiberg bond orders were calculated using Natural Bond Orbital (NBO) analysis,[13] at the M06-D3/def2-TZVP level. Quantum Theory of Atoms in Molecules (QTAIM) analysis was conducted at the same level. The calculations were performed using Gaussian 16.[14] QTAIM analysis was carried out using AIMAll.[15]

The Wiberg bond indices (WBIs) for the two Mg–E bonds in [{(^{iPrDip}NacNac)Mg}$_2$(μ-E)] are 0.047 and 0.047 (E = O, **2**), 0.211 and 0.210 (E = S, **4**), and 0.252 and 0.252 (E = Se, **5**).

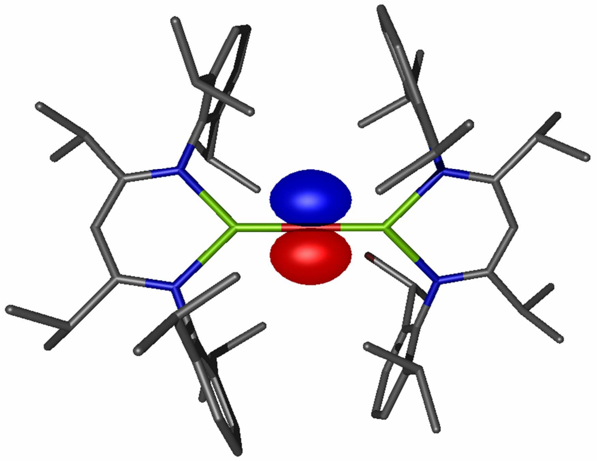

HOMO-3 (-6.11 eV)

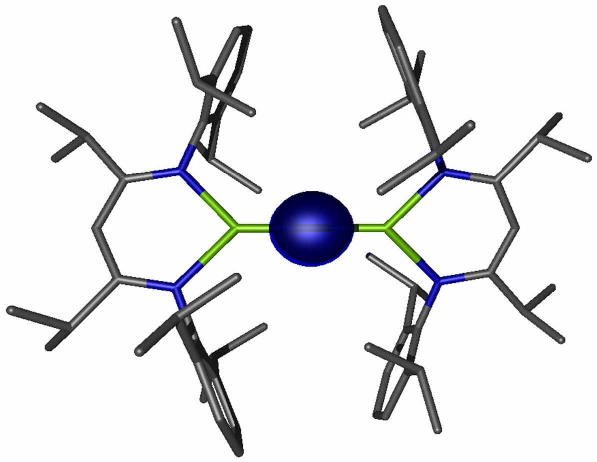

HOMO-4 (-6.20 eV)

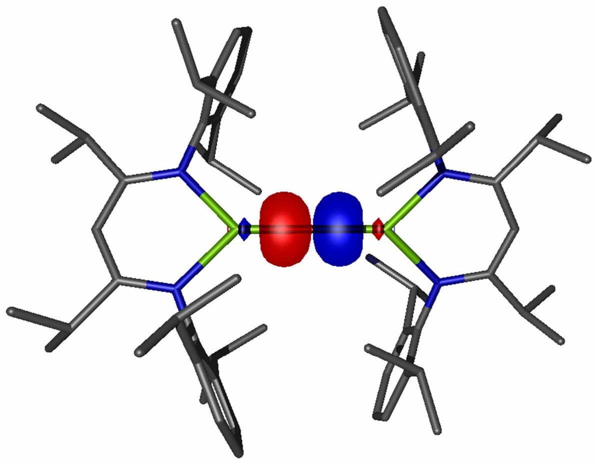

HOMO-12 (-7.59 eV)

Figure S62. Selected orbitals (chalcogen *p*-orbitals) of [{(^{iPrDip}NacNac)Mg}$_2$(μ-O)] **2** (isovalue 0.06).

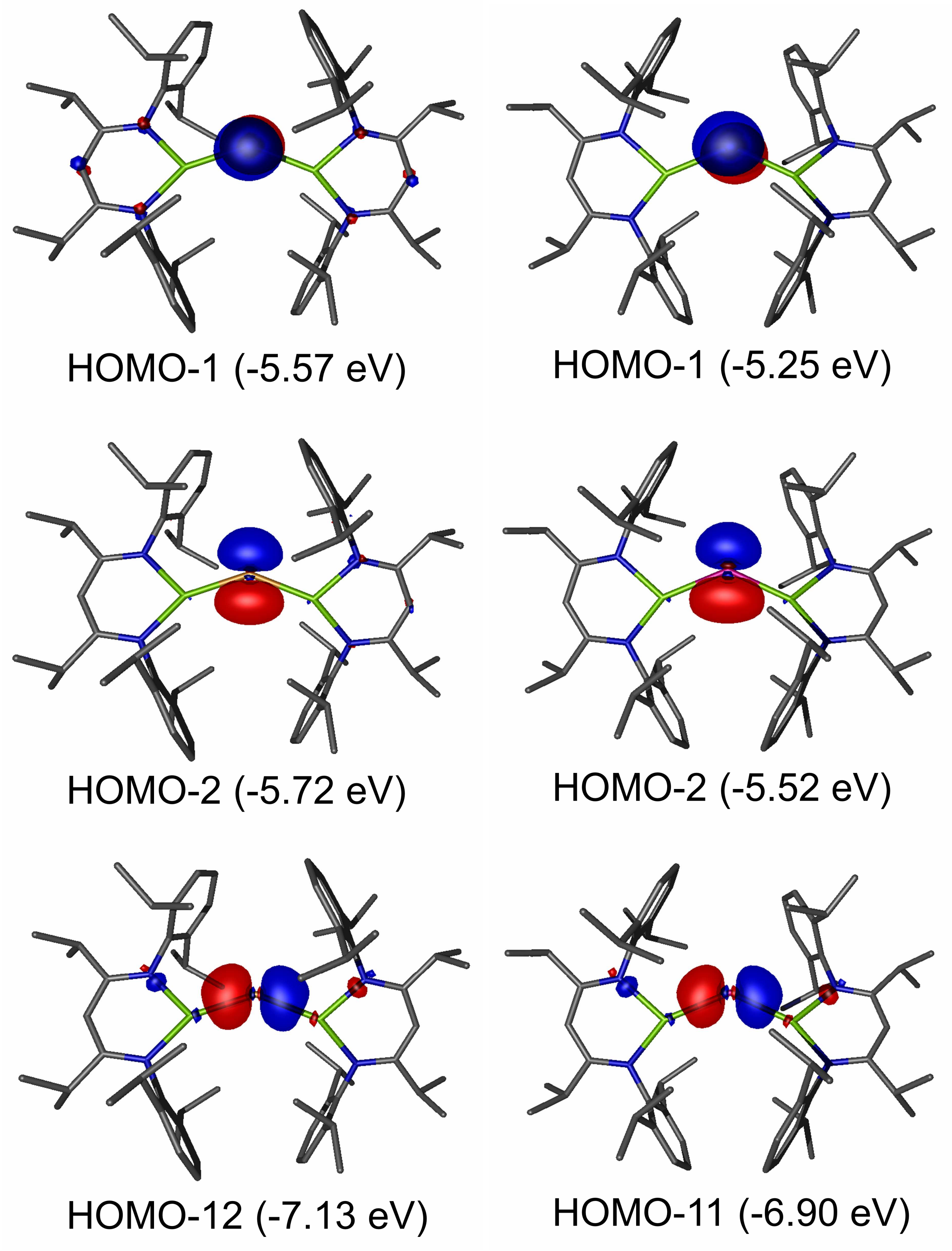

Figure S63. Selected orbitals (chalcogen *p*-orbitals) of [{(^{iPrDip}NacNac)Mg}$_2$(μ-S)], **4**, left, and [{(^{iPrDip}NacNac)Mg}$_2$(μ-Se)], **5**, right (isovalue 0.06).

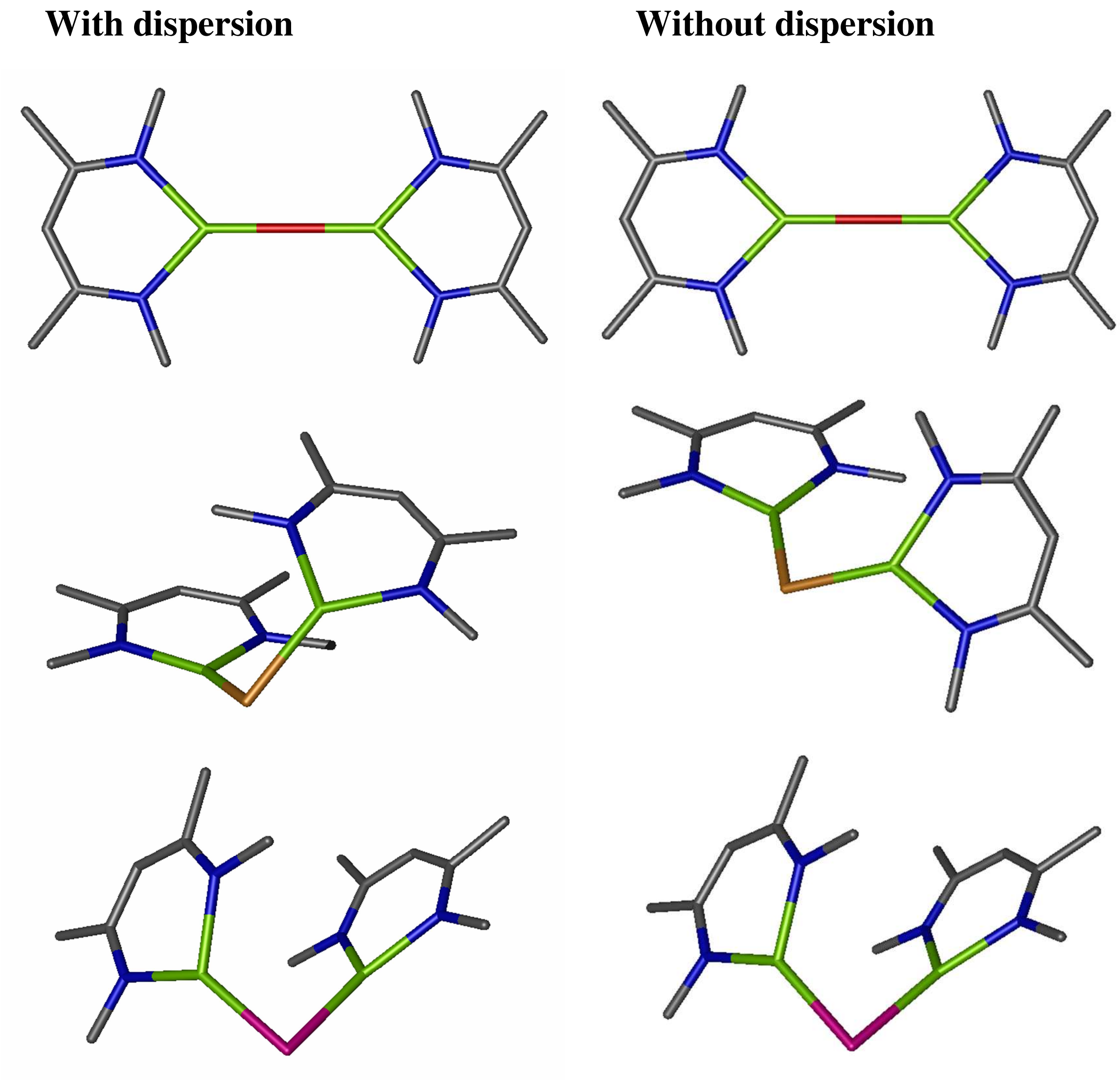

Figure S64. Optimised geometries of [{(^{MeMe}NacNac)Mg}$_2$(μ-E)]; top: E = O, centre: E = S, bottom: E = Se; hydrogen atoms omitted. The geometries on the left have been obtained with dispersion addition (+D3), the ones on the right have been obtained without dispersion addition. Please note, however, that the M06-L functional is able to capture some dispersion effects (which appear to have an effect on the Se derivative). The difference mainly affects the geometry of the molecule for E = S; the Mg–E–Mg angles are 179.9/180.0° (left/right, E = O), 87.6/106.4° (E = S), 82.5/82.6° (E = Se).

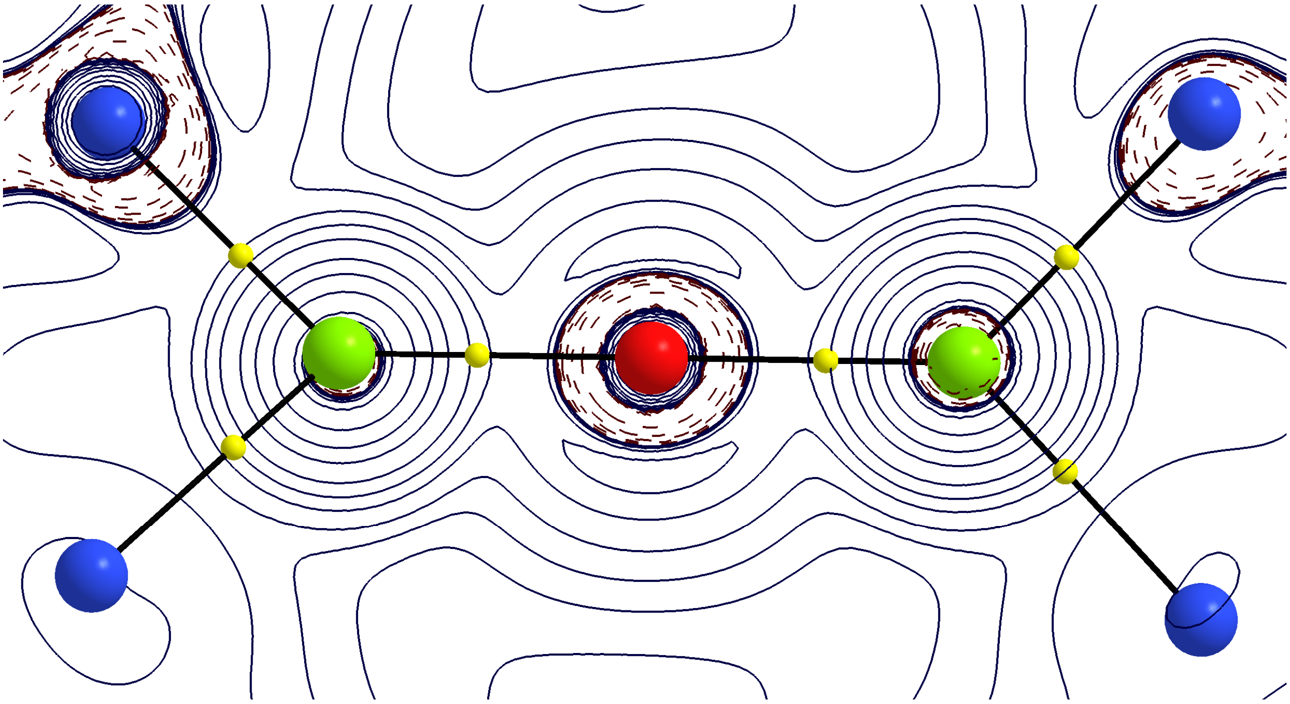

Figure S65. QTAIM contour plots of the Laplacian of electron density (solid lines positive, dashed lines negative) for [{(^{iPrDip}NacNac)Mg}$_2$(μ-O)] **2** through the Mg_2O plane showing only the core atoms (Mg green, O red, N blue) and selected bond paths (black) and bond critical points (yellow). Selected values for the electron density, ρ, Laplacian, $\nabla^2\rho$, and bond ellipticity, ε, are given for key bond critical points in Table S2.

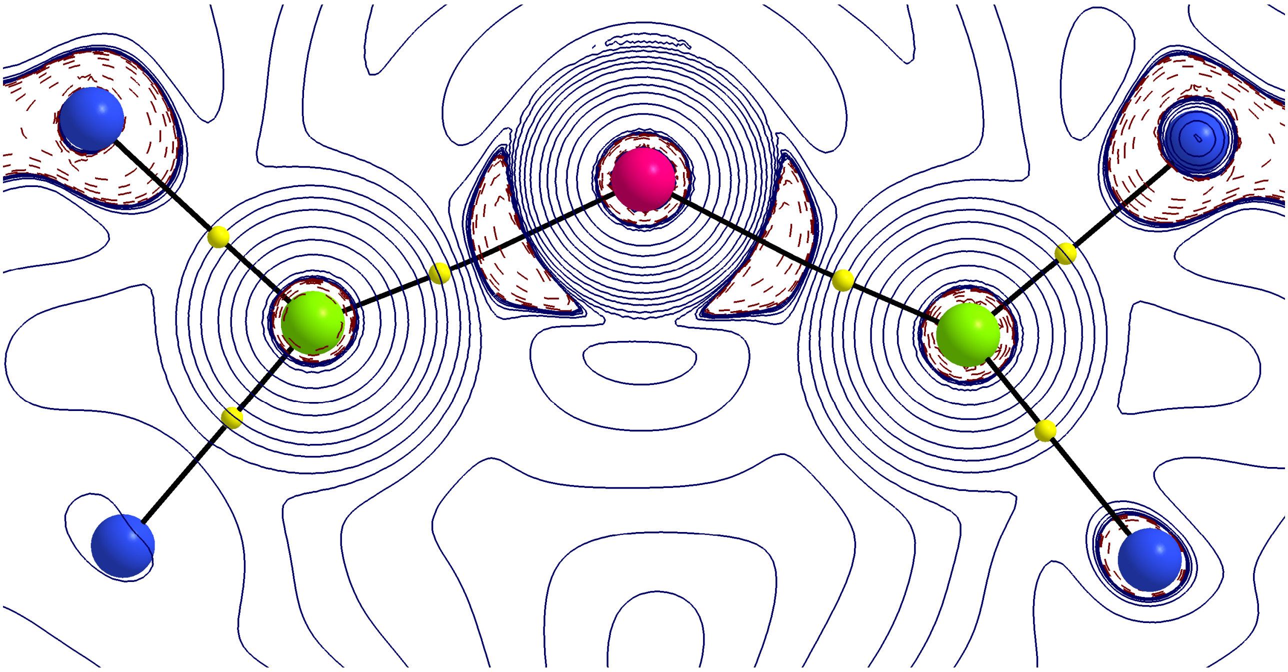

Figure S66. QTAIM contour plots of the Laplacian of electron density (solid lines positive, dashed lines negative) for $[\{(^{iPrDip}NacNac)Mg\}_2(\mu\text{-}Se)]$, **5**, through the Mg_2Se plane showing only the core atoms (Se magenta, Mg green, N blue) and selected bond paths (black) and bond critical points (yellow). Selected values for the electron density, ρ, Laplacian, $\nabla^2\rho$, and bond ellipticity, ε, are given for key bond critical points in Table S2.

Table S2: Selected values for bond critical points on Mg–E bonds (QTAIM)

**bcp on Mg-E bond**	**electron density, ρ [e/bohr^3]**	**Laplacian, $\nabla^2\rho$ [e/bohr^5]**	**bond ellipticity, ε**
[{(^{iPrDip}NacNac)Mg}$_2$(μ-O)] **2**			
bcp (Mg1, O2)	0.078	0.655	0.0008
bcp (Mg46, O2)	0.078	0.655	0.0008
[{(^{iPrDip}NacNac)Mg}$_2$(μ-S)] **4**			
bcp (Mg87, S86)	0.051	0.244	0.034
bcp (Mg1, S86)	0.051	0.244	0.032
[{(^{iPrDip}NacNac)Mg}$_2$(μ-Se)] **5**			
bcp (Mg1, Se4)	0.046	0.187	0.055
bcp (Mg87, Se4)	0.046	0.187	0.055

Atomic coordinates (XYZ) (in Å)

LMgOMgL species

Mg-O-Mg full, [{(^{iPrDip}NacNac)Mg}$_2$(μ-O)] **2**
Mg -1.80530900 0.00009900 0.00007200
O 0.00002100 -0.00026500 0.00035900
N -3.18436500 -1.39521600 0.52607900
C -5.07405200 0.00048600 -0.00036300
H -6.15433000 0.00061300 -0.00051100
C -2.67806200 -2.64604100 0.97690500
C -2.34505900 -3.62795900 0.02873400
C -4.49663300 -1.17766700 0.48599500
C -5.47876800 -2.24661600 0.95451500
H -4.89972400 -3.04497300 1.42066500
C -2.48155500 -2.87199300 2.34978700
C -1.63951800 -5.07670000 1.83141400
H -1.23956600 -6.02641200 2.16455500
C -2.77594100 -1.77711400 3.35095000
H -3.54115300 -1.12838000 2.91687200
C -1.81613300 -4.83330000 0.48038700
H -1.55106500 -5.59893600 -0.23830200
C -1.97162300 -4.10021200 2.75544800
H -1.82309900 -4.29188300 3.81124300
C -2.53815000 -3.35875300 -1.44790700
H -3.37719800 -2.66315200 -1.54464400
C -6.44968600 -1.72017600 2.00518200
H -7.14535400 -0.99010000 1.59078200
H -5.92813300 -1.24131400 2.83386700
H -7.04331500 -2.53858600 2.41358100
C -6.22526400 -2.85388800 -0.22841500
H -6.89311300 -3.64824900 0.10581300
H -5.54055700 -3.28131700 -0.96023600
H -6.82984400 -2.10407700 -0.74080400
C -1.30435300 -2.67803200 -2.03486400
H -0.99115100 -1.78477900 -1.48526600
H -1.47054300 -2.39545700 -3.07559400
H -0.44794000 -3.35244600 -2.00279100
C -1.52609800 -0.92410500 3.55894300
H -0.73294900 -1.51346100 4.02343200
H -1.72788700 -0.06788400 4.20416500
H -1.12045800 -0.54746100 2.61429200
C -3.31454400 -2.29038000 4.67684700
H -4.18319900 -2.93399600 4.53477600
H -3.61398300 -1.45889500 5.31484500
H -2.56675500 -2.86106100 5.22872300
C -2.87648900 -4.60472300 -2.25291900
H -2.02883900 -5.28925200 -2.31115800
H -3.13573900 -4.33566300 -3.27672600
H -3.71625900 -5.15304900 -1.82569600
Mg 1.80534200 -0.00020000 0.00051900
N 3.18403100 -1.39576700 -0.52588800
C 5.07409200 -0.00024400 -0.00025500
H 6.15437100 -0.00027900 -0.00047100
C 2.67741700 -2.64646500 -0.97670000
C 2.34420200 -3.62830600 -0.02854300
C 4.49633800 -1.17835100 -0.48633700
C 5.47816700 -2.24746700 -0.95513300
H 4.89887400 -3.04568900 -1.42120100
C 2.48087300 -2.87237900 -2.34960000
C 1.63841600 -5.07692700 -1.83122200
H 1.23830800 -6.02657000 -2.16437100
C 2.77544300 -1.77753200 -3.35074400
H 3.54092800 -1.12906000 -2.91675900
C 1.81504200 -4.83355200 -0.48019800
H 1.54978900 -5.59911900 0.23849700
C 1.97074000 -4.10050600 -2.75526200
H 1.82222000 -4.29216800 -3.81105800
C 2.53732900 -3.35914000 1.44810500
H 3.37650200 -2.66368400 1.54483900
C 6.44903900 -1.72125000 -2.00594900
H 7.14502700 -0.99142000 -1.59165600
H 5.92746400 -1.24218200 -2.83450400
H 7.04233600 -2.53981600 -2.41452000
C 6.22470200 -2.85493200 0.22768200
H 6.89230700 -3.64945400 -0.10664700
H 5.53999200 -3.28219000 0.95959800

H	6.82953200	-2.10525600	0.73997900
C	1.30364700	-2.67819200	2.03504900
H	0.99066000	-1.78484600	1.48547100
H	1.46982900	-2.39570800	3.07580400
H	0.44709600	-3.35243100	2.00289300
C	1.52580600	-0.92415400	-3.55845300
H	0.73242100	-1.51323500	-4.02288800
H	1.72771300	-0.06789600	-4.20359300
H	1.12040300	-0.54752000	-2.61370000
C	3.31364600	-2.29085200	-4.67678100
H	4.18210400	-2.93478500	-4.53492400
H	3.61326900	-1.45940000	-5.31473600
H	2.56556900	-2.86121200	-5.22859900
C	2.87544100	-4.60517300	2.25311600
H	2.02765900	-5.28953400	2.31138700
H	3.13478100	-4.33616100	3.27691300
H	3.71507900	-5.15367900	1.82586800
N	-3.18390200	1.39577100	-0.52621000
C	-2.67713100	2.64643700	-0.97691100
C	-2.34379700	3.62822000	-0.02871800
C	-4.49622600	1.17854900	-0.48643800
C	-5.47796900	2.24780100	-0.95509800
H	-4.89858100	3.04612800	-1.42088100
C	-2.48058400	2.87239300	-2.34981300
C	-1.63777000	5.07678600	-1.83134600
H	-1.23747500	6.02636500	-2.16446100
C	-2.77519000	1.77756100	-3.35095800
H	-3.54077100	1.12917700	-2.91702400
C	-1.81435200	4.83336000	-0.48032900
H	-1.54887800	5.59882500	0.23839700
C	-1.97032200	4.10047800	-2.75542800
H	-1.82182200	4.29218800	-3.81121900
C	-2.53717000	3.35918400	1.44791400
H	-3.37648800	2.66390700	1.54460800
C	-6.44862900	1.72176300	-2.00621000
H	-7.14448700	0.99164800	-1.59219800
H	-5.92689000	1.24306900	-2.83487500
H	-7.04205700	2.54034600	-2.41455700
C	-6.22479200	2.85498300	0.22766900
H	-6.89226900	3.64961700	-0.10665200
H	-5.54028800	3.28203000	0.95990100
H	-6.82981200	2.10522000	0.73961100
C	-1.30371100	2.67807900	2.03511200
H	-0.99099400	1.78445700	1.48584900
H	-1.47000800	2.39599500	3.07595400
H	-0.44695600	3.35203700	2.00274600
C	-1.52559300	0.92410300	-3.55857300
H	-0.73219900	1.51313100	-4.02306100
H	-1.72751000	0.06778100	-4.20362700
H	-1.12015800	0.54754300	-2.61379700
C	-3.31322600	2.29090000	-4.67706200
H	-4.18161300	2.93495100	-4.53530900
H	-3.61289200	1.45946400	-5.31502000
H	-2.56502300	2.86113900	-5.22883000
C	-2.87516200	4.60537700	2.25273400
H	-2.02727100	5.28960000	2.31103000
H	-3.13468400	4.33654100	3.27653100
H	-3.71464500	5.15397800	1.82530300
N	3.18431700	1.39522400	0.52635300
C	2.67777500	2.64598800	0.97704700
C	2.34464600	3.62775800	0.02875900
C	4.49660400	1.17786300	0.48611200
C	5.47864800	2.24689300	0.95464700
H	4.89948400	3.04543400	1.42034100
C	2.48110500	2.87201700	2.34990500
C	1.63860500	5.07648200	1.83125900
H	1.23841000	6.02613500	2.16428500
C	2.77563000	1.77729000	3.35119600
H	3.54122300	1.12882800	2.91739100
C	1.81536500	4.83300300	0.48026300
H	1.55006700	5.59848600	-0.23850900
C	1.97088700	4.10016500	2.75541200
H	1.82235100	4.29194300	3.81118800
C	2.53814400	3.35850700	-1.44783300
H	3.37735900	2.66307800	-1.54430600
C	6.44907500	1.72062000	2.00586200
H	7.14469300	0.99022300	1.59194500
H	5.92712100	1.24217100	2.83453000
H	7.04276200	2.53903300	2.41417200

C 6.22574600 2.85374200 -0.22811200
H 6.89347000 3.64817500 0.10619300
H 5.54143800 3.28097300 -0.96041900
H 6.83055000 2.10374900 -0.73996800
C 1.30465700 2.67747700 -2.03508400
H 0.99152400 1.78419400 -1.48551100
H 1.47115700 2.39488300 -3.07575800
H 0.44806900 3.35167000 -2.00322700
C 1.52600600 0.92387300 3.55883300
H 0.73247700 1.51304300 4.02291100
H 1.72781600 0.06781600 4.20427100
H 1.12082300 0.54692400 2.61409800
C 3.31359900 2.29081700 4.67724900
H 4.18204600 2.93476900 4.53543700
H 3.61315500 1.45947300 5.31537900
H 2.56540300 2.86121900 5.22885900
C 2.87645600 4.60448200 -2.25283800
H 2.02868100 5.28881800 -2.31144800
H 3.13612600 4.33538800 -3.27653100
H 3.71594500 5.15303800 -1.82536100

Small model (with dispersion), [{(^{MeMe}NacNac)Mg}$_2$(μ-O)]
Mg 1.79913400 0.00000500 0.00001800
O 0.00000000 -0.00000400 0.00003200
N 3.16228700 1.48309100 0.00000900
C 5.06556600 -0.00000400 -0.00001400
H 6.14605600 -0.00000700 -0.00002600
C 4.47238100 1.27232500 -0.00000300
C 5.40721400 2.44675900 -0.00000100
H 5.24043500 3.08067500 -0.87256200
Mg -1.79913400 0.00000200 -0.00000400
N -3.16228400 1.48309100 -0.00001900
C -5.06556500 -0.00000100 0.00001300
H -6.14605600 -0.00000100 0.00002800
C -4.47237900 1.27232800 -0.00000100
C -5.40720900 2.44676300 0.00000000
H -5.24045200 3.08065500 -0.87258200
N 3.16228000 -1.48308800 -0.00000200
C 4.47237500 -1.27233100 -0.00001100
C 5.40720100 -2.44676900 -0.00001600
H 5.24042000 -3.08067600 -0.87258300
N -3.16228200 -1.48308800 -0.00001100
C -4.47237700 -1.27232800 0.00000700
C -5.40720500 -2.44676500 0.00001900
H -5.24041900 -3.08067000 0.87258600
C -2.68298600 -2.85158600 -0.00002200
H -3.00747400 -3.41740700 -0.87992500
H -1.59264900 -2.85025400 -0.00003200
H -3.00745800 -3.41741500 0.87988200
C -2.68299100 2.85159000 -0.00003400
H -3.00745800 3.41741900 0.87987200
H -1.59265400 2.85026000 -0.00005000
H -3.00748600 3.41741000 -0.87993600
C 2.68299500 2.85159000 0.00002200
H 3.00747900 3.41740700 0.87993000
H 3.00747400 3.41742200 -0.87987700
H 1.59265800 2.85026200 0.00002600
C 2.68298000 -2.85158400 0.00000700
H 3.00746000 -3.41741800 -0.87989100
H 3.00745600 -3.41740300 0.87991600
H 1.59264300 -2.85024900 0.00000500
H 6.44675600 -2.13364400 -0.00002000
H 5.24042700 -3.08067400 0.87255300
H 6.44676700 2.13362800 -0.00001100
H 5.24044700 3.08065700 0.87257500
H -6.44675900 -2.13363800 0.00003100
H -5.24043900 -3.08067300 -0.87255000
H -6.44676300 2.13363400 0.00002500
H -5.24041700 3.08068500 0.87255400

Small model (without dispersion), [{(^{MeMe}NacNac)Mg}$_2$(μ-O)]
Mg 1.79950400 0.00000500 0.00000700
O 0.00000000 -0.00000500 0.00000100
N 3.16325400 1.48308200 0.00000700
C 5.06656300 -0.00000500 -0.00001400
H 6.14703800 -0.00000900 -0.00002600
C 4.47336700 1.27228600 -0.00000900
C 5.40817800 2.44677500 -0.00002400
H 5.24139000 3.08070900 -0.87256700

Mg -1.79950400 -0.00000100 -0.00000400
N -3.16324800 1.48308100 0.00000500
C -5.06656400 0.00000400 -0.00000700
H -6.14703800 0.00000500 -0.00001300
C -4.47336100 1.27229200 0.00000000
C -5.40816700 2.44678600 0.00000100
H -5.24138100 3.08073000 -0.87253400
N 3.16324500 -1.48308100 0.00001300
C 4.47335900 -1.27229300 -0.00000200
C 5.40816400 -2.44678700 -0.00000800
H 5.24138200 -3.08071800 -0.87255400
N -3.16325200 -1.48308000 -0.00000200
C -4.47336500 -1.27228600 -0.00000800
C -5.40817500 -2.44677700 -0.00001400
H -5.24140100 -3.08071700 0.87252800
C -2.68454100 -2.85179600 -0.00000500
H -3.00941800 -3.41746600 -0.87986800
H -1.59421400 -2.85106100 0.00000300
H -3.00942900 -3.41747300 0.87985000
C -2.68453200 2.85179500 0.00001500
H -3.00941700 3.41746500 0.87987500
H -1.59420500 2.85105600 0.00002100
H -3.00940700 3.41747500 -0.87984300
C 2.68454600 2.85179800 0.00001200
H 3.00944000 3.41747300 0.87986600
H 3.00941600 3.41747000 -0.87985200
H 1.59421800 2.85106600 0.00002700
C 2.68452900 -2.85179500 0.00003100
H 3.00941400 -3.41748400 -0.87981700
H 3.00940300 -3.41745500 0.87990200
H 1.59420100 -2.85105500 0.00002500
H 6.44774400 -2.13378000 -0.00000800
H 5.24138400 -3.08073200 0.87252800
H 6.44775700 2.13376300 -0.00003700
H 5.24141200 3.08071900 0.87251600
H -6.44775400 -2.13376600 -0.00001900
H -5.24139200 -3.08071500 -0.87255500
H -6.44774700 2.13377800 -0.00000800
H -5.24139300 3.08071700 0.87254800

LMgSMgL species

Mg-S-Mg full, [{(^{iPrDip}NacNac)Mg}$_2$(μ-S)] **4**
Mg -2.10450900 -0.00751900 -0.15450200
N -3.39222700 -1.47003100 0.32747300
N -3.52775300 1.32929200 -0.62781900
C -4.71761500 -1.30672700 0.31238500
C -5.35311700 -0.12830100 -0.08768000
H -6.43178500 -0.16923900 -0.06216400
C -4.82462700 1.11041100 -0.48774400
C -2.80181100 -2.67829200 0.78758800
C -2.61814800 -2.89197400 2.16573800
C -1.99327700 -4.06389400 2.57761300
H -1.84954600 -4.24469900 3.63615100
C -1.54171600 -4.99633300 1.65910800
H -1.05648900 -5.90313300 1.99891000
C -1.70859900 -4.76421600 0.30507800
H -1.34866600 -5.49480200 -0.40847800
C -2.34179500 -3.61402100 -0.15547000
C -3.04275300 -1.84437400 3.17232100
H -3.83038700 -1.24074800 2.71444500
C -3.61262900 -2.43424900 4.45361100
H -4.00778000 -1.64509700 5.09312600
H -2.85482200 -2.96282800 5.03293500
H -4.42137200 -3.13628600 4.24850100
C -1.87214800 -0.90927700 3.47374800
H -1.46472700 -0.44263000 2.57128400
H -1.05178200 -1.45962200 3.93934300
H -2.16614000 -0.10954600 4.15508100
C -2.51530800 -3.35425700 -1.63715400
H -3.41163200 -2.73845600 -1.75549300
C -2.71177700 -4.62291600 -2.45415800
H -3.50534400 -5.24936900 -2.04714400
H -1.80114900 -5.22269200 -2.49250600
H -2.97176000 -4.37569600 -3.48311700
C -1.33654000 -2.56053900 -2.19458400
H -0.40603700 -3.11976900 -2.08518200
H -1.16448900 -1.60750300 -1.68412500
H -1.47593600 -2.33687800 -3.25318500

C	-5.63176500	-2.45412300	0.72751100
H	-5.00806300	-3.21160200	1.20522300
C	-6.26819700	-3.09520500	-0.50159600
H	-6.89529000	-3.93961100	-0.21450800
H	-5.51632800	-3.46224100	-1.19980900
H	-6.89476400	-2.37947400	-1.03562600
C	-6.69015800	-2.03798100	1.74137700
H	-7.21142100	-2.91685300	2.12185800
H	-7.44287500	-1.38083200	1.30560500
H	-6.25208500	-1.51568600	2.59188500
C	-5.82610700	2.21391800	-0.78797100
H	-5.26091500	3.14727800	-0.84485600
C	-6.46640000	1.97507900	-2.15157100
H	-7.13735600	2.79349900	-2.41348800
H	-7.04878800	1.05245500	-2.15180000
H	-5.71636900	1.89201700	-2.93741000
C	-6.88226300	2.37603400	0.29727400
H	-7.58892800	1.54582600	0.30877800
H	-7.45866300	3.28629700	0.12955500
H	-6.43563800	2.44062300	1.28923400
C	-2.97874100	2.60317700	-0.95491500
C	-2.53693300	2.84141500	-2.26741200
C	-1.84940100	4.01996400	-2.53535900
H	-1.49329900	4.20568200	-3.54249500
C	-1.59570600	4.94622200	-1.54064300
H	-1.05097400	5.85443000	-1.76704600
C	-2.03335100	4.69887200	-0.25295900
H	-1.82192200	5.41676200	0.53170100
C	-2.72245500	3.53450300	0.06688400
C	-2.72959400	1.82258300	-3.37114800
H	-3.42603900	1.06220800	-3.00448600
C	-3.33184100	2.43324100	-4.63058700
H	-2.65025600	3.14916300	-5.09152500
H	-4.26310500	2.95953800	-4.42208300
H	-3.53869100	1.66228600	-5.37340200
C	-1.40796800	1.12695300	-3.69297900
H	-0.94589700	0.69420000	-2.80204400
H	-0.68655700	1.83933500	-4.09948900
H	-1.54576200	0.33613500	-4.43239800
C	-3.13973800	3.29710900	1.50309100
H	-3.65562100	2.33326400	1.55344200
C	-4.11515500	4.36843200	1.98101200
H	-4.98328100	4.45069600	1.32683100
H	-3.63557800	5.34806600	2.00929800
H	-4.47204400	4.15087600	2.98851900
C	-1.92586900	3.22058100	2.42631100
H	-1.40742300	4.18018000	2.46922500
H	-1.20042200	2.48156700	2.08037300
H	-2.22772600	2.96555700	3.44375400
S	-0.01041500	0.77356200	0.03562200
Mg	2.10076900	0.01893700	0.10870700
N	3.40351200	-1.43642400	-0.35350300
N	3.51319800	1.36065000	0.59817700
C	4.72833500	-1.26882900	-0.31580700
C	5.35335300	-0.09424500	0.11001700
H	6.43301400	-0.13436400	0.11147200
C	4.81340900	1.13652400	0.51991600
C	2.82697600	-2.65441700	-0.80583000
C	2.63909700	-2.87497600	-2.18202600
C	2.03293100	-4.05870200	-2.58802700
H	1.88737000	-4.24503200	-3.64540600
C	1.60190400	-4.99558100	-1.66424400
H	1.13072600	-5.91175300	-1.99864300
C	1.77021300	-4.75572800	-0.31157000
H	1.42487100	-5.48966400	0.40575300
C	2.38626600	-3.59386500	0.14305400
C	3.03776200	-1.82027200	-3.19136900
H	3.81323800	-1.19939400	-2.73587300
C	3.61574400	-2.39809900	-4.47429200
H	3.98731000	-1.60088100	-5.11792400
H	2.86880200	-2.94772100	-5.04812600
H	4.44322200	-3.07850100	-4.27132000
C	1.84546000	-0.91152700	-3.48906600
H	1.41683600	-0.47103800	-2.58284400
H	1.04378800	-1.47563300	-3.97031000
H	2.12350600	-0.09336200	-4.15478100
C	2.56256000	-3.32670500	1.62346300
H	3.45504500	-2.70425800	1.73645800
C	2.77110600	-4.59089700	2.44433400

H 3.56663200 -5.21429200 2.03630700
H 1.86440500 -5.19611900 2.48967300
H 3.03431500 -4.33767100 3.47098200
C 1.38112600 -2.53786700 2.18286900
H 0.45233100 -3.10066800 2.07630800
H 1.20571500 -1.58543300 1.67331900
H 1.52203100 -2.31324900 3.24117300
C 5.65442500 -2.40681600 -0.73056800
H 5.03910400 -3.17237600 -1.20634100
C 6.30620900 -3.03967700 0.49450700
H 6.94238000 -3.87586600 0.20336000
H 5.56354100 -3.41647700 1.19728400
H 6.92695000 -2.31648200 1.02534500
C 6.70223600 -1.97372600 -1.74868600
H 7.24042700 -2.84308900 -2.12746300
H 7.44161200 -1.29960000 -1.31599800
H 6.25217400 -1.46280200 -2.59978900
C 5.80910700 2.22312800 0.89081100
H 5.23316600 3.13187600 1.08210900
C 6.54397800 1.85467700 2.17527400
H 7.19093300 2.67088800 2.49765400
H 7.16941200 0.97267900 2.03073200
H 5.85085100 1.63542800 2.98686700
C 6.78893200 2.51901000 -0.23818000
H 7.47436400 1.68760900 -0.40574100
H 7.39223100 3.39515600 0.00116100
H 6.27486000 2.71385900 -1.17892400
C 2.94986500 2.62299300 0.94475600
C 2.48439500 2.83128100 2.25352800
C 1.76898600 3.99125200 2.52917500
H 1.39453900 4.15349800 3.53396500
C 1.50892900 4.92620500 1.54463600
H 0.93803100 5.81713600 1.77531000
C 1.97746100 4.71184200 0.26180900
H 1.76505100 5.43899600 -0.51389800
C 2.70071100 3.56997900 -0.06345500
C 2.69888500 1.81192200 3.35224300
H 3.36135300 1.03310000 2.96143600
C 3.37662700 2.42469200 4.57239800
H 2.73439000 3.16435800 5.05222900
H 4.30792300 2.92594000 4.30880200
H 3.60234600 1.66058000 5.31701500
C 1.38011600 1.14781100 3.74266900
H 0.87682500 0.70830500 2.87839600
H 0.68946400 1.87813900 4.17002400
H 1.53759900 0.36674800 4.48849200
C 3.15367400 3.36277000 -1.49320700
H 3.77470000 2.46274200 -1.52547100
C 4.00570700 4.52671600 -1.98777400
H 4.84922400 4.72224000 -1.32529000
H 3.42157100 5.44584500 -2.05047200
H 4.39931000 4.32452900 -2.98466300
C 1.95885100 3.13368300 -2.41606700
H 1.31737500 4.01652000 -2.44673900
H 1.33368200 2.30647400 -2.07335700
H 2.28679200 2.92577500 -3.43624200

Small model (with dispersion), [{(^{MeMe}NacNac)Mg}$_2$(μ-S)]
Mg -1.48294000 -1.42146200 -0.52850400
N -0.91156000 0.30096500 -1.40971300
N -3.29236500 -0.76102600 0.02709800
C -1.58773800 1.43153100 -1.26477400
C -2.84652400 1.51892100 -0.64419000
H -3.27650700 2.50996900 -0.62464100
C -3.64562900 0.51684900 -0.07079700
C -1.00131200 2.71287600 -1.78184900
H -0.86462900 2.67314300 -2.86382200
C -4.97595100 0.95229600 0.46983600
H -5.05381400 0.74158800 1.53785500
S 0.00001200 -3.06373500 -0.00004900
Mg 1.48297100 -1.42148800 0.52846400
N 0.91155300 0.30090900 1.40971500
N 3.29239500 -0.76100700 -0.02709200
C 1.58770200 1.43149400 1.26479200
C 2.84648900 1.51892300 0.64421500
H 3.27644500 2.50998200 0.62467500
C 3.64562300 0.51687700 0.07081700
C 1.00124100 2.71281800 1.78187900
H 0.86455000 2.67306700 2.86385000

C 4.97593800 0.95236400 -0.46980000
H 5.79063300 0.40590600 0.00791100
C -4.21513000 -1.68271800 0.66427300
H -5.16429600 -1.77319200 0.12635100
H -3.76904900 -2.67640200 0.70383500
H -4.45597100 -1.39949100 1.69380500
C 0.37980900 0.34970300 -2.07161300
H 0.77271700 -0.66553500 -2.16551900
H 0.32687500 0.76654500 -3.08222900
H 1.11975800 0.94102400 -1.51748100
C -0.37981600 0.34960700 2.07161800
H -0.32688600 0.76642700 3.08224500
H -1.11977500 0.94093100 1.51750300
H -0.77270600 -0.66563900 2.16550200
C 4.21519700 -1.68266700 -0.66426000
H 5.16436300 -1.77310600 -0.12633200
H 3.76915300 -2.67636700 -0.70382400
H 4.45603400 -1.39943300 -1.69379100
H -5.14275900 2.01423800 0.31890300
H -5.79063400 0.40580800 -0.00786100
H -1.63142100 3.56654100 -1.55058500
H -0.01056300 2.88731600 -1.35689000
H 5.14270900 2.01431200 -0.31887000
H 5.05382300 0.74165400 -1.53781700
H 1.63133300 3.56650100 1.55063300
H 0.01049200 2.88724000 1.35691400

Small model (without dispersion), [{(^{MeMe}NacNac)Mg}$_2$(μ-S)]
Mg -1.80554500 -0.89846900 -0.16509300
N -2.08667300 0.95368900 -0.89182300
N -3.69981600 -1.16939900 0.43126800
C -3.27112800 1.55935700 -0.87212600
C -4.43678200 0.99764300 -0.33322100
H -5.31371600 1.62577400 -0.39282900
C -4.64616700 -0.25429800 0.26887800
C -3.40055600 2.93545900 -1.45698100
H -3.09509200 2.94755500 -2.50446300
C -6.03889000 -0.54356300 0.74613600
H -6.05107200 -0.74287800 1.81893400
S -0.00000400 -2.25382400 0.00003200
Mg 1.80553600 -0.89846200 0.16512200
N 2.08669000 0.95369900 0.89182900
N 3.69979200 -1.16940800 -0.43127800
C 3.27115400 1.55935100 0.87212300
C 4.43679600 0.99762000 0.33321300
H 5.31374000 1.62573700 0.39281900
C 4.64615700 -0.25432100 -0.26889400
C 3.40060200 2.93545300 1.45697300
H 3.09513700 2.94755900 2.50445400
C 6.03887000 -0.54360300 -0.74617000
H 6.43912900 -1.43821100 -0.26652600
C -4.05197700 -2.42467600 1.06991100
H -4.81152200 -2.98307900 0.51358600
H -3.16689800 -3.05681300 1.13672500
H -4.43349500 -2.28929700 2.08680600
C -0.97157400 1.65138400 -1.50134700
H -0.06486400 1.05140900 -1.39277400
H -1.11301600 1.82482800 -2.57336500
H -0.76973200 2.62450700 -1.04249400
C 0.97160100 1.65141400 1.50134800
H 1.11304600 1.82486400 2.57336500
H 0.76977400 2.62453700 1.04248900
H 0.06488300 1.05145200 1.39277900
C 4.05192600 -2.42468300 -1.06993900
H 4.81147600 -2.98310000 -0.51363500
H 3.16683800 -3.05680900 -1.13674100
H 4.43342500 -2.28929900 -2.08684100
H -6.71318000 0.28310500 0.54511700
H -6.43915700 -1.43816100 0.26648000
H -4.41961200 3.30417100 -1.39420900
H -2.74917600 3.64345600 -0.94159300
H 6.71317600 0.28305300 -0.54515100
H 6.05103700 -0.74290900 -1.81897000
H 4.41966400 3.30415000 1.39419900
H 2.74923300 3.64345700 0.94158000

LMgSeMgL species

MgSeMg full, [{(^{iPrDip}NacNac)Mg}$_2$(μ-Se)] **5**

Mg	2.15918400	-0.00819200	-0.11727000
N	3.60868700	1.30320600	-0.60032200
C	4.90183500	1.05316600	-0.49319500
Se	-0.00000200	0.97817200	0.00001400
N	3.42389200	-1.48730300	0.37042800
C	5.40807500	-0.18783400	-0.07065600
H	6.48636900	-0.25117300	-0.05565500
C	4.75263300	-1.34720200	0.34985800
C	5.92745200	2.11612800	-0.85009300
H	5.37720800	3.04458500	-1.02228100
C	6.63885100	1.74467600	-2.14708000
H	7.23355700	0.83883400	-2.02076700
H	7.31150200	2.54285600	-2.46187800
H	5.93195500	1.56268900	-2.95584500
C	6.92749500	2.36382100	0.27253900
H	7.60114000	1.51717600	0.40805300
H	6.43044100	2.54253500	1.22548300
H	7.54311200	3.23524000	0.04758900
C	5.64906200	-2.50327500	0.77960700
H	5.01239800	-3.25273400	1.25275200
C	6.29775200	-3.15618200	-0.43655600
H	6.93939500	-2.44934500	-0.96454100
H	5.55325800	-3.51920100	-1.14462500
H	6.91220700	-4.00519500	-0.13604200
C	6.69658800	-2.08924400	1.80588300
H	7.20956700	-2.96905300	2.19536700
H	6.25021900	-1.56239200	2.64917300
H	7.45664500	-1.43596600	1.37709500
C	3.07335800	2.57695400	-0.95253400
C	2.59694000	2.78140700	-2.25851100
C	1.88950200	3.94604300	-2.53600000
H	1.50628700	4.10344000	-3.53825600
C	1.65071400	4.89243800	-1.55716700
H	1.08601700	5.78694600	-1.78868100
C	2.13425300	4.68508100	-0.27885000
H	1.93998900	5.42166300	0.49283800
C	2.84599600	3.53679900	0.04984000
C	2.79704700	1.75869700	-3.35744600
H	3.42805400	0.95772100	-2.95883000
C	1.46808300	1.13807600	-3.78288600
H	0.93602800	0.69962600	-2.93586200
H	1.61932200	0.36457000	-4.53789200
H	0.80674500	1.89338500	-4.21299800
C	3.51681600	2.35929000	-4.56025700
H	2.90516400	3.12135200	-5.04510500
H	3.73394400	1.59438300	-5.30668000
H	4.45700000	2.83153100	-4.27585700
C	3.32052000	3.34891700	1.47584100
H	3.87448500	2.40697500	1.52931600
C	2.14715000	3.25009900	2.44850000
H	1.57277700	4.17843400	2.46186500
H	2.50086800	3.06726500	3.46477600
H	1.45340000	2.45419200	2.17155800
C	4.27020100	4.46829200	1.89213000
H	5.10311200	4.57436800	1.19686400
H	4.68109100	4.28270300	2.88539200
H	3.75277800	5.42805200	1.92815100
C	2.81740600	-2.69204100	0.81902700
C	2.36790400	-3.62318000	-0.13409000
C	1.72893700	-4.77486600	0.31432200
H	1.37923300	-5.50347500	-0.40621500
C	1.54358800	-5.01129500	1.66537000
H	1.05560600	-5.92019100	1.99541900
C	1.98019600	-4.08102000	2.59310200
H	1.82081700	-4.26464300	3.64897100
C	2.61207800	-2.90851500	2.19351300
C	2.55879000	-3.35677300	-1.61290000
H	3.46223100	-2.74918000	-1.71938600
C	1.39370700	-2.54754800	-2.17778700
H	0.45464700	-3.09418300	-2.07610000
H	1.54310000	-2.32237700	-3.23478600
H	1.23198600	-1.59190500	-1.66797600
C	2.75071800	-4.62240600	-2.43558100
H	3.53281700	-5.26011000	-2.02388100
H	3.02484500	-4.37150100	-3.45992100
H	1.83439600	-5.21221400	-2.48803500
C	3.02229900	-1.86314900	3.20815100
H	3.81129700	-1.25482400	2.75893000
C	1.84425100	-0.93444000	3.50049900

H 1.43289000 -0.48248700 2.59217400
H 2.13111700 -0.12261100 4.17048300
H 1.02785900 -1.48527500 3.97262900
C 3.58240400 -2.45470300 4.49288500
H 2.82206900 -2.99139900 5.06131900
H 3.96486900 -1.66580900 5.14036600
H 4.39833200 -3.14997200 4.29342500
Mg -2.15918600 -0.00819700 0.11727800
N -3.60870400 1.30318300 0.60033300
C -4.90184800 1.05314900 0.49315200
N -3.42387900 -1.48732000 -0.37042800
C -5.40807600 -0.18785200 0.07060200
H -6.48636900 -0.25119200 0.05557400
C -4.75262100 -1.34722200 -0.34988500
C -5.92747300 2.11611400 0.85001900
H -5.37723600 3.04457800 1.02218600
C -6.63885800 1.74468200 2.14702000
H -7.23355500 0.83883200 2.02073100
H -7.31151400 2.54286300 2.46180900
H -5.93195200 1.56271900 2.95578200
C -6.92752300 2.36377700 -0.27261100
H -7.60116800 1.51712700 -0.40810400
H -6.43047700 2.54247300 -1.22556300
H -7.54314300 3.23519800 -0.04767600
C -5.64903800 -2.50330600 -0.77963100
H -5.01236500 -3.25276400 -1.25276600
C -6.29772200 -3.15620900 0.43653900
H -6.93937600 -2.44937300 0.96451300
H -5.55322400 -3.51920900 1.14461500
H -6.91216500 -4.00523400 0.13603500
C -6.69656700 -2.08930100 -1.80591200
H -7.20953400 -2.96912100 -2.19538600
H -6.25020400 -1.56245500 -2.64920900
H -7.45663400 -1.43602800 -1.37713400
C -3.07338200 2.57693300 0.95254500
C -2.59695500 2.78138000 2.25852000
C -1.88952200 3.94601900 2.53601200
H -1.50630100 4.10341200 3.53826700
C -1.65074700 4.89242100 1.55718300
H -1.08605500 5.78693200 1.78869900
C -2.13429400 4.68506900 0.27886800
H -1.94003700 5.42165600 -0.49281700
C -2.84603200 3.53678500 -0.04982500
C -2.79705500 1.75866600 3.35745300
H -3.42805100 0.95768300 2.95883300
C -1.46808600 1.13805900 3.78290300
H -0.93602300 0.69961200 2.93588300
H -1.61932300 0.36455400 4.53791000
H -0.80675900 1.89337500 4.21301600
C -3.51683700 2.35924900 4.56026100
H -2.90519700 3.12132100 5.04511000
H -3.73395600 1.59434000 5.30668400
H -4.45702700 2.83147600 4.27586000
C -3.32055700 3.34890500 -1.47582500
H -3.87451800 2.40696000 -1.52930400
C -2.14718500 3.25009200 -2.44848400
H -1.57281900 4.17843000 -2.46184900
H -2.50090100 3.06725100 -3.46475900
H -1.45342900 2.45419100 -2.17153900
C -4.27024100 4.46827500 -1.89211800
H -5.10315600 4.57434700 -1.19685500
H -4.68112700 4.28268400 -2.88538100
H -3.75282300 5.42803800 -1.92813800
C -2.81738400 -2.69205700 -0.81901700
C -2.36787100 -3.62318500 0.13410600
C -1.72889900 -4.77487100 -0.31429900
H -1.37918700 -5.50347100 0.40624300
C -1.54355500 -5.01131100 -1.66534500
H -1.05557000 -5.92020800 -1.99538800
C -1.98017400 -4.08104800 -2.59308400
H -1.82080000 -4.26467900 -3.64895100
C -2.61206000 -2.90854200 -2.19350200
C -2.55875500 -3.35676700 1.61291400
H -3.46219800 -2.74917400 1.71939500
C -1.39367500 -2.54753600 2.17779700
H -0.45461200 -3.09416700 2.07610800
H -1.54306600 -2.32236500 3.23479600
H -1.23195800 -1.59189200 1.66798500
C -2.75068200 -4.62239400 2.43560500

H -3.53277900 -5.26010200 2.02390900
H -3.02481000 -4.37148200 3.45994200
H -1.83435900 -5.21220100 2.48806400
C -3.02229000 -1.86318500 -3.20814500
H -3.81130600 -1.25487700 -2.75893600
C -1.84425800 -0.93445200 -3.50047800
H -1.43292300 -0.48248500 -2.59214900
H -2.13112900 -0.12263200 -4.17047100
H -1.02784500 -1.48527200 -3.97258900
C -3.58236600 -2.45474900 -4.49288800
H -2.82201000 -2.99141800 -5.06131900
H -3.96485100 -1.66586300 -5.14036700
H -4.39827500 -3.15004400 -4.29343800

Small model (with dispersion), [{(^{MeMe}NacNac)Mg}$_2$(μ-Se)]
Mg -1.49769600 -1.12255900 0.53799600
N -3.29537100 -0.43017500 -0.01541900
C -3.62533400 0.85374900 0.08144500
Se 0.00001100 -2.93702200 0.00003100
N -0.89037900 0.58989800 1.41281600
C -2.80534300 1.84199000 0.64957200
H -3.21686800 2.84083500 0.62963900
C -1.54686700 1.73240800 1.26680700
C -4.94942400 1.31225300 -0.45508800
H -5.77206400 0.78108100 0.02619600
C -0.93616300 3.00382200 1.78043000
H 0.05708100 3.15955200 1.35385100
Mg 1.49774700 -1.12259600 -0.53798100
N 3.29540400 -0.43014800 0.01540900
C 3.62532300 0.85378800 -0.08146600
N 0.89037300 0.58983400 -1.41282800
C 2.80530000 1.84199500 -0.64960400
H 3.21679100 2.84085500 -0.62968000
C 1.54682500 1.73236700 -1.26683300
C 4.94939900 1.31234100 0.45506000
H 5.03464400 1.10139800 1.52246900
C 0.93607400 3.00375600 -1.78045600
H -0.05716700 3.15945900 -1.35385700
C -4.23685700 -1.33691800 -0.64678000
H -3.80755300 -2.33798500 -0.68685800
H -5.18459000 -1.41018000 -0.10385600
H -4.47766300 -1.05070100 -1.67544400
C 0.40206300 0.61869700 2.07416700
H 0.35551300 1.03618400 3.08478400
H 0.77934000 -0.40258700 2.16827600
H 1.14990600 1.19958600 1.51962300
C -0.40207400 0.61859200 -2.07417000
H -0.35554400 1.03607000 -3.08479100
H -0.77932500 -0.40270400 -2.16826500
H -1.14992800 1.19946800 -1.51962600
C 4.23692500 -1.33685100 0.64677500
H 3.80767700 -2.33794300 0.68682400
H 5.18467700 -1.41004900 0.10387600
H 4.47768800 -1.05064100 1.67545200
H -5.03463900 1.10133500 -1.52250500
H -5.09660900 2.37723800 -0.30520800
H 5.09653300 2.37733600 0.30520100
H 5.77205600 0.78121900 -0.02625300
H 1.55100800 3.86819000 -1.54826300
H 0.79890200 2.96374600 -2.86234600
H -1.55112000 3.86823400 1.54821800
H -0.79901300 2.96382400 2.86232300

Small model (without dispersion), [{(^{MeMe}NacNac)Mg}$_2$(μ-Se)]
Mg -1.50347300 -1.11708300 0.52645400
N -3.30617500 -0.43859700 -0.02741500
C -3.64653100 0.84233400 0.07240400
Se 0.00000300 -2.92975800 0.00000600
N -0.90755900 0.59936800 1.40120200
C -2.83391700 1.83624600 0.64139300
H -3.25417700 2.83148400 0.62417700
C -1.57485600 1.73615300 1.25892100
C -4.97632900 1.29035500 -0.45907900
H -5.79263400 0.75285400 0.02601900
C -0.97692700 3.01133800 1.77827800
H 0.01577000 3.17775900 1.35462900
Mg 1.50348300 -1.11709000 -0.52645500
N 3.30618000 -0.43858900 0.02741400
C 3.64652900 0.84234200 -0.07241000

N	0.90755600	0.59935900	-1.40120000
C	2.83390800	1.83624900	-0.64139800
H	3.25416300	2.83149000	-0.62418600
C	1.57484600	1.73614800	-1.25892200
C	4.97632700	1.29037200	0.45906700
H	5.06471300	1.07833200	1.52596500
C	0.97690800	3.01132800	-1.77828000
H	-0.01578900	3.17774400	-1.35462800
C	-4.24285700	-1.35255500	-0.65555400
H	-3.80618600	-2.35034000	-0.69713800
H	-5.18805400	-1.43295500	-0.10921100
H	-4.48961900	-1.06813600	-1.68326900
C	0.38319800	0.63730700	2.06522600
H	0.33123700	1.05178900	3.07682200
H	0.76839000	-0.38122000	2.15817500
H	1.12796900	1.22492100	1.51368800
C	-0.38320200	0.63728900	-2.06522300
H	-0.33124500	1.05177300	-3.07681800
H	-0.76838800	-0.38124000	-2.15817100
H	-1.12797600	1.22489900	-1.51368300
C	4.24287100	-1.35254200	0.65554900
H	3.80620200	-2.35032700	0.69714500
H	5.18806100	-1.43294400	0.10919500
H	4.48964500	-1.06811600	1.68325800
H	-5.06471700	1.07830200	-1.52597400
H	-5.13155700	2.35414000	-0.30880500
H	5.13155300	2.35415500	0.30878100
H	5.79263300	0.75286600	-0.02602300
H	1.59922900	3.87094600	-1.54791500
H	0.84200400	2.96887000	-2.86041300
H	-1.59925400	3.87095100	1.54791100
H	-0.84202600	2.96888100	2.86041200

5 References

1 Kuhn, N.; Kratz, T. Synthesis of Imidazol-2-ylidenes by Reduction of Imidazole-2(3*H*)-thiones. *Synthesis* **1993**, 561–562.

2 Burnett, S.; Bourne, C.; Slawin, A. M. Z.; van Mourik, T.; Stasch, A. Umpolung of an Aliphatic Ketone to a Magnesium Ketone-1,2-diide Complex with Vicinal Dianionic Charge. *Angew. Chem. Int. Ed.* **2022**, *61*, e20220447.

3 Kumar, R.; Kumar, S.; Pandey, M. K.; Kashid, V. S.; Radhakrishna, L.; Balakrishna, M. S. Synthesis of Phosphine Chalcogenides Under Solvent-Free Conditions Using a Rotary Ball Mill. *Eur. J. Inorg. Chem.* **2018**, 1028–1037.

4 Fulmer, G. R.; Miller, A. J. M.; Sherden, N. H.; Gottlieb, H. E.; Nudelman, A.; Stoltz, B. M.; Bercaw, J. E.; Goldberg, K. I. NMR Chemical Shifts of Trace Impurities: Common Laboratory Solvents, Organics, and Gases in Deuterated Solvents Relevant to the Organometallic Chemist. *Organometallics* **2010**, *29*, 2176–2179.

5 Hesse, M.; Meier, H.; Zeeh, B.; *Spektroskopische Methoden in der organischen Chemie*, 4. Auflage, Georg Thieme Verlag, Stuttgart, New York, 1991.

6 *CrystalClear-SM Exper*t v2.1. Rigaku Americas, The Woodlands, Texas, USA, and Rigaku Corporation, Tokyo, Japan, 2015.

7 *CrysAlisPro* v1.171.38.8d. Rigaku Oxford Diffraction, Rigaku Corporation, Oxford, U.K. 2015.

8 Sheldrick, G. M. *SHELXT* – Integrated space-group and crystal-structure determination. *Acta Cryst.* **2015**, *A71*, 3–8.

9 Sheldrick, G. M. Crystal structure refinement with SHELXL. *Acta Cryst.* **2015**, *C71*, 3–8.

10 Zhao, Y.; Truhlar, D. G. The M06 suite of density functionals for main group thermochemistry, thermochemical kinetics, noncovalent interactions, excited states, and transition elements: two new functionals and systematic testing of four M06-class functionals and 12 other functionals. *Theor. Chem. Acc.* **2008**, *120*, 215–241.

11 Grimme, S.; Antony, J.; Ehrlich, S.; Krieg, H. A consistent and accurate *ab initio* parametrization of density functional dispersion correction (DFT-D) for the 94 elements H-Pu. *J. Chem. Phys.* **2010**, *132*, 154104.

12 Weigend, F.; Ahlrichs, R. Balanced basis sets of split valence, triple zeta valence and quadruple zeta valence quality for H to Rn: Design and assessment of accuracy. *Phys. Chem. Chem. Phys.* **2005**, *7*, 3297–3305.

13 Reed, A. E.; Weinstock, R. B.; Weinhold, F. Natural population analysis. *J. Chem. Phys.* **1985**, *83*, 735–746.

14 Gaussian 16, Revision C.01, Frisch, M. J.; Trucks, G. W.; Schlegel, H. B.; Scuseria, G. E.; Robb, M. A.; Cheeseman, J. R.; Scalmani, G.; Barone, V.; Petersson, G. A.; Nakatsuji, H.; Li, X.; Caricato, M; Marenich, A. V.; Bloino, J.; Janesko, B.G.; Gomperts, R.; Mennucci, B.; Hratchian, H.P.; Ortiz, J.V.; Izmaylov, A.F.; Sonnenberg, J.L.; Williams-Young, D.; Ding, F.; Lipparini, F.; Egidi, F.; Goings, J.; Peng, B.; Petrone, A.; Henderson, T.; Ranasinghe, D.; Zakrzewski, V.G.; Gao, J.; Rega, N.; Zheng, G.; Liang, W.; Hada, M.; Ehara, M.; Toyota, K.; Fukuda, R.; Hasegawa, J.; Ishida, M.; Nakajima, T.; Honda, Y.; Kitao, O.; Nakai, H.; Vreven, T.; Throssell, K.; Montgomery Jr., J.A.; Peralta, J.E.; Ogliaro, F.; Bearpark, M.J.; Heyd, J.J.; Brothers, E.N.; Kudin, K.N.; Staroverov, V.N.; Keith, T.A.; Kobayashi, R.; Normand, J.; Raghavachari, K.; Rendell, A.P.; Burant, J.C.; Iyengar, S.S.; Tomasi, J.; Cossi, M.; Millam, J.M.; Klene, M.; Adamo, C.; Cammi, R.; Ochterski, J.W.; Martin, R.L.; Morokuma, K.; Farkas, O.; Foresman, J.B.; Fox, D.J. Gaussian, Inc., Wallingford CT, 2016.

15 AIMAll (Version 19.10.12), Keith, T. A. TK Gristmill Software, Overland Park KS, USA, 2019 (aim.tkgristmill.com).
